# Teaching Patient Handoffs to Medical Students in Obstetrics and Gynecology: Simulation Curriculum and Assessment Tool

**DOI:** 10.15766/mep_2374-8265.10479

**Published:** 2016-10-02

**Authors:** Celeste S. Royce, Katharyn Meredith Atkins, Monica Mendiola, Hope Ricciotti

**Affiliations:** 1 Instructor, Department of Obstetrics, Gynecology and Reproductive Gynecology, Beth Israel Deaconess Medical Center and Harvard Medical School; 2 Assistant Professor, Department of Obstetrics, Gynecology and Reproductive Gynecology, Beth Israel Deaconess Medical Center and Harvard Medical School; 3 Associate Professor, Department of Obstetrics, Gynecology and Reproductive Gynecology, Beth Israel Deaconess Medical Center and Harvard Medical School

**Keywords:** Simulation, Miscarriage, Spontaneous Abortion, Handoff, Intern, Handover

## Abstract

**Introduction:**

Patient handoffs, the communications required for the safe transfer of patient care, are known to be a common source of medical errors. Simulation exercises are effective techniques for teaching the procedures and patient interaction skills involved in a handoff. We developed a teaching tool that allows students to individually interact with a simulated patient, develop a treatment plan, and practice a handoff to another provider.

**Methods:**

The curriculum is a flexible instructional tool to teach patient handoffs in the context of a simulated obstetric emergency for learners at the clerkship through first-year obstetrics and gynecology resident levels. The curriculum secondarily teaches management of first-trimester bleeding with acute blood loss and can be adapted to allow advanced learners to practice obtaining informed consent. To evaluate this simulation for educational effectiveness, we developed a faculty observation assessment tool.

**Results:**

The simulation assessments for history taking, fund of knowledge, and interpersonal skills were predictive of subsequent clerkship clinical grades. Eighty percent of students agreed the exercise was realistic, 95% agreed it was relevant to the clinical curriculum, 90% agreed the simulation taught handoff skills, and 73% agreed the simulation increased confidence in handoff skills. Students uniformly found the curriculum to be relevant, realistic, and effective at teaching handoff skills.

**Discussion:**

Use of this curriculum has the potential to improve students’ communication skills, handoff performance, and confidence during an obstetrics and gynecology clerkship. The assessment tool may allow early identification of students in need of improvement in communication skills.

## Educational Objectives

By the end of the simulation session, learners will be able to:

1. Describe the necessary components of a successful patient handoff.
2. Give and receive a patient handoff.
3. Understand the diagnosis and management of spontaneous abortion with significant blood loss.

## Introduction

Patient handoffs are a common source of medical errors.^1–4^ Duty hour restrictions currently in place for trainees effectively increase the number of patient handoffs that must occur to comply with these limitations.^4,5^ Skills for giving and receiving patient handoffs are not standardized and not routinely taught in medical school.^6–11^ These skills are, however, widely recognized as essential for patient care and have been incorporated into the entrustable professional activities (EPAs) expected of graduating medical students by the Association of American Medical Colleges (AAMC).^12^

We developed a simulation to allow students and trainees to practice patient handoffs in the context of an evolving clinical scenario involving patient management. This curriculum was created to allow third-year medical students to practice patient handoffs for obstetric and gynecologic patients. It gives students an opportunity to practice independent management of a common gynecological emergency, a first-trimester pregnancy loss with significant hemorrhage. The inclusion of a patient management component was designed to provide a more immersive and realistic experience for students, which has been demonstrated to improve clinical skill acquisition.^13^

Medical students are traditionally expected to interview patients, take a medical history, perform a physical exam, and then present this information to a supervising physician, often a resident or attending physician on ward rounds. This allows medical students to function at the reporter level in the RIME (reporter, interpreter, manager, educator) framework of medical education and professional development, which roughly corresponds to third-year students, fourth-year students, intern/junior residents, and senior residents/fellows.^14^ Medical students are often frustrated by the fast-paced environment of modern inpatient medicine, where time pressures can prevent them from practicing more advanced clinical reasoning and decision making. In particular, medical students have limited chances to learn or practice handoffs, as work rounds are often cut short by the need for residents to honor duty hour restrictions and quickly give and receive clinical information.

This simulation exercise was designed to provide a safe environment for students and new residents to learn and practice patient handoffs. In order to provide a more robust learning experience, we developed a high-fidelity, standardized simulated patient and clinical scenario of a patient experiencing a spontaneous abortion.

Handoff simulations and curricula exist in the literature for pediatrics, surgery, emergency medicine, and adult general medicine and describe a variety of handoff techniques, including the I-PASS and ACCEPT mnemonics.^15–19^ Published curricula utilize video presentations, small-group learning through role-play, and didactic sessions.^15–20^ There is currently no published handoff curriculum specific to obstetrics and gynecology, a specialty with a unique vocabulary and elements of surgery and medicine and one that often deals with rapidly evolving clinical situations. Our curriculum fills this void by providing a specialty-specific simulation for practicing handoffs in obstetrics and gynecology that is adaptable to both medical students and residents. Additionally, our curriculum provides a unique, realistic immersion experience for learners in which they must interview a simulated patient, perform an examination, order tests and imaging, and synthesize clinical information through clinical reasoning. The scenario concludes with each learner performing a handoff based on the information the learner has gathered.

Prior to the simulation exercise, students are provided with two seminars offering an introduction to patient handoffs using the SBAR (situation, background, assessment, recommendations) technique, as well as instruction on the management of first-trimester bleeding. Spontaneous abortion with acute blood loss was chosen as the clinical scenario as it is a common occurrence that most physicians practicing in the fields of internal medicine, family practice, emergency medicine, obstetrics and gynecology, and surgery may confront during their careers. Additionally, the clinical skills required to manage acute blood loss are useful for all physicians to practice. The SBAR method was chosen for its simplicity, ease of use, and cross-disciplinary applications.^21^ The method has only four specific items, which can be expanded or compressed to fit different patients, and is thus readily adaptable to the unique components of an obstetric or gynecologic patient.

The curriculum consists of two didactic sessions, a simulation, and a debriefing. The curriculum begins with the two faculty-led interactive didactic sessions, which include background material for evaluation and management of a patient with first-trimester vaginal bleeding and an introduction to handoffs and patient safety. Learners then move on to the simulation, where they practice both patient management and handoffs. During the simulation, learners are observed and assessed by the faculty members. The simulation may be easily modified for either individual learners or small groups of learners. The simulation sessions are followed by a debriefing led by the faculty observers. The simulation sessions are intended to be immersive experiences to allow learners to safely practice formulating handoffs in a realistic environment, in addition to practicing information gathering and clinical reasoning.

## Methods

The exercise begins with two 45-minute didactic sessions, one on handoffs (Appendix A) and one on evaluation and management of first-trimester bleeding (Appendix B). Speaker notes are included for each session (Appendices E & F). Our results are based on didactic sessions in which the slides were presented in lecture format to students; we have included narrated versions of the slides (Appendices C & D) for use in a flipped classroom, for which the session slides may be assigned as preparatory work. Each simulation scenario should be allotted 15 minutes per student (10 to 12 minutes for the student to complete the simulation and 3 to 5 minutes for individual feedback). We allot 3 hours for a clerkship of six to eight students, including the didactic sessions and simulation. The simulation alone can be done in 1 hour per six to eight students as a group activity or 1 hour per four trainees as an individual activity. The students should be assigned reading or video reviews of first-trimester bleeding and spontaneous abortion prior to the session.^22,23^ The didactic session slides may also be assigned as preview material.

The role-play in the simulation can be run in two ways. In the first version, a learner gives a handoff to another learner. The participants enter into the scenario at different junctures, and each has the chance to receive a handoff, interview the patient, manage the clinical problem, and receive and interpret laboratory and imaging results as they are reported. The learner then gives a handoff to the next learner entering the scenario. This version allows learners to practice giving and receiving a handoff as well as managing the clinical problem for a short time. This version was used for our third-year clerkship students at Harvard Medical School.

In the second version, each learner completes the entire scenario of receiving a handoff, managing the patient, and concluding with giving a handoff. Learners are more completely immersed in the role and have more responsibility for the clinical care of the patient, but the scenario is more time consuming. This version was used for the fourth-year medical student obstetrics and gynecology boot camp course at Harvard Medical School.

The simulation can be adapted to a low-tech version if a simulation center intensive care unit is unavailable. In this version, file cards with the patient's clinical status can be given to the participating student as the scenario unfolds.

Further details are provided in the simulation guide (Appendix G) and role-play description (Appendix H).

### Equipment/Environment

The didactic sessions require a small-group learning space with audiovisual capacity for review of the two PowerPoint presentations. This space should also be adequate for the initial practice role-play of handoffs in small groups of two or three learners.

The simulation can be run in a simulation room for an emergency department, trauma bay, or triage area. The room requires a single bed with Laerdal monitors (or equivalent) for telemetry monitoring (blood pressure, heart rate, respiratory rate) and a Laerdal SimMan 3G (or equivalent) female mannequin.

Props and supplies needed include a blood pressure cuff monitor for the mannequin, intravenous fluids and tubing, simulated blood for bleeding, a 60-mL Luer-Lok syringe for simulated blood, disposable pads or chux for bed protection, a phone, and an observation window.

The faculty observer, the new-hire nurse (from the scenario), and the faculty monitor need communications headphones and earpieces.

Provide learners with the physical exam findings and laboratory results index cards (Appendix I) and the ultrasound report (Appendix J). Learners should have a means of recording information (paper, whiteboard, or electronic) for history taking and to facilitate their handoff. It is not expected that the learner will generate a written handoff.

There are no distractors for this resource.

### Personnel

Simulation personnel needed include a voice actor for the patient mannequin, an actor for the role of the new-hire nurse, and an actor for the attending. In our experience, we have used our clerkship staff for the voice of the patient, simulation center nurses for the role of the new-hire nurse, and residents or physician faculty for the role of the attending. Although using actors with medical knowledge is helpful, these roles could be scripted and played by actors without medical or nursing training. A third staff member may be helpful if using programmable telemetry monitors, to operate the technical equipment; in our experience, the simulation center nurse can adjust the telemetry equipment as well as play the role of the new-hire nurse.

Faculty needed for the curriculum include a facilitator for the two didactic sessions and two observers for the simulation. One faculty member can serve a dual role as the attending in the simulation and one of the observers. A single faculty member may staff the curriculum, but this decreases the accuracy of observations. More faculty observers could also be utilized. All observers should participate in the debriefing. Faculty may consist of upper-level residents, fellows, or attending physicians. Having more-experienced physicians involved is particularly valuable for the debriefing sessions.

### Assessment

Faculty observers use the student assessment tool (Appendix K) to evaluate learners. The assessment tool was developed to correspond to our institution's grading metrics and as such includes some categories that are not directly mapped to the EPAs of the AAMC. The assessment tool does incorporate eight of the EPAs and also has categories for professionalism and interpersonal skills. The assessment tool uses a 5-point Likert scale anchored at *appropriate to level of training* and can thus be adjusted to different levels of learners. The assessment tool also includes a comments section for observers to take notes of particular areas of concern or exemplary behavior. Although a single observer can be used, we have found two observers to be appropriate as the simulation can be fast paced and one observer may miss some aspects of the learner's performance.

### Debriefing

Faculty observers use the debrief checklist (Appendix L) to guide the conversation. Appendix L includes prompt questions, an outline of the debriefing structure, and two checklists, one to track the facilitator's review and one to track the learner's responses to the debrief.

The debrief can occur in a small group if the learners have participated in the simulation in small groups or individually for learners who complete the entire simulation alone. All observers should participate in the debrief for any simulation session they observe. The debrief should occur immediately after the simulation session and can take place in the simulation room or a separate space.

Faculty observers should first check in emotionally with the learners. “So, how did that feel?” is a useful icebreaker. In our experience, most trainees are anxious and need a few minutes to process the experience. Reassurance regarding learners’ management decisions is important, as is a reminder that simulations are an opportunity to safely practice skills and that a learner's performance is not used in summative evaluations (we have used this simulation exercise only in formative assessments).

Debriefing should include asking learners whether they used the SBAR technique in receiving or giving handoffs; if not, ask how SBAR could have helped the handoff. Ask the learners if they used closed-loop communication and if they remembered to ask questions and clarify the handoff they received.

Debriefing should include a review of clinical findings found within the simulation and an explanation of the significance of each item. A review of these findings and their significance is included in the debrief checklist.

Debriefing should also include an opportunity for the learner to make comments or suggestions for improvement and to discuss any areas of concern the learner may have regarding his or her own performance. The final checklist in Appendix L provides space for these comments. The learners’ suggestions and feedback can be used by clerkship or program directors to continue to refine and improve the curriculum.

## Results

Between 2013 and 2016, 46 third-year and 22 fourth-year medical students participated in the simulation curriculum. Students were surveyed at the conclusion of the simulation. The survey included four questions and utilized a 5-point Likert scale where 1 = *strongly disagree* and 5 = *strongly agree*. Students found the exercise useful. Ninety-five percent agreed the simulation was relevant to the third-year clinical experience, 90% agreed the simulation taught handoff skills, 80% agreed the exercise was realistic, and 73% agreed the simulation increased confidence in handoffs.

Student comments for the simulation have been consistently positive:

• “Appropriate case for clerkship level, good experience in working up a patient, loved it.”
• “The exercise contributed to my understanding of sign out rounds.”
• “Gave us a chance to practice making decisions in stressful situations.”
• “The simulation felt real.”

In response to student feedback, we incorporated a group debriefing session as well as individual feedback after the simulation. Students suggested the exercise occur toward the end of the clerkship; however, scheduling logistics did not always allow for this timing. We did not collect data to evaluate the effect of scheduling within the clerkship in regard to student performance or satisfaction scores. Student feedback has suggested more time be allotted for history taking during the simulation; however, we feel that a defined time frame contributes to the realism of the exercise and has educational benefit.

Our assessment tool for the exercise was based on the elements of the clerkship grading scheme currently used for students at our institution. Areas include history taking, fund of knowledge, evaluation and management, interpersonal skills, presentation skills, professionalism, desire to learn, and systems awareness. We used a Likert scale where 1 = *below expectations* and 5 = *consistently above expectations*. Two faculty observed and evaluated each student. We considered interrater reliability strong if the *r* value was more than .70, moderate if between .60 and .69, fair if between .40 and .59, and weak if less than .40. The skills assessment area in which there was the highest interrater agreement was for fund of knowledge (*r* = .66); the area of least agreement was evaluation and management (*r* = .34). There was moderate correlation between observers on assessment scores in fund of knowledge (*r* = .66) and fair correlation on assessment scores of interpersonal skills (*r* = .58), history taking (*r* = .47), systems awareness (*r* = .44), desire to learn (*r* = .42), and professionalism (*r* = .41). There was weak correlation between observers for assessment of presentation skills (*r* = .38) and evaluation and management (*r* = .34).

## Discussion

Our curriculum provides an opportunity for the learning and practice of patient handoffs that students found relevant and realistic. The simulation exercise was effective and valued by students, who frequently noted this immersive and realistic simulation experience was a unique opportunity to practice patient management skills and handoffs. The curriculum may be limited in scale due to time constraints, as well as the space requirements for a simulation center with mannequin and staff to run the simulation. The curriculum may be adaptable to use without a simulation laboratory using role-play, though a low-tech version of the exercise might lessen the immersive experience of directly managing a patient's clinical status, and student satisfaction could be decreased.

We have used this curriculum in both a third-year obstetrics and gynecology clerkship and a fourth-year residency preparation boot camp course. In both settings, we used a formative, low-stakes approach, which was explicitly stated to the students at the start of the simulation session. In our experience, the clerkship students benefit from the group activity. Structuring the simulation as a group activity provides the opportunity for both faculty and peer direct observation and feedback. In the setting of a residency preparation course, students benefit from the individual activity as a more realistic simulation of patient management and handoff skills. This simulation was developed as a formative experience, and the assessment tool was developed as such. The simulation may be adapted to a higher-stakes evaluation for promotion or competency assessment; however, this was not our intent.

Our assessment tool was based on a clerkship grading tool currently used for students at our institution. We found moderate interrater reliability for observers of the simulation only in the area of fund of knowledge. We employed three faculty members during the academic year to staff the simulation; with a more diverse group of observers, the interrater reliability may improve. In our experience, a team of two to three faculty observers worked well. The simulation is complex, and at times, it is hard for observers to evaluate all aspects of a trainee's performance. Interrater reliability may be improved by assigning each observer to focus on a limited set of metrics rather than a global approach. However, using a single observer does not allow adequate assessment of both clinical decision-making and handoff skills. In training observers, faculty may benefit from a short review of SBAR techniques and observation of experienced observers. The assessment tool may provide objective assessment of clinical performance on fund of knowledge, but it has not been shown to predict clinical performance in other areas. The assessment tool may be improved by focusing only on handoff skills and not on patient evaluation and management.

## Appendices

A. Patient Handoffs in Obstetrics and Gynecology.pptx
B. Approach to Diagnosis and Management of First Trimester Bleeding.pptx
C. Patient Handoffs in Obstetrics and Gynecology Narrated.mp4
D. Approach to Diagnosis and Management of First Trimester Bleeding Narrated.mp4
E. Handoff Skills Speakers Notes.docx
F. First Trimester Bleeding Speakers Notes.docx
G. Simulation Guide.docx
H. Role Play Description.docx
I. Trainee Simulation Information Cards.doc
J. Ultrasound Report.docx
K. Student Assessment Tool.docx
L. Debrief Checklists.docx All appendices are peer reviewed as integral parts of the Original Publication.
