# Supplementary material for: Teaching Patient Handoffs to Medical Students in Obstetrics and Gynecology: Simulation Curriculum and Assessment Tool
Source: MedEdPORTAL. 2016 Oct 2;12:10479. doi: 10.15766/mep_2374-8265.10479 (PMC6440488; doi:10.15766/mep_2374-8265.10479)
Supplement: Supplementary file 1 — A. Patient Handoffs in Obstetrics and Gynecology.pptx B. Approach to Diagnosis and Management of First Trimester Bleeding.pptx C. Patient Handoffs in Obstetrics and Gynecology Narrated.mp4 D. Approach to Diagnosis and Management of First Trimester Bleeding Narrated.mp4 E. Handoff Skills Speakers Notes.docx F. First Trimester Bleeding Speakers Notes.docx G. Simulation Guide.docx H. Role Play Description.docx I. Trainee Simulation Information Cards.doc J. Ultrasound Report.docx K. Student Assessment Tool.docx L. Debrief Checklists.docx [file mep-12-10479-s001.zip › L. Debrief Checklists.docx]

**Teaching Patient Handoffs to Medical Students in Obstetrics and Gynecology: A Simulation Curriculum and Assessment Tool**

**Appendix L: Debrief Checklist**

1. Rules for the debriefing:
   - - - 1. Check in with trainees emotionally.
         2. Reassure students regarding management decisions.
         3. Emphasize this is an opportunity to practice handoff and clinical management skills.
         4. Emphasize this is not used in assessment or evaluation.
2. Questions to facilitate the debriefing:
   - - 1. When you gave the handoff, did you use the SBAR technique? If not, why not? Would SBAR have helped you to be more complete in your handoff?
       2. When you received the handoff did you remember to ask questions and clarify? If not, why not?
       3. Was closed-loop communication used in the handoffs?
       4. What could have gone better?
       5. How would you improve the scenario?
       6. Review the clinical findings and explain each significant item:
          1. Pre-existing anemia contributes to patient's rapid decline.
          2. Enlarged uterus is due to fibroids, but may be due to multiple gestations, molar pregnancy.
          3. The trainees may have been distracted be the social history, suggestive of ongoing domestic violence issues, or the possibility of recurrent pregnancy loss. The trainees should recognize the need for emergency surgery and not be distracted by non-urgent findings.
          4. Some findings that may seem incidental are significant, for example the Rh-negative status indicates need for rhogam.
          5. Pelvic exam findings with open cervical os and tissue protruding indicate incomplete spontaneous abortion and need for surgical management.
          6. Rapid decompensating indicates need for surgical management.

Checklist 1: Facilitator Review

|  |  |
| --- | --- |
| Emotional Response to Scenario Addressed (Facilitator) |  |
| Reassurance on performance (Facilitator) |  |
| Reassurance not used in Summative Assessment (Facilitator) |  |
| Pre-existing anemia contributes to patient's rapid decline. |  |
| Enlarged uterus is due to fibroids, but may be due to multiple gestations, molar pregnancy. |  |
| The trainees may have been distracted be the social history, suggestive of ongoing domestic violence issues, or the possibility of recurrent pregnancy loss. The trainees should recognize the need for emergency surgery and not be distracted by non-urgent findings. |  |
| Some findings, which may seem incidental are significant, for example the Rh-negative status indicates need for rhogam. |  |
| Pelvic exam findings with open cervical os and tissue protruding indicate need for surgical management. |  |
| Rapid decompensating indicates need for surgical management. |  |

Checklist 2: Trainee’s Performance and Response

| Desired Behavior | Trainee Comment | Facilitator 1 Comment | Facilitator 2 Comment |
| --- | --- | --- | --- |
| Emotional Response to Scenario (Facilitator) |  |  |  |
| Reassurance on performance (Facilitator) |  |  |  |
| Reassurance not used in Summative Assessment (Facilitator) |  |  |  |
| SBAR used? If not, why not? |  |  |  |
| Recipient given chance to ask questions? |  |  |  |
| Closed-loop Communication used? |  |  |  |
| What could have gone better? |  |  |  |
| How can we improve? |  |  |  |
