# Supplementary material for: Teaching Patient Handoffs to Medical Students in Obstetrics and Gynecology: Simulation Curriculum and Assessment Tool
Source: MedEdPORTAL. 2016 Oct 2;12:10479. doi: 10.15766/mep_2374-8265.10479 (PMC6440488; doi:10.15766/mep_2374-8265.10479)
Supplement: Supplementary file 1 — A. Patient Handoffs in Obstetrics and Gynecology.pptx B. Approach to Diagnosis and Management of First Trimester Bleeding.pptx C. Patient Handoffs in Obstetrics and Gynecology Narrated.mp4 D. Approach to Diagnosis and Management of First Trimester Bleeding Narrated.mp4 E. Handoff Skills Speakers Notes.docx F. First Trimester Bleeding Speakers Notes.docx G. Simulation Guide.docx H. Role Play Description.docx I. Trainee Simulation Information Cards.doc J. Ultrasound Report.docx K. Student Assessment Tool.docx L. Debrief Checklists.docx [file mep-12-10479-s001.zip › A. Patient Handoffs in Obstetrics and Gynecology.pptx]

## Slide 1
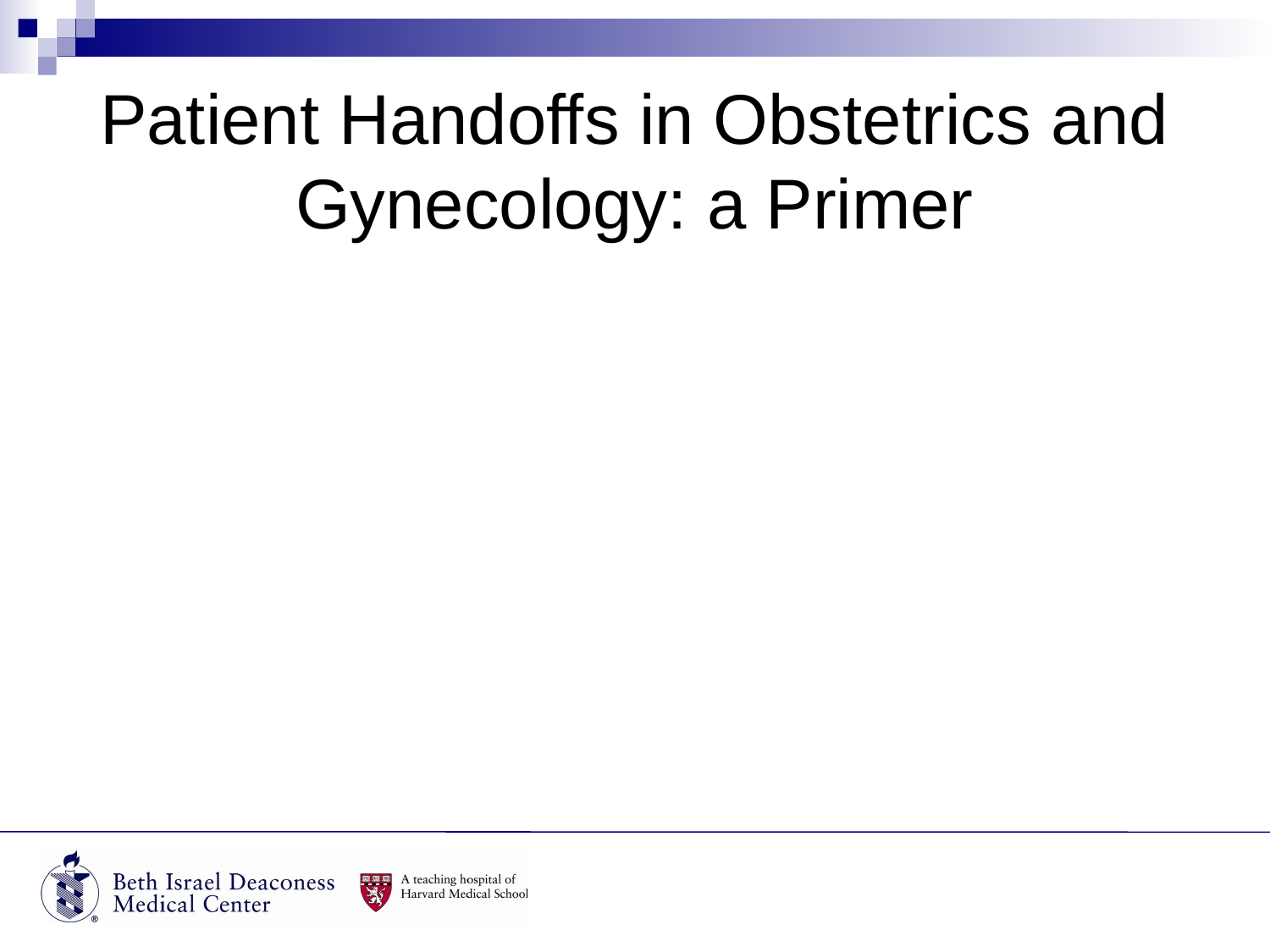

# Patient Handoffs in Obstetrics and Gynecology: a Primer

## Slide 2
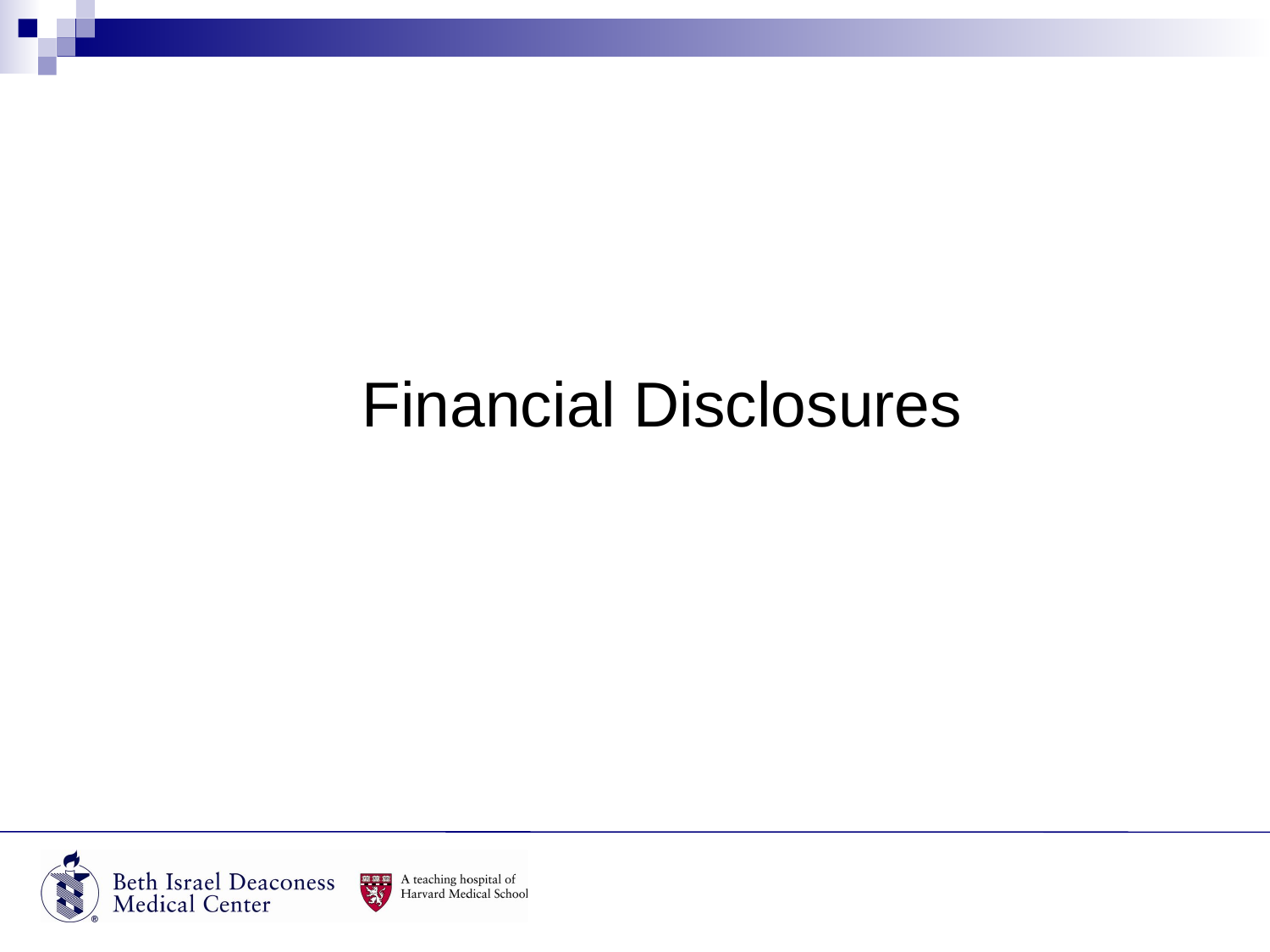

Financial Disclosures

## Slide 3
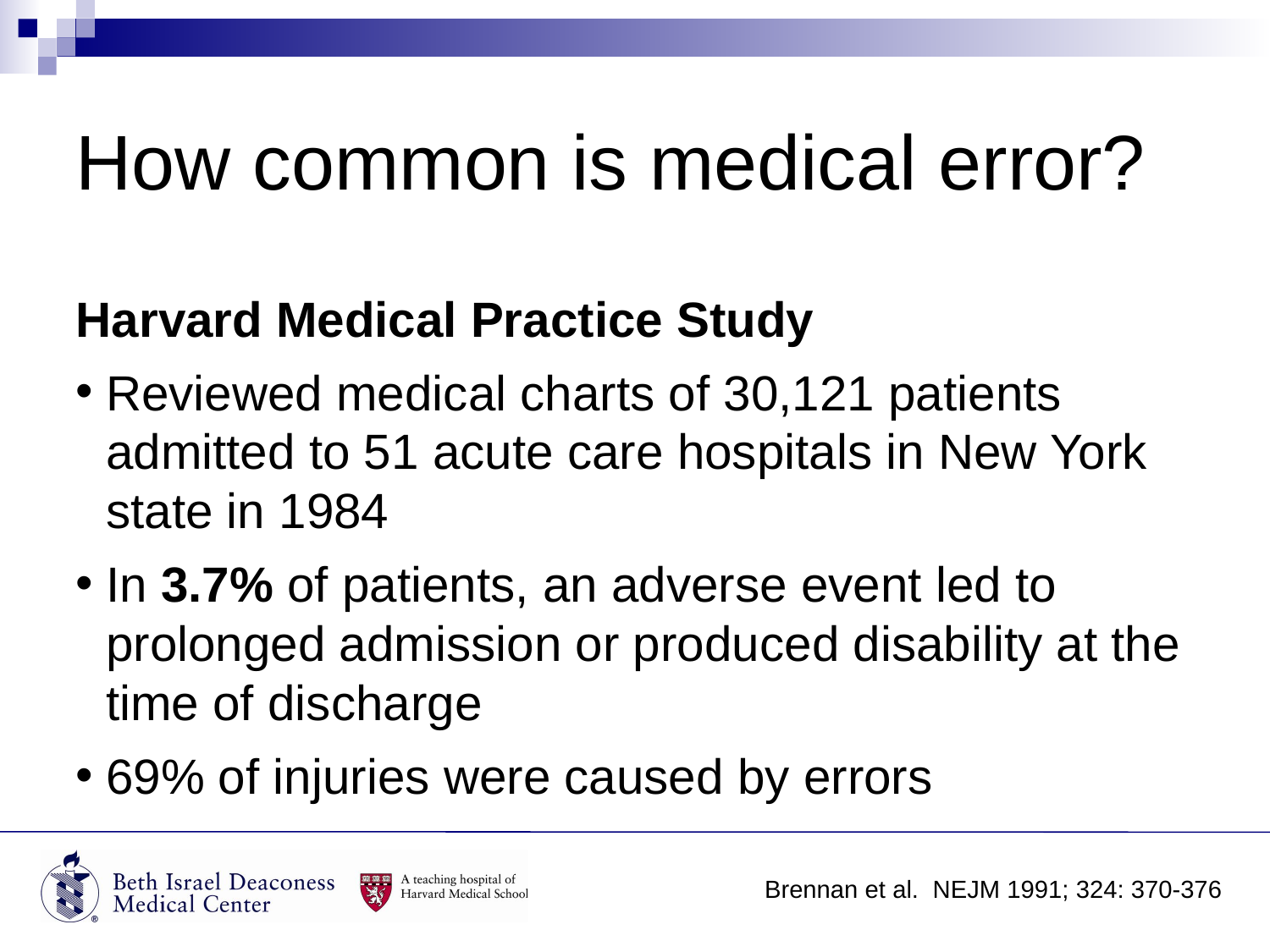

How common is medical error?
Harvard Medical Practice Study
Reviewed medical charts of 30,121 patients admitted to 51 acute care hospitals in New York state in 1984
In 3.7% of patients, an adverse event led to prolonged admission or produced disability at the time of discharge
69% of injuries were caused by errors
Brennan et al. NEJM 1991; 324: 370-376

## Slide 4
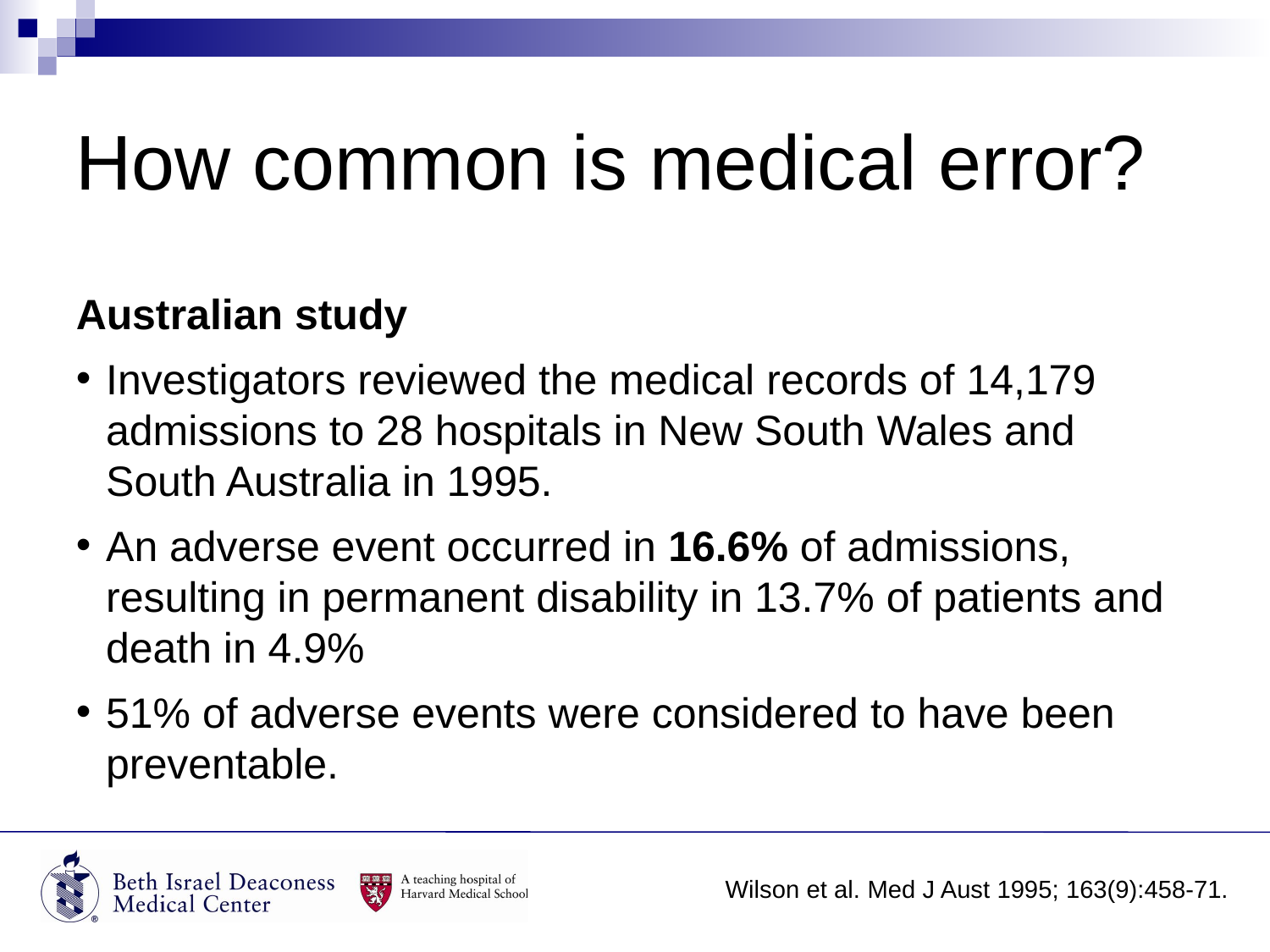

How common is medical error?
Australian study
Investigators reviewed the medical records of 14,179 admissions to 28 hospitals in New South Wales and South Australia in 1995.
An adverse event occurred in 16.6% of admissions, resulting in permanent disability in 13.7% of patients and death in 4.9%
51% of adverse events were considered to have been preventable.
Wilson et al. Med J Aust 1995; 163(9):458-71.

## Slide 5
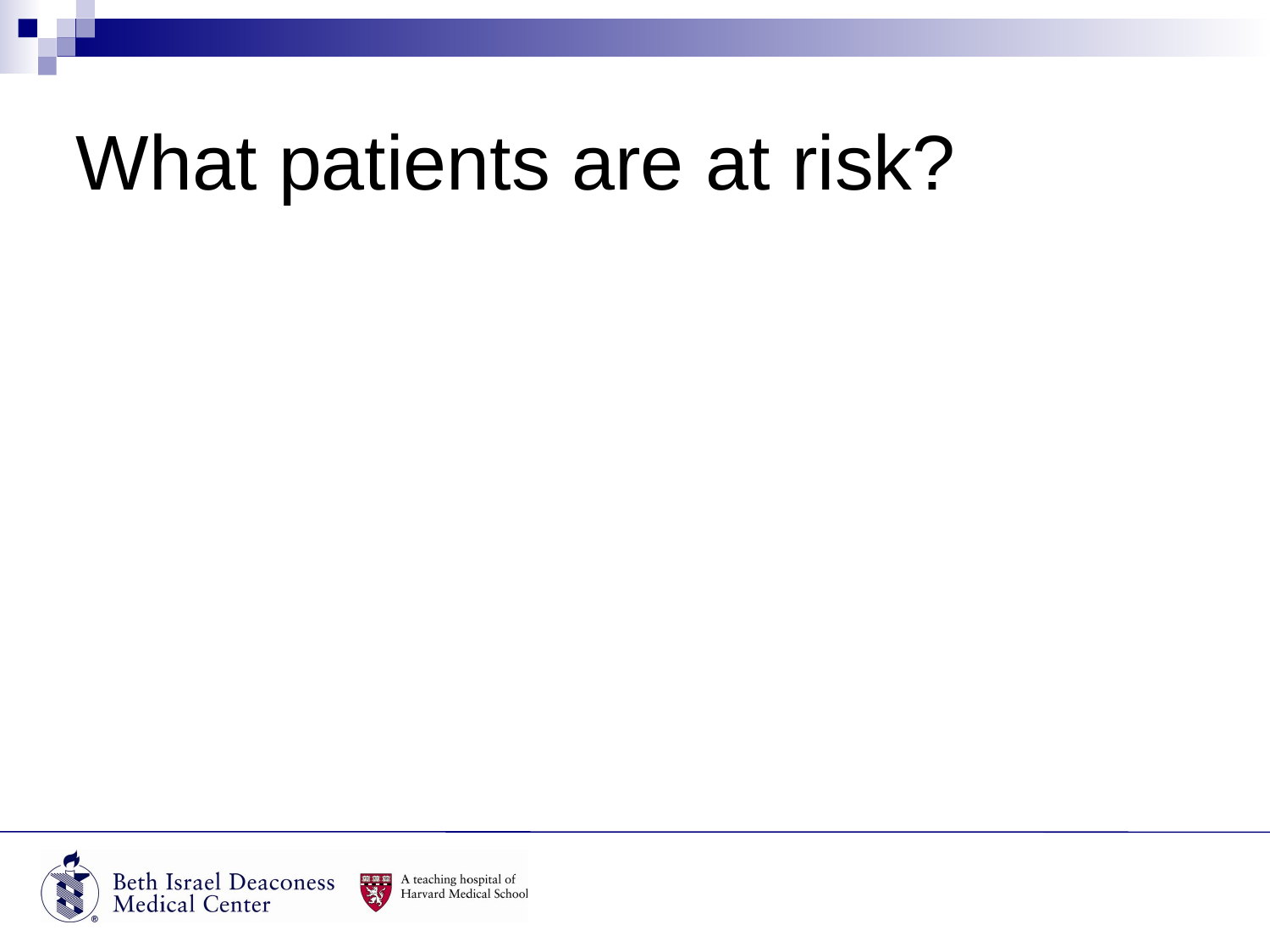

What patients are at risk?

## Slide 6
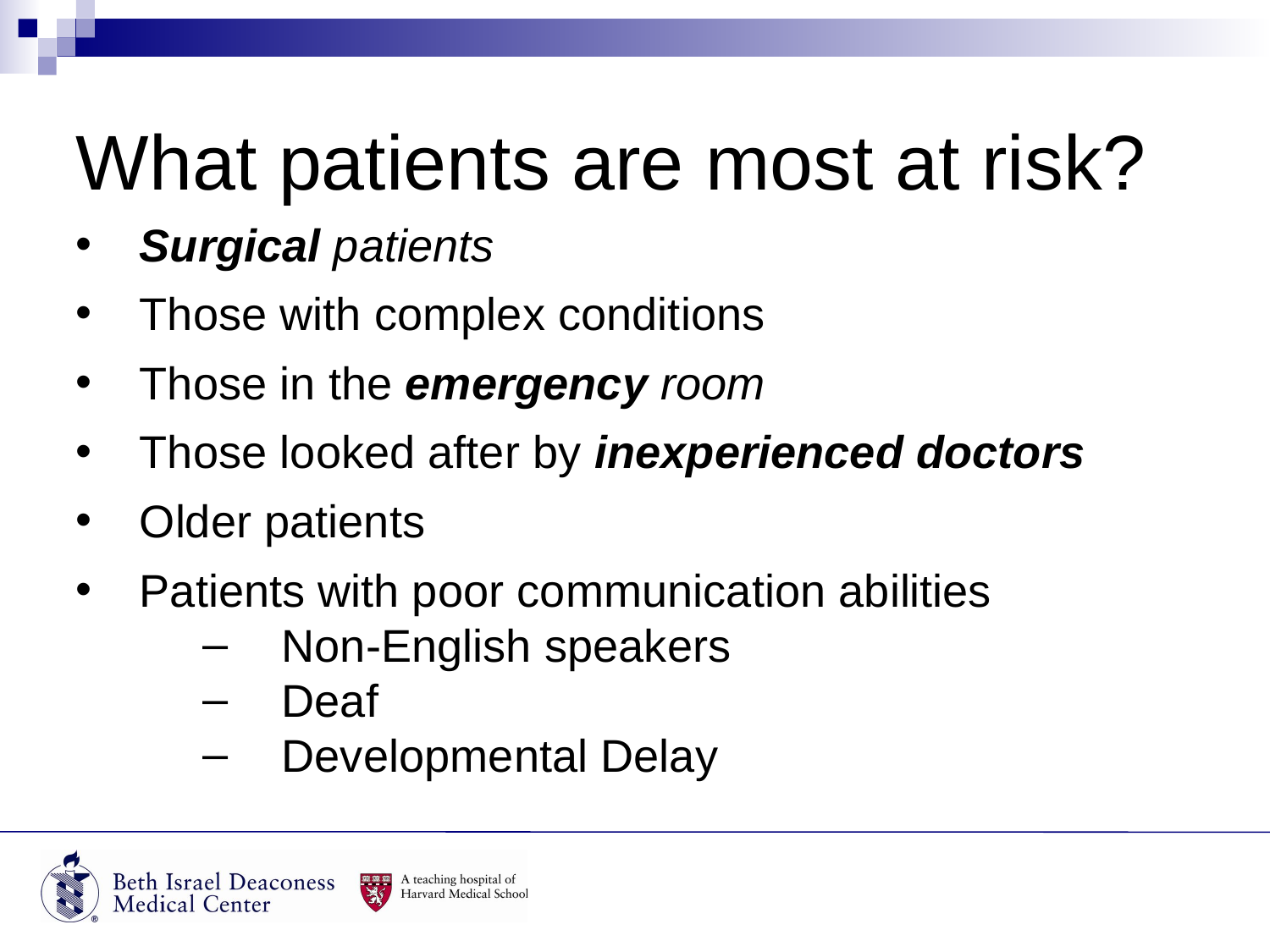

What patients are most at risk?
Surgical patients
Those with complex conditions
Those in the emergency room
Those looked after by inexperienced doctors
Older patients
Patients with poor communication abilities
Non-English speakers
Deaf
Developmental Delay

## Slide 7
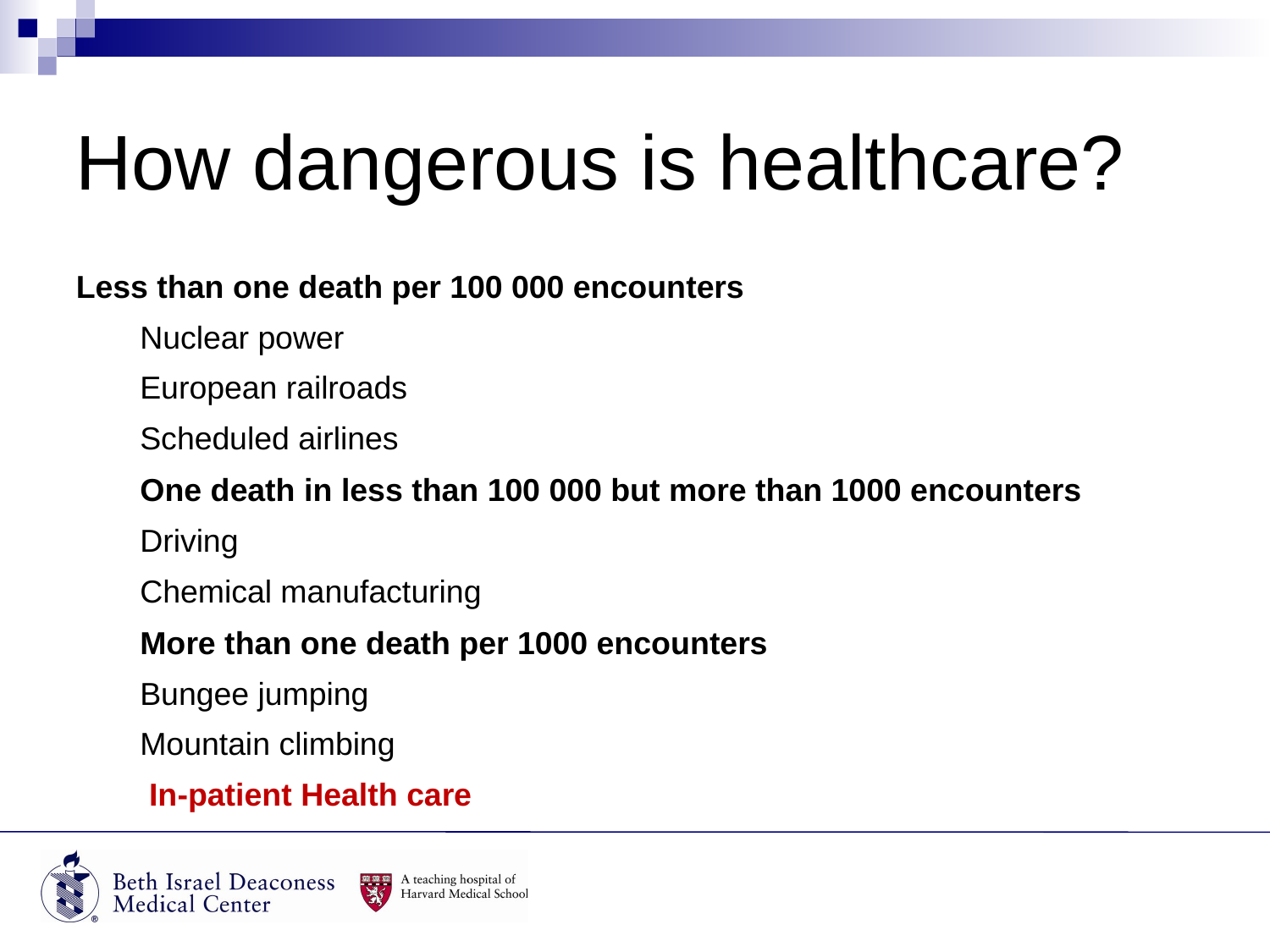

How dangerous is healthcare?
Less than one death per 100 000 encounters
Nuclear power
European railroads
Scheduled airlines
One death in less than 100 000 but more than 1000 encounters
Driving
Chemical manufacturing
More than one death per 1000 encounters
Bungee jumping
Mountain climbing
 In-patient Health care

## Slide 8
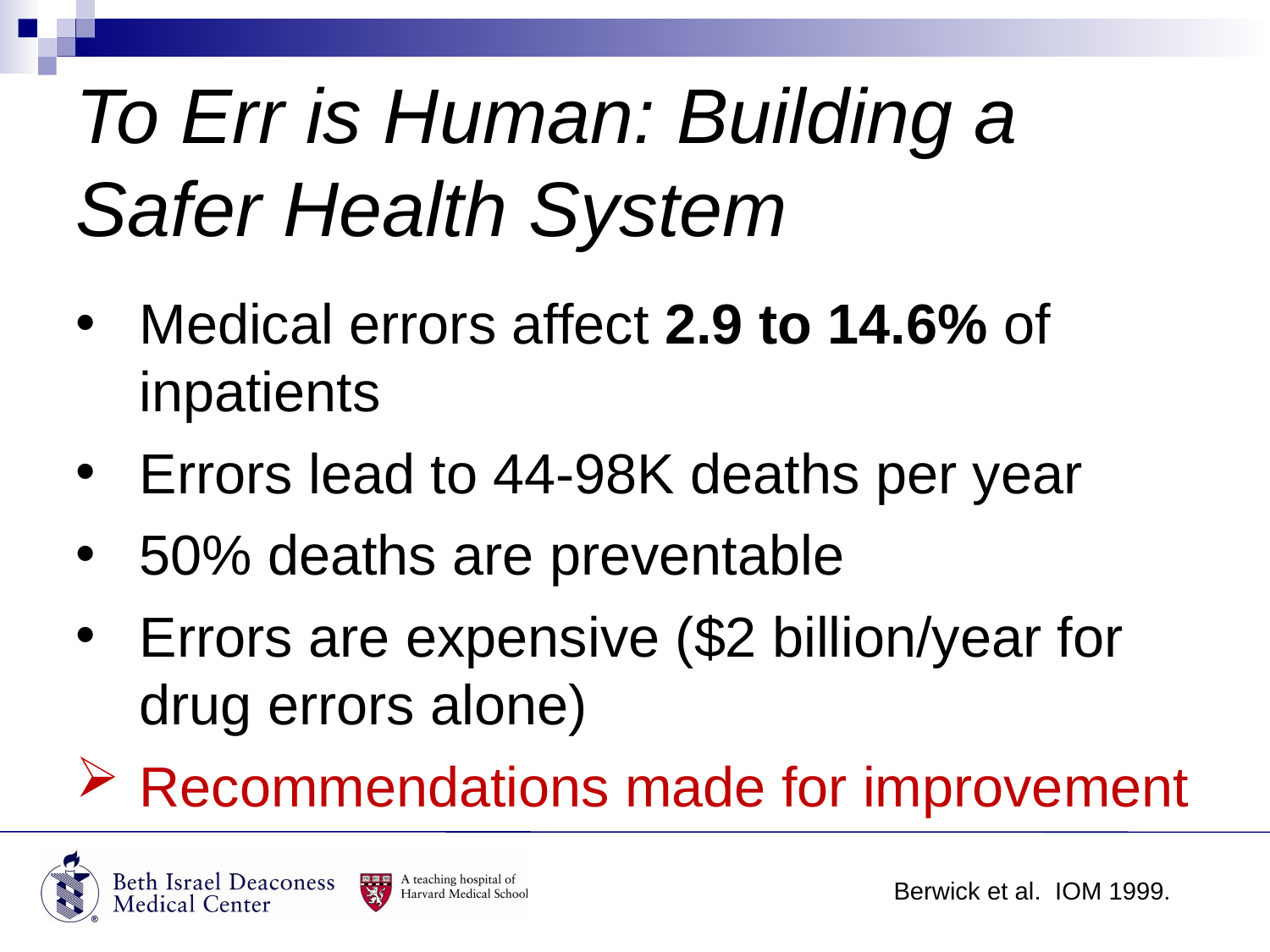

To Err is Human: Building a Safer Health System
Medical errors affect 2.9 to 14.6% of inpatients
Errors lead to 44-98K deaths per year
50% deaths are preventable
Errors are expensive ($2 billion/year for drug errors alone)
Recommendations made for improvement
Berwick et al. IOM 1999.

## Slide 9
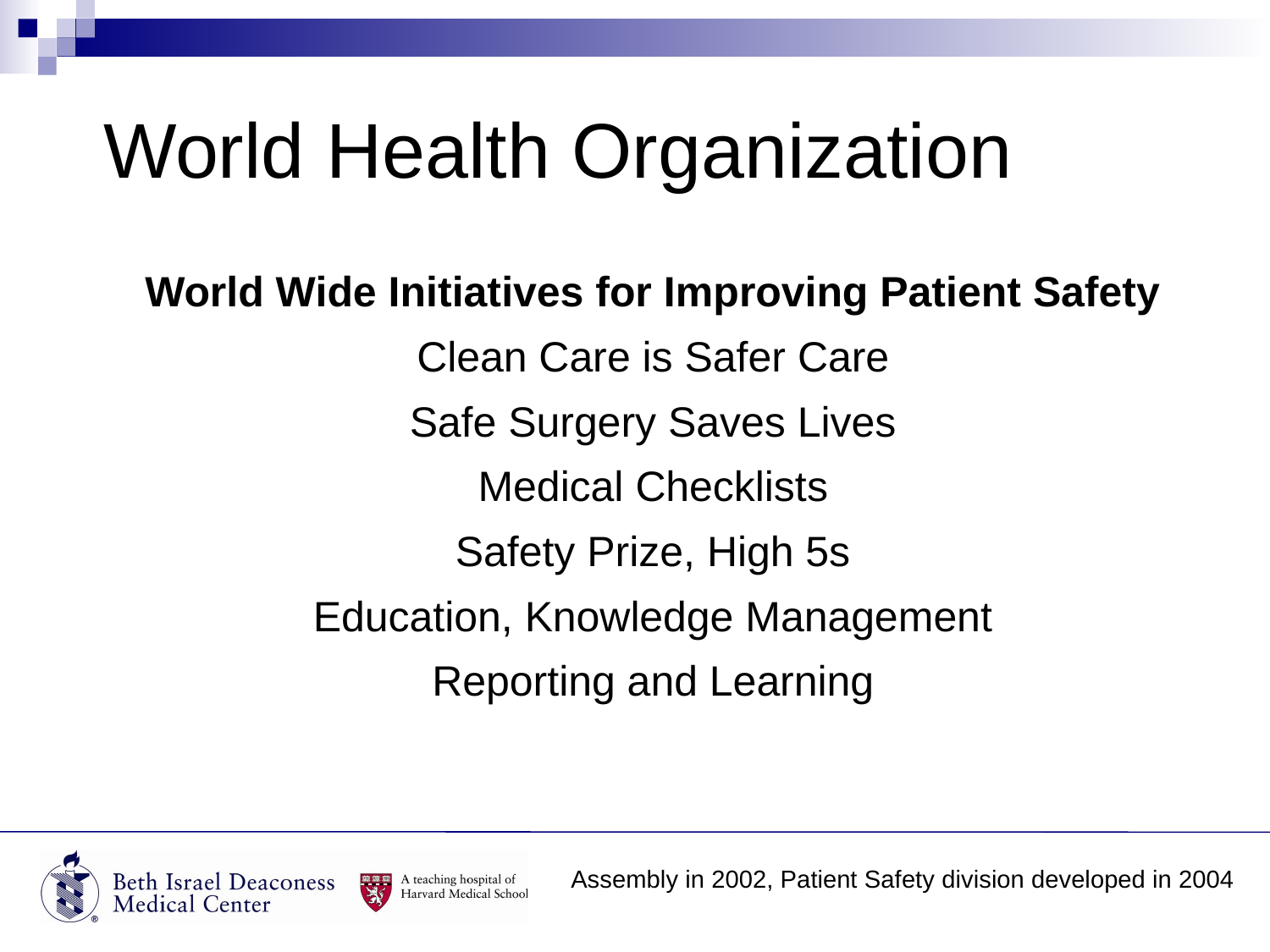

# World Health Organization
World Wide Initiatives for Improving Patient Safety
Clean Care is Safer Care
Safe Surgery Saves Lives
Medical Checklists
Safety Prize, High 5s
Education, Knowledge Management
Reporting and Learning
Assembly in 2002, Patient Safety division developed in 2004

## Slide 10
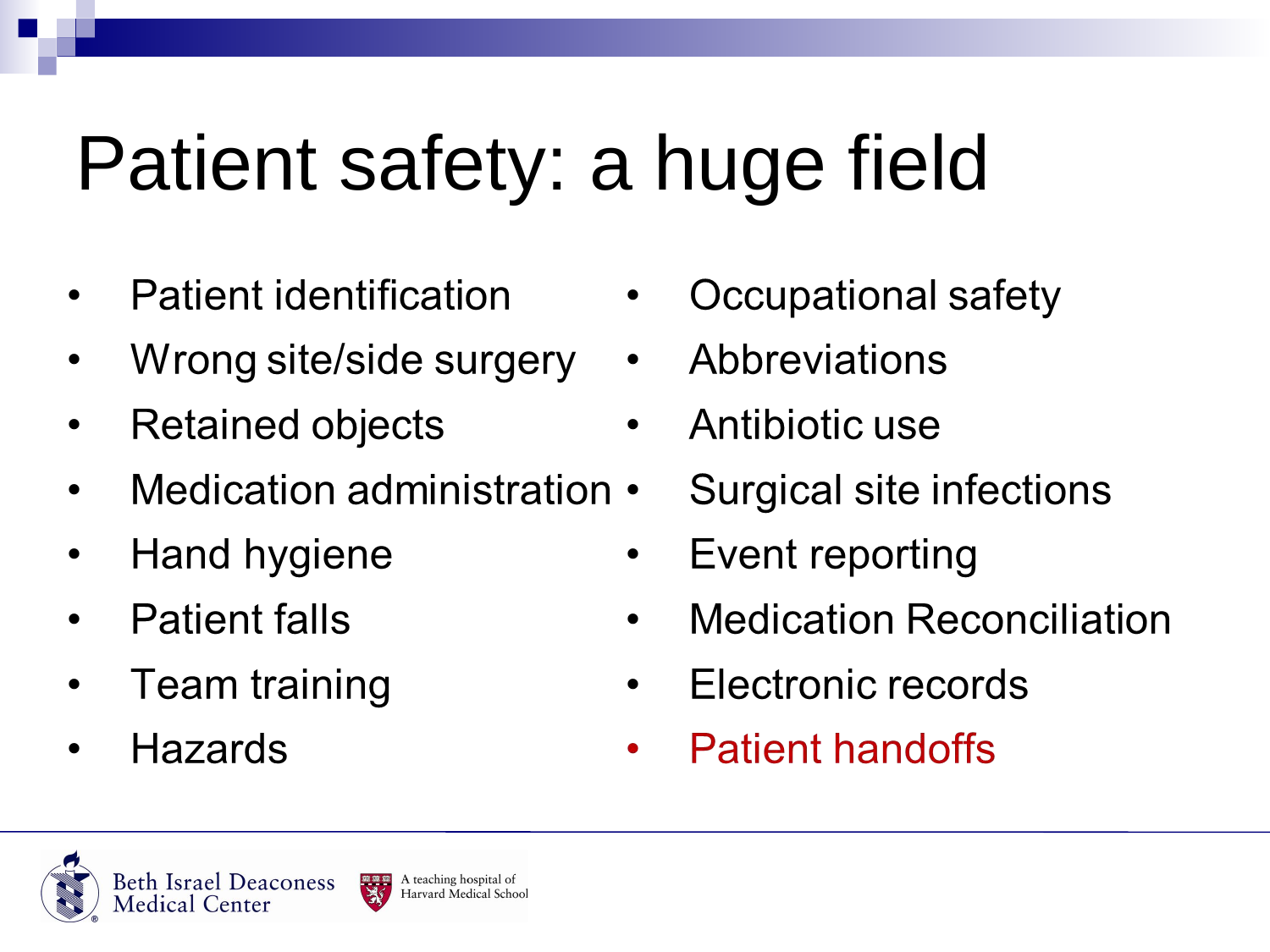

Patient safety: a huge field

## Slide 11
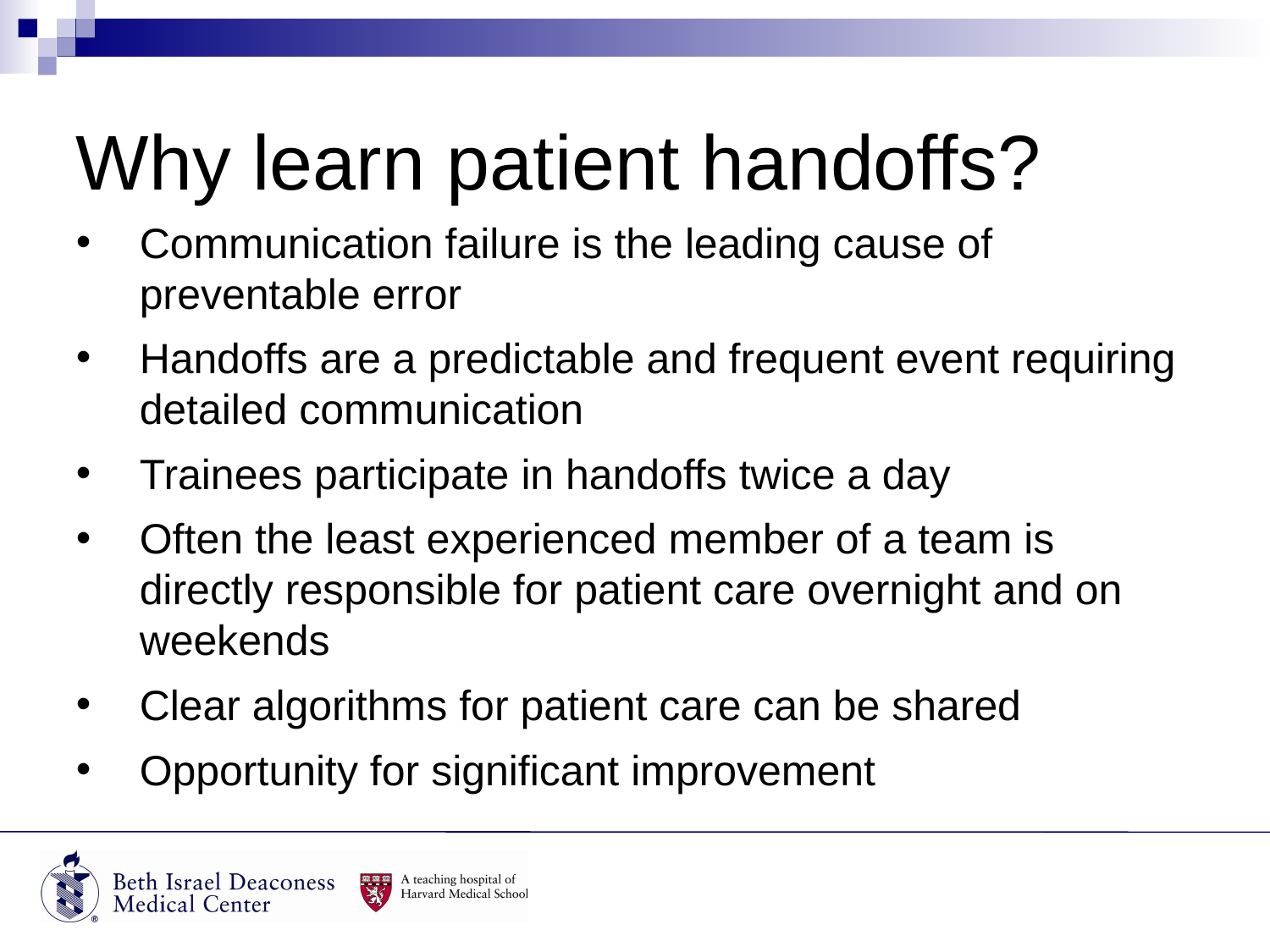

Why learn patient handoffs?
Communication failure is the leading cause of preventable error
Handoffs are a predictable and frequent event requiring detailed communication
Trainees participate in handoffs twice a day
Often the least experienced member of a team is directly responsible for patient care overnight and on weekends
Clear algorithms for patient care can be shared
Opportunity for significant improvement

## Slide 12
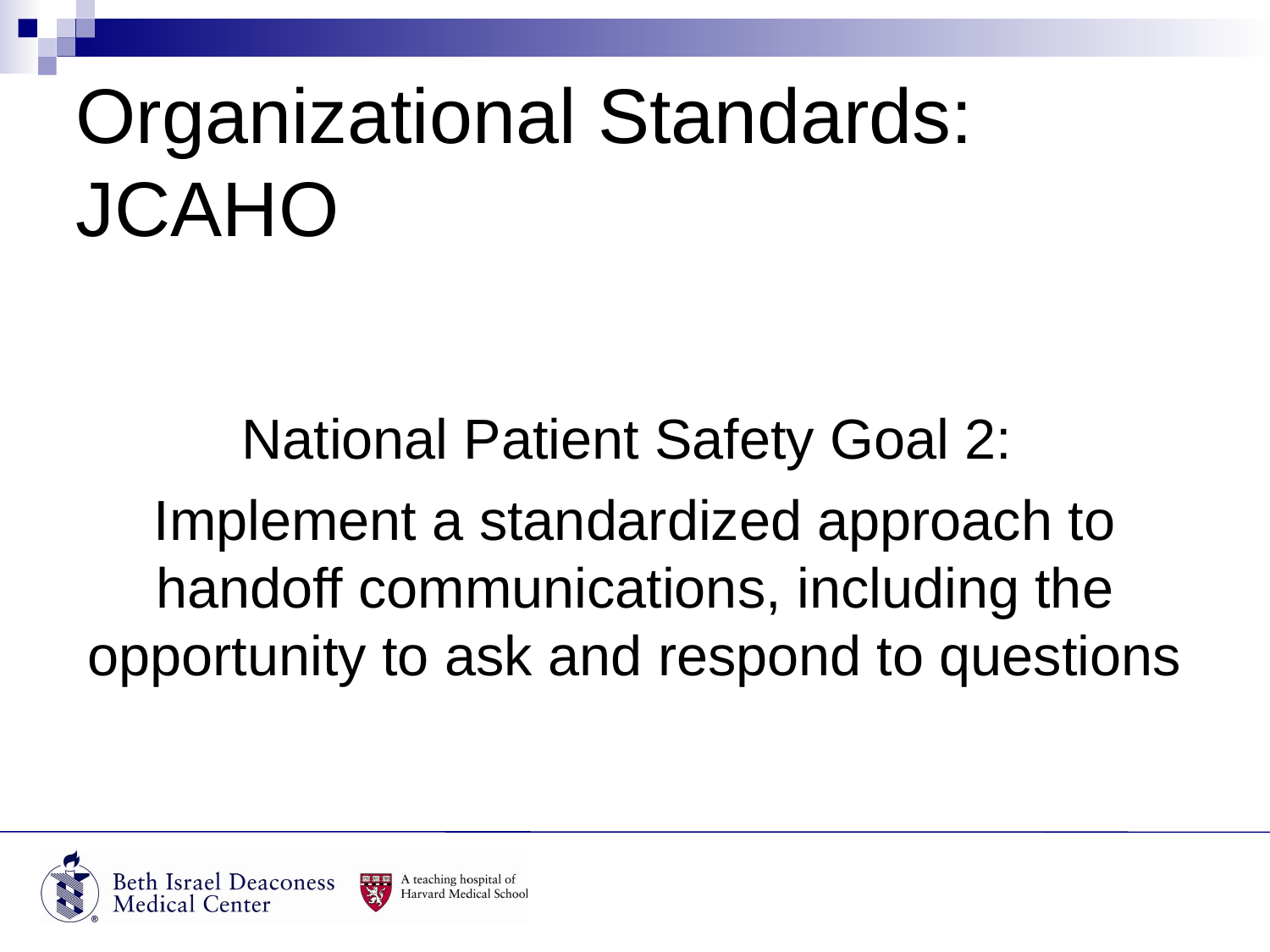

Organizational Standards: JCAHO
National Patient Safety Goal 2:
Implement a standardized approach to handoff communications, including the opportunity to ask and respond to questions

## Slide 13
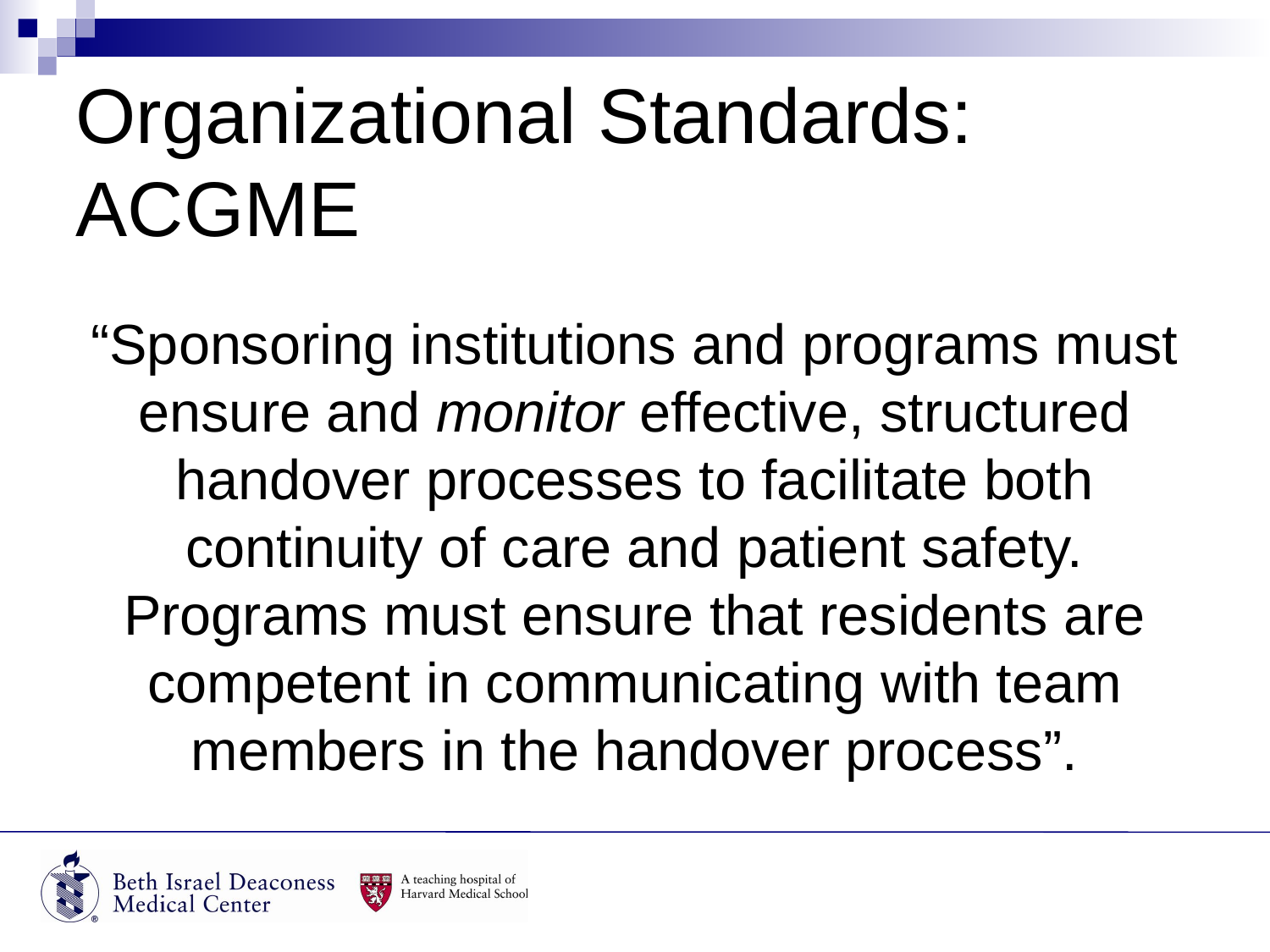

Organizational Standards: ACGME
“Sponsoring institutions and programs must ensure and monitor effective, structured handover processes to facilitate both continuity of care and patient safety. Programs must ensure that residents are competent in communicating with team members in the handover process”.

## Slide 14
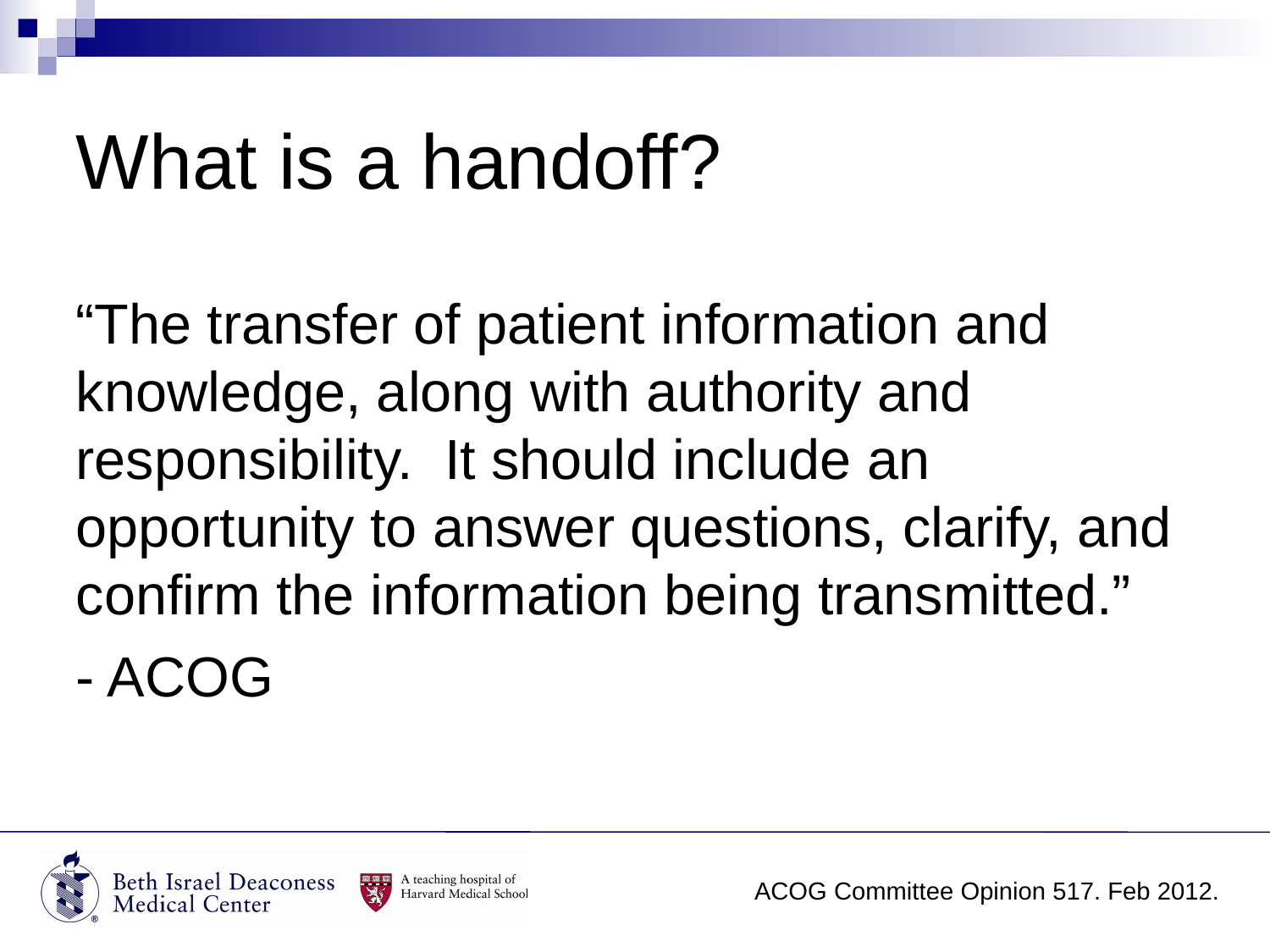

What is a handoff?
“The transfer of patient information and knowledge, along with authority and responsibility. It should include an opportunity to answer questions, clarify, and confirm the information being transmitted.”
- ACOG
ACOG Committee Opinion 517. Feb 2012.

## Slide 15
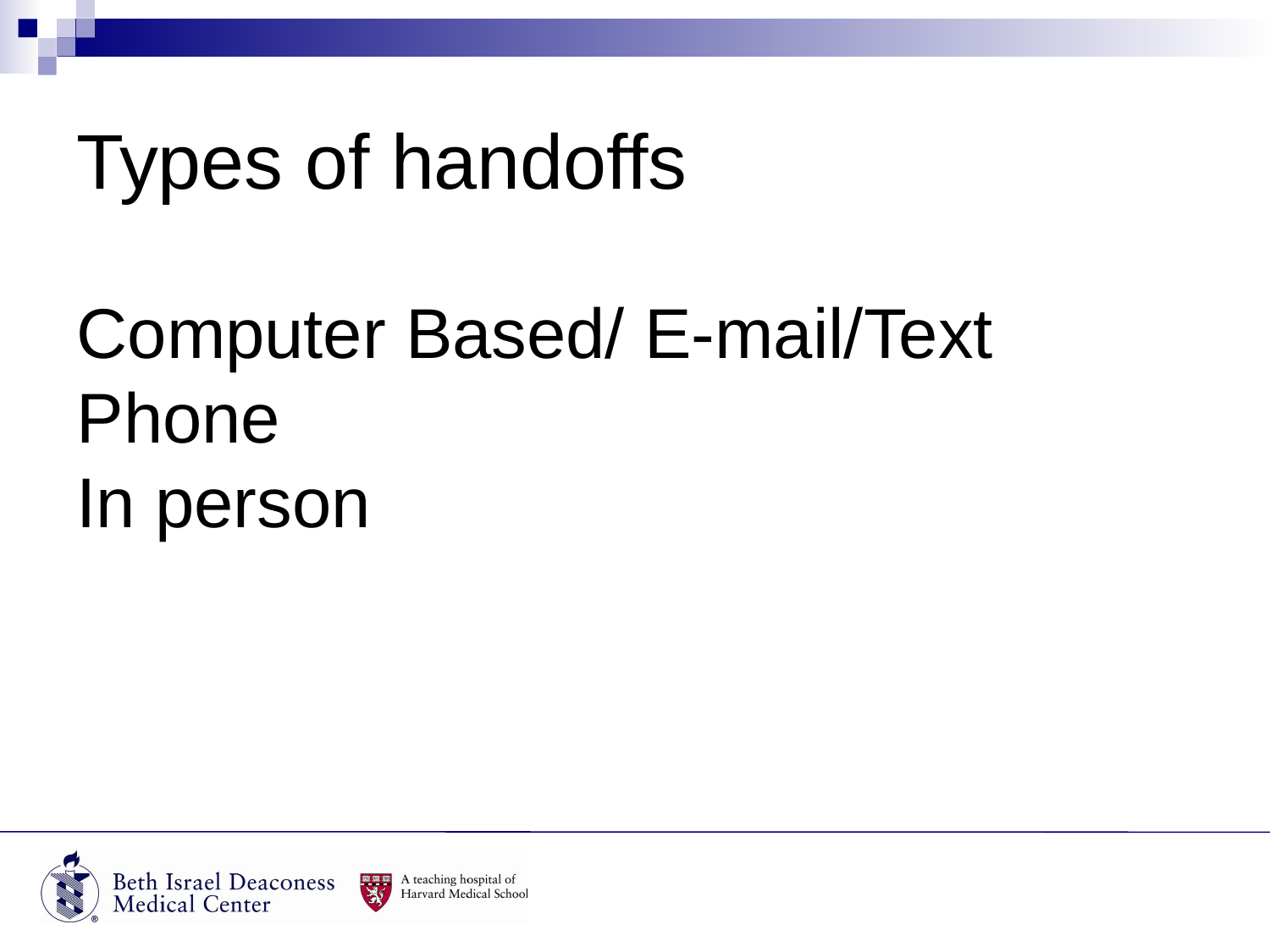

# Types of handoffs
Computer Based/ E-mail/Text
Phone
In person

## Slide 16
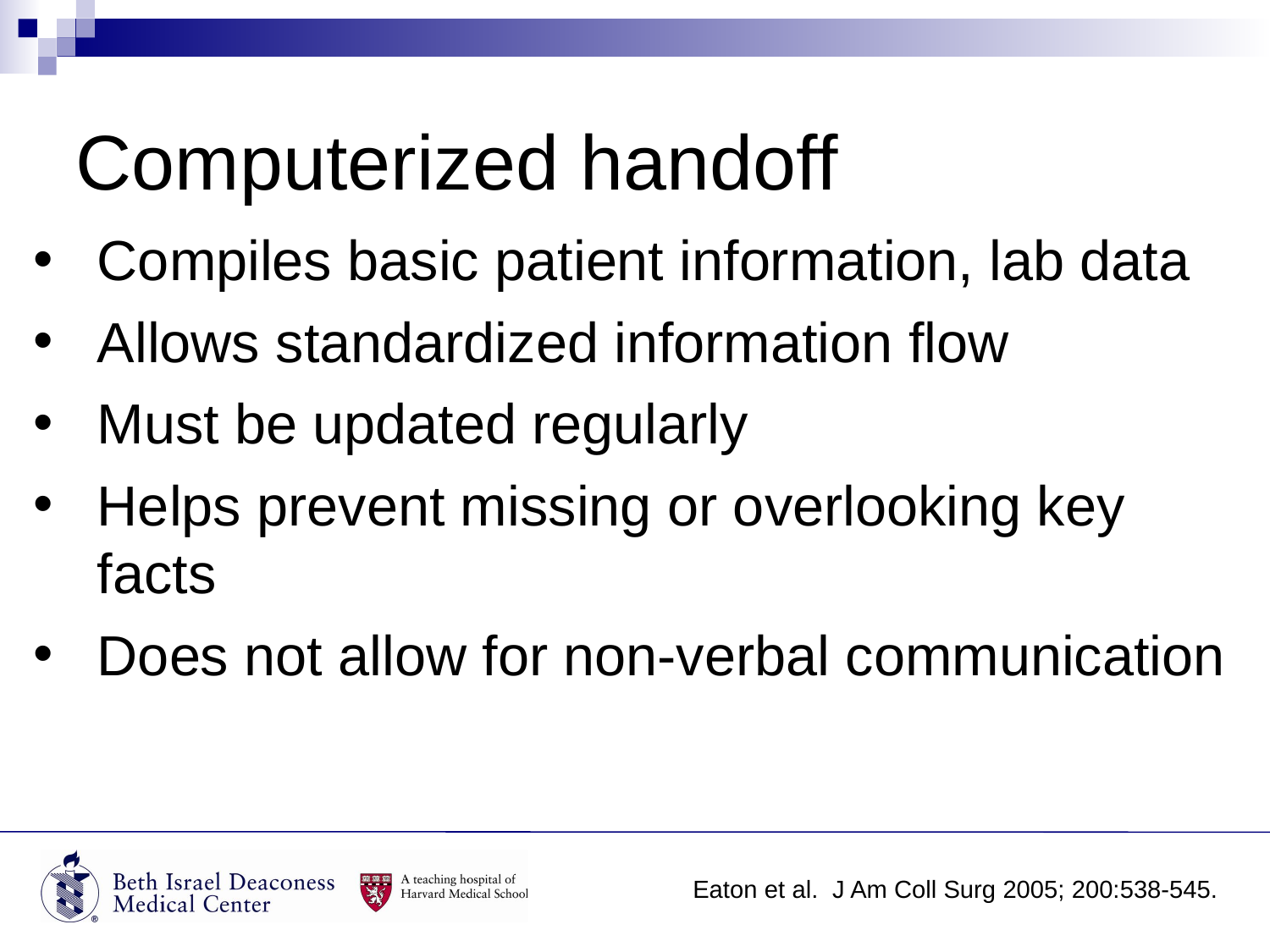

Computerized handoff
Compiles basic patient information, lab data
Allows standardized information flow
Must be updated regularly
Helps prevent missing or overlooking key facts
Does not allow for non-verbal communication
Eaton et al. J Am Coll Surg 2005; 200:538-545.

## Slide 17
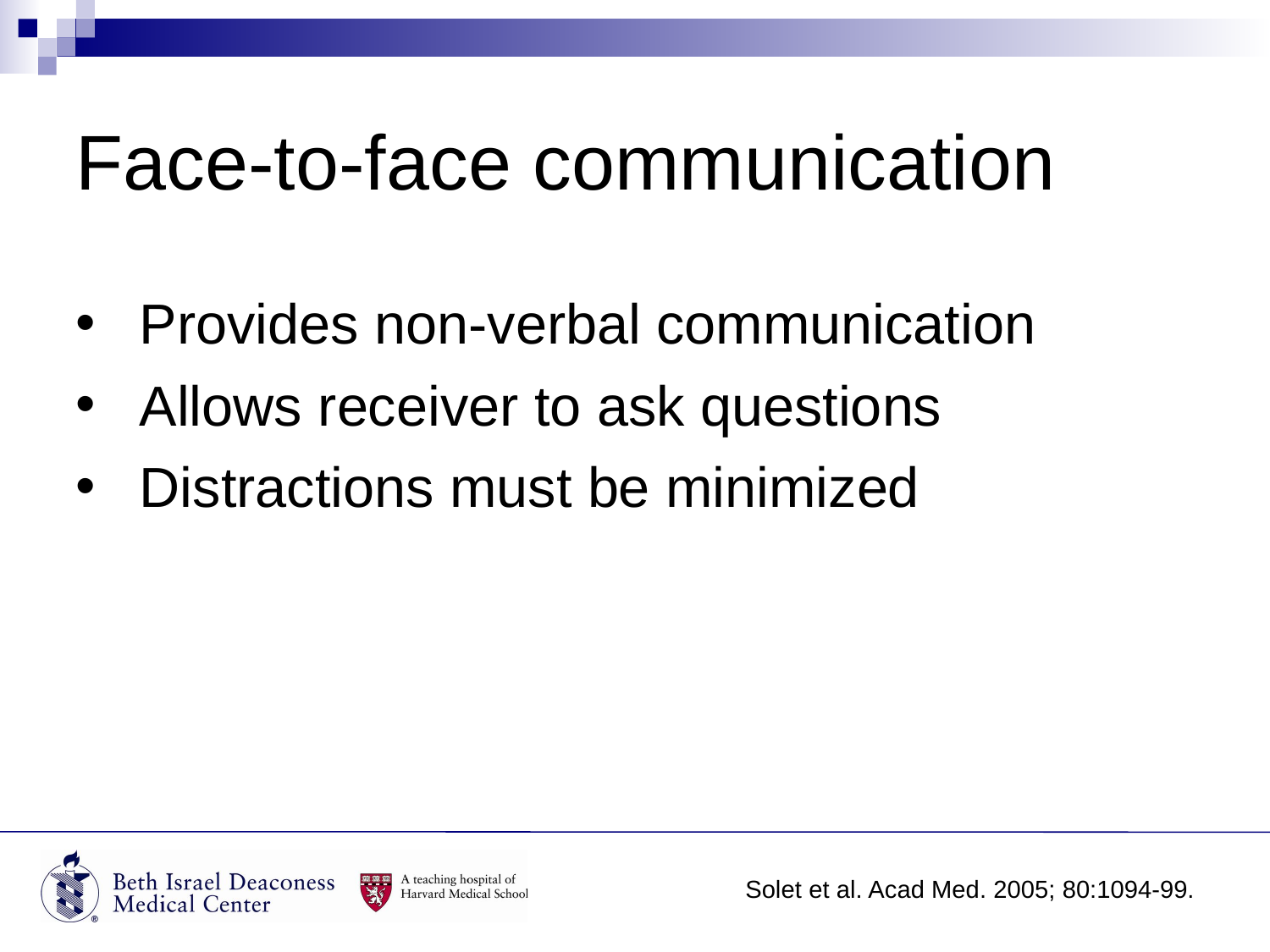

Face-to-face communication
Provides non-verbal communication
Allows receiver to ask questions
Distractions must be minimized
Solet et al. Acad Med. 2005; 80:1094-99.

## Slide 18
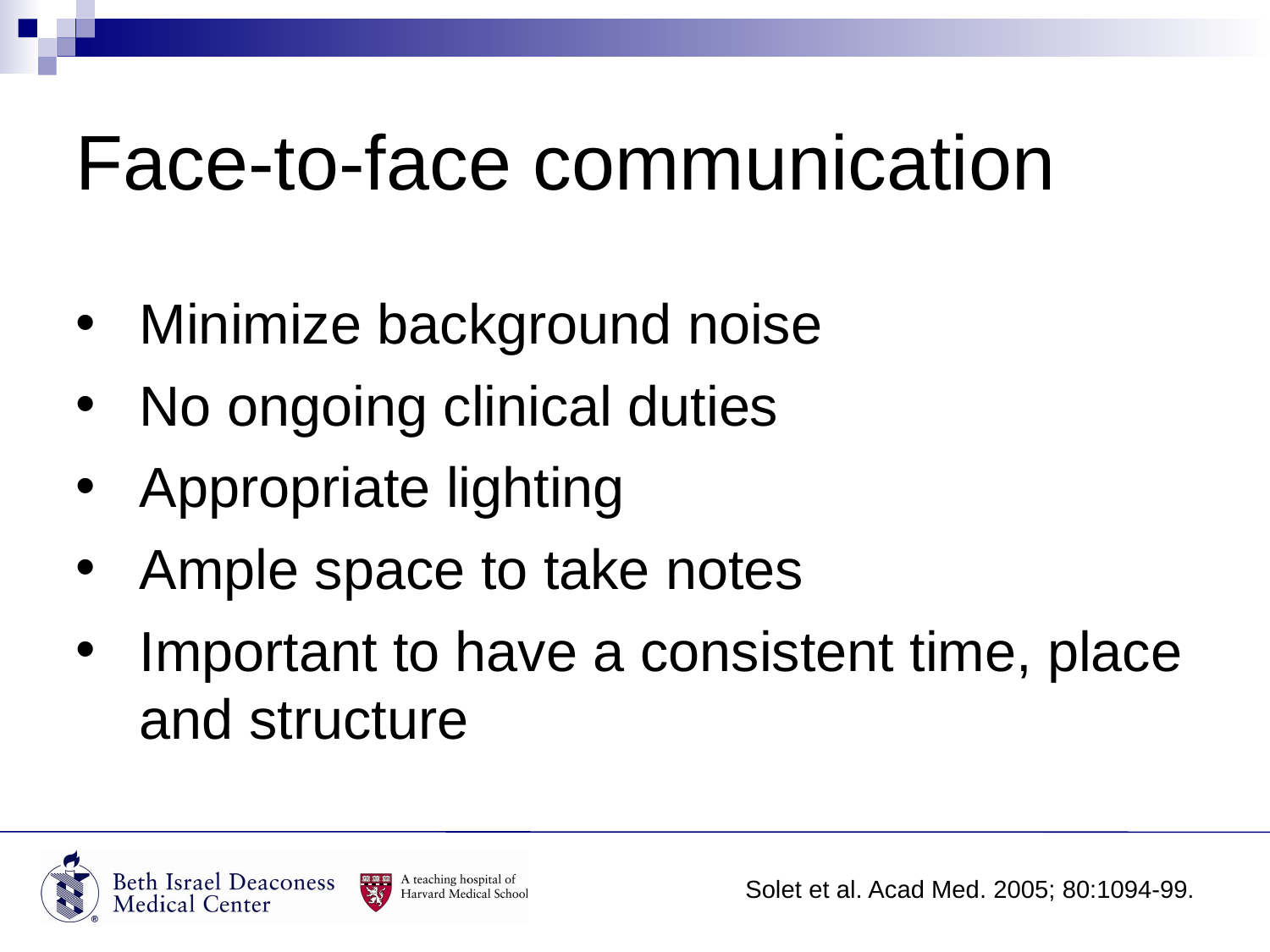

Face-to-face communication
Minimize background noise
No ongoing clinical duties
Appropriate lighting
Ample space to take notes
Important to have a consistent time, place and structure
Solet et al. Acad Med. 2005; 80:1094-99.

## Slide 19
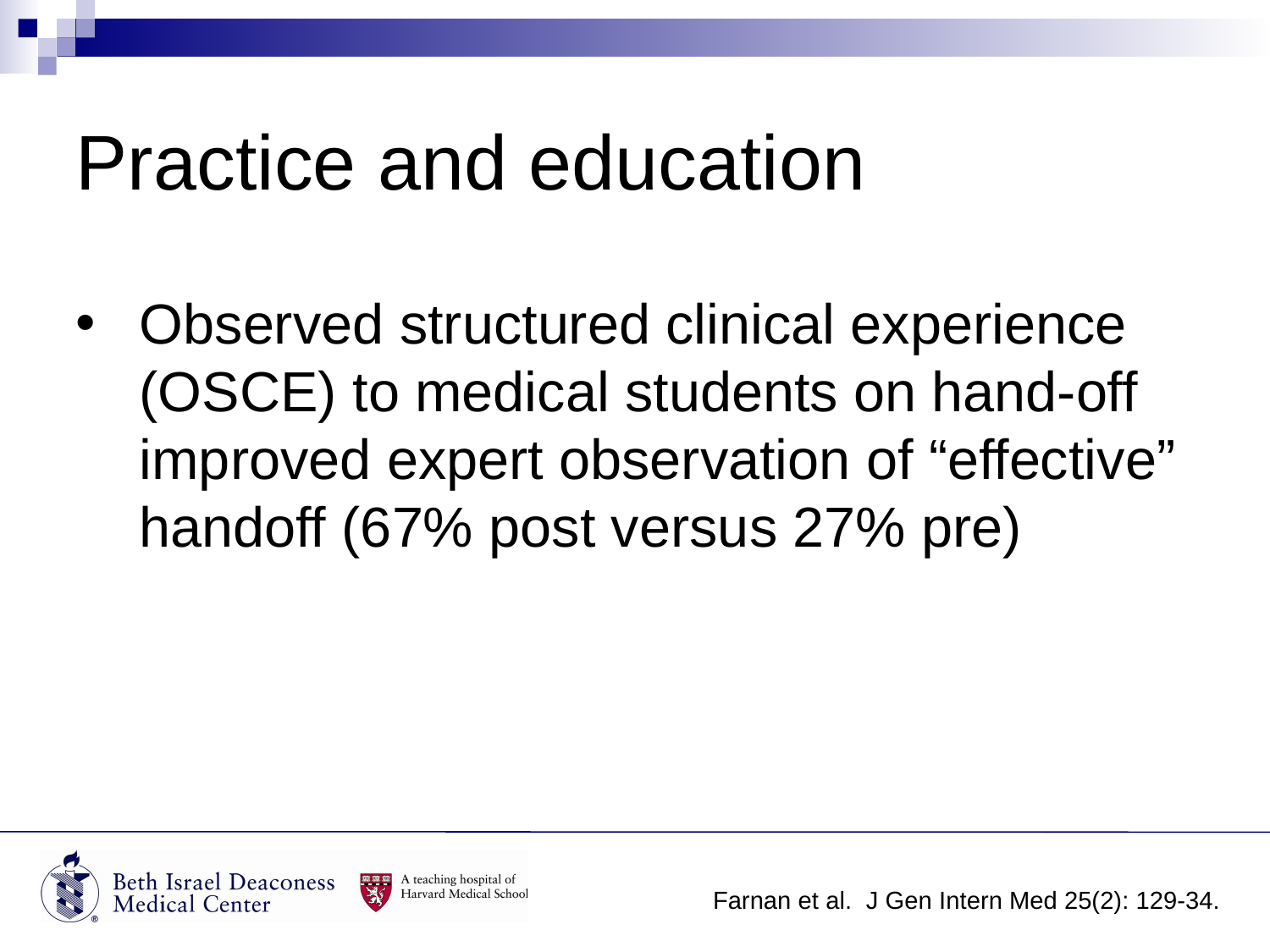

Practice and education
Observed structured clinical experience (OSCE) to medical students on hand-off improved expert observation of “effective” handoff (67% post versus 27% pre)
Farnan et al. J Gen Intern Med 25(2): 129-34.

## Slide 20
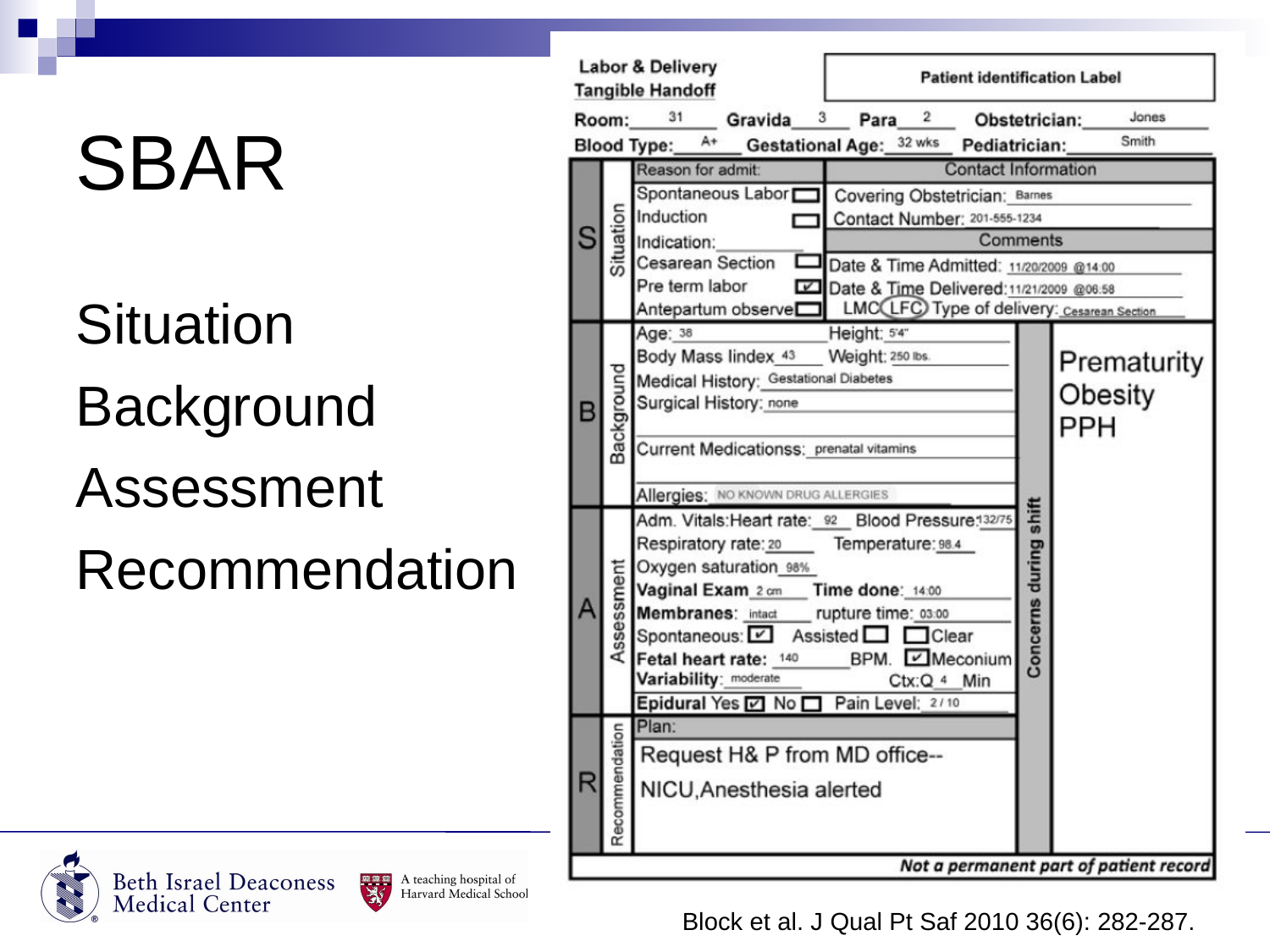

SBAR
Situation
Background
Assessment
Recommendation
Block et al. J Qual Pt Saf 2010 36(6): 282-287.

## Slide 21
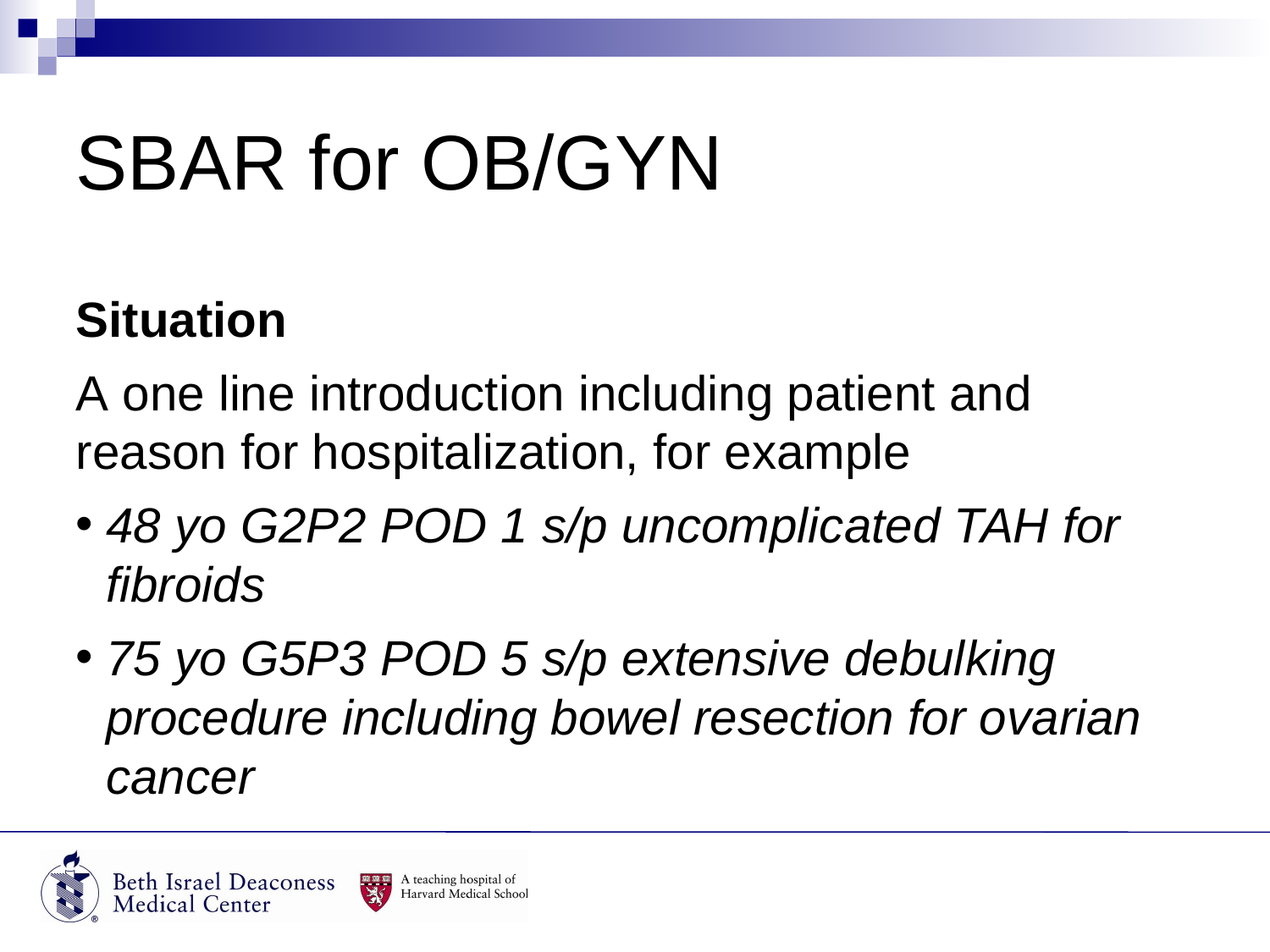

SBAR for OB/GYN
Situation
A one line introduction including patient and reason for hospitalization, for example
48 yo G2P2 POD 1 s/p uncomplicated TAH for fibroids
75 yo G5P3 POD 5 s/p extensive debulking procedure including bowel resection for ovarian cancer

## Slide 22
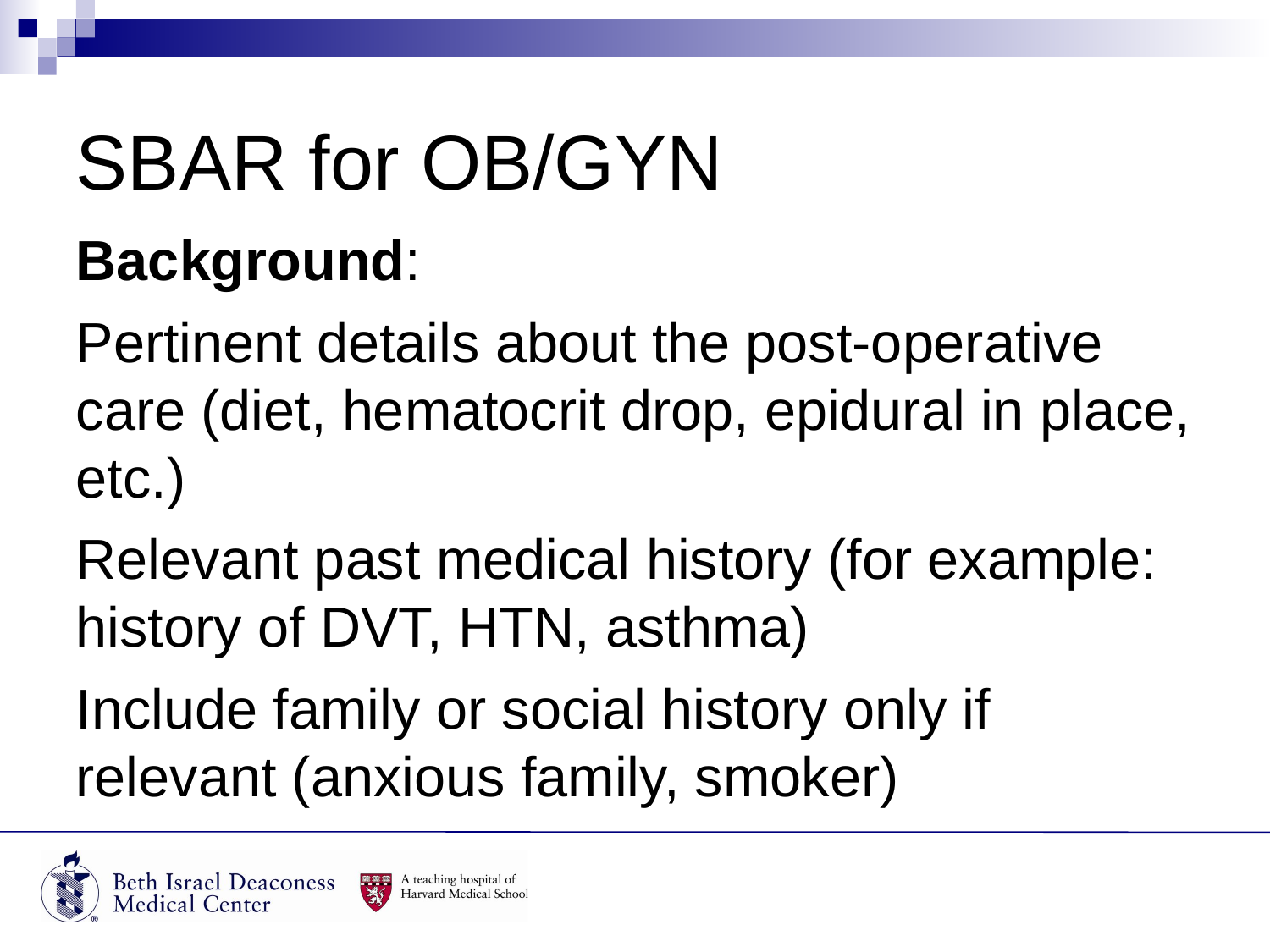

SBAR for OB/GYN
Background:
Pertinent details about the post-operative care (diet, hematocrit drop, epidural in place, etc.)
Relevant past medical history (for example: history of DVT, HTN, asthma)
Include family or social history only if relevant (anxious family, smoker)

## Slide 23
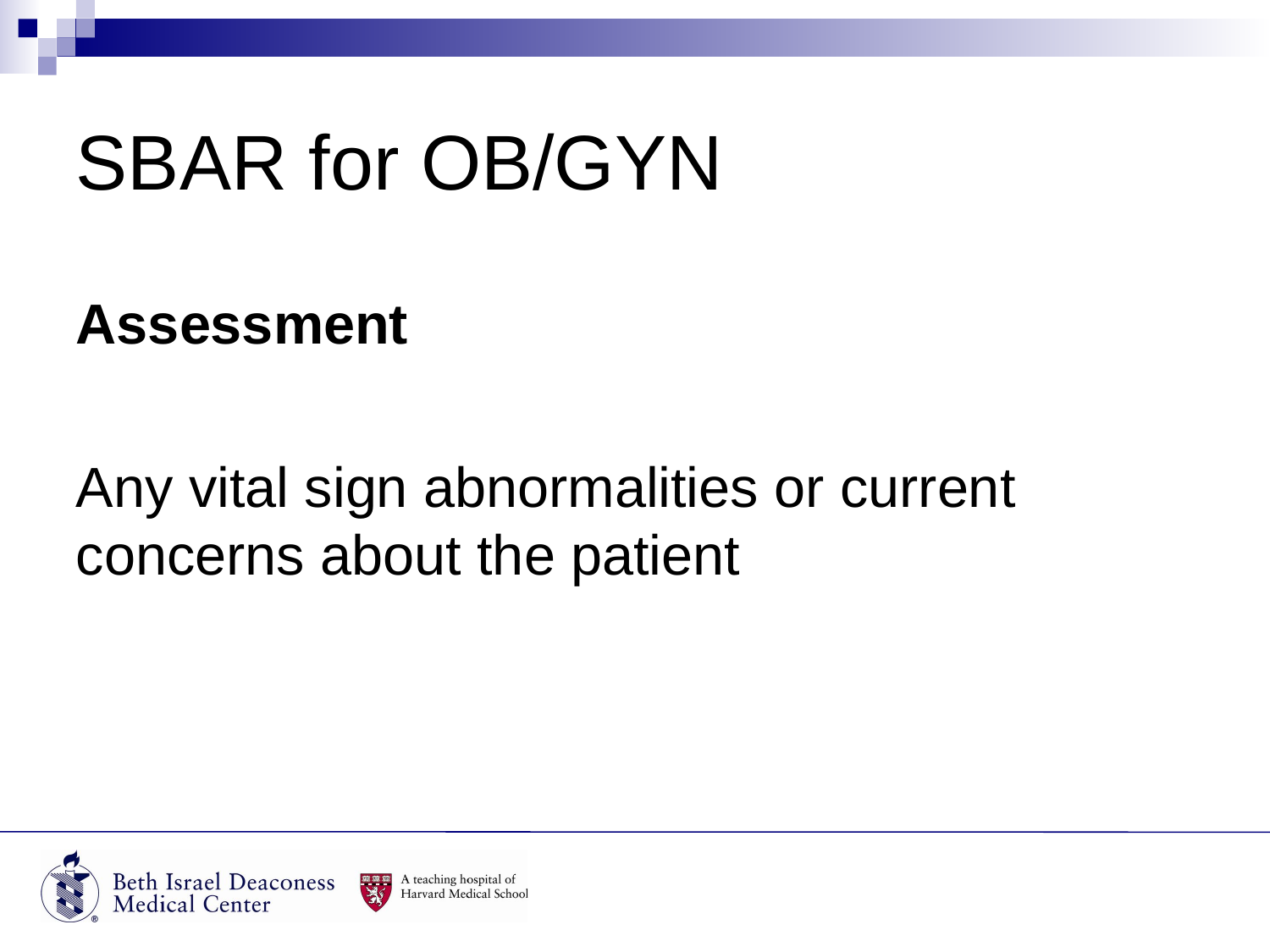

SBAR for OB/GYN
Assessment
Any vital sign abnormalities or current concerns about the patient

## Slide 24
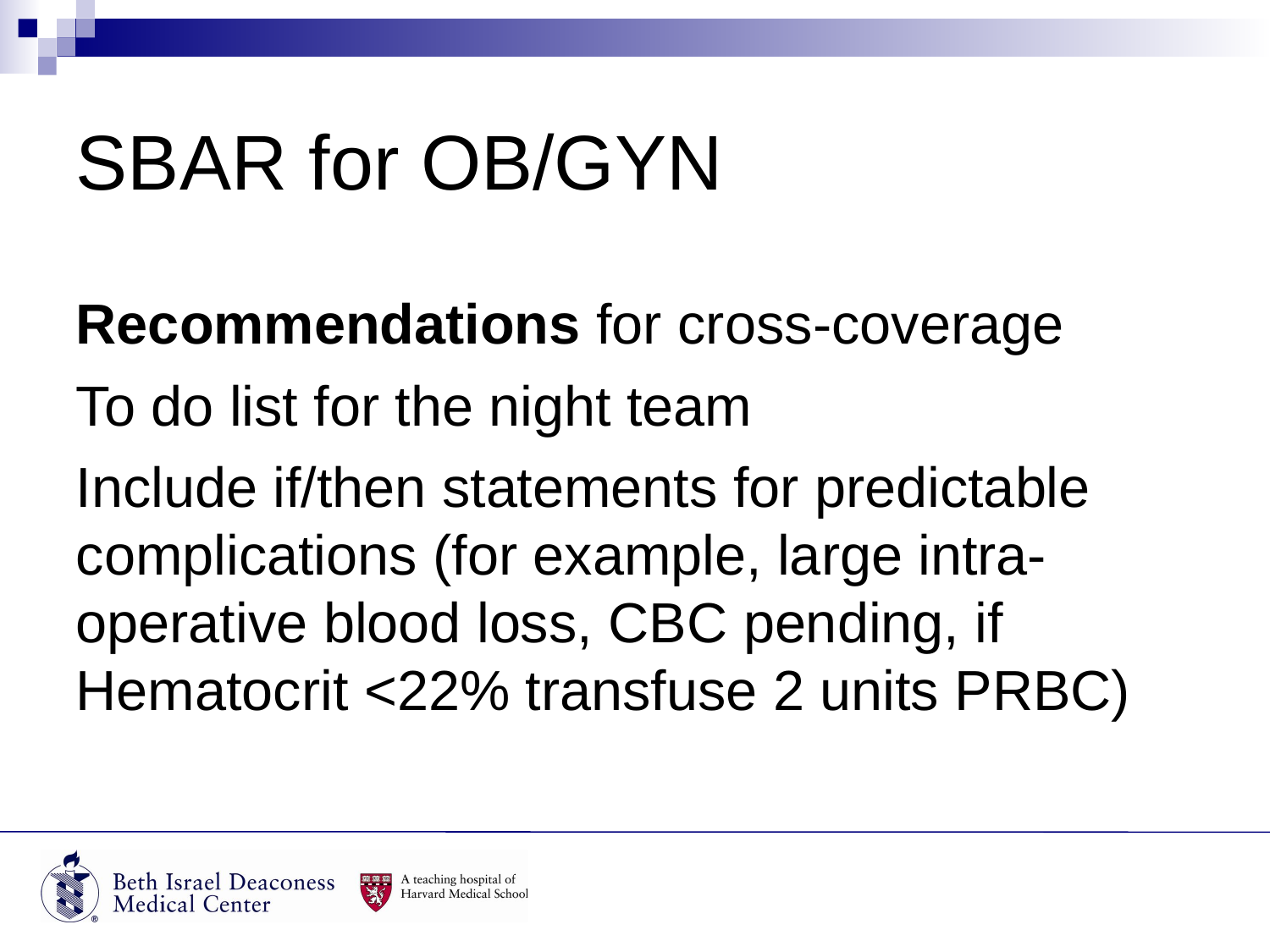

SBAR for OB/GYN
Recommendations for cross-coverage
To do list for the night team
Include if/then statements for predictable complications (for example, large intra-operative blood loss, CBC pending, if Hematocrit <22% transfuse 2 units PRBC)

## Slide 25
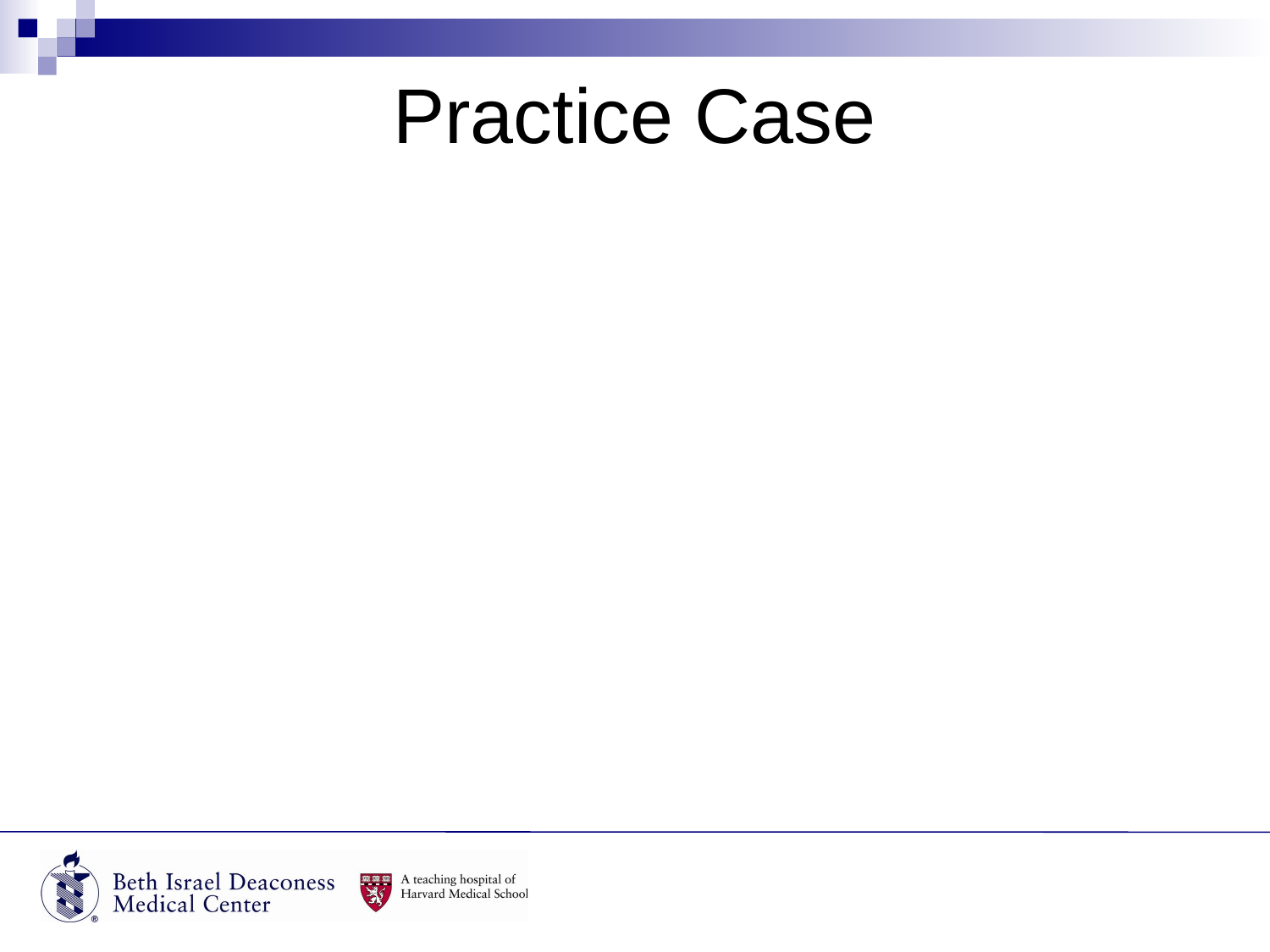

Practice Case

## Slide 26
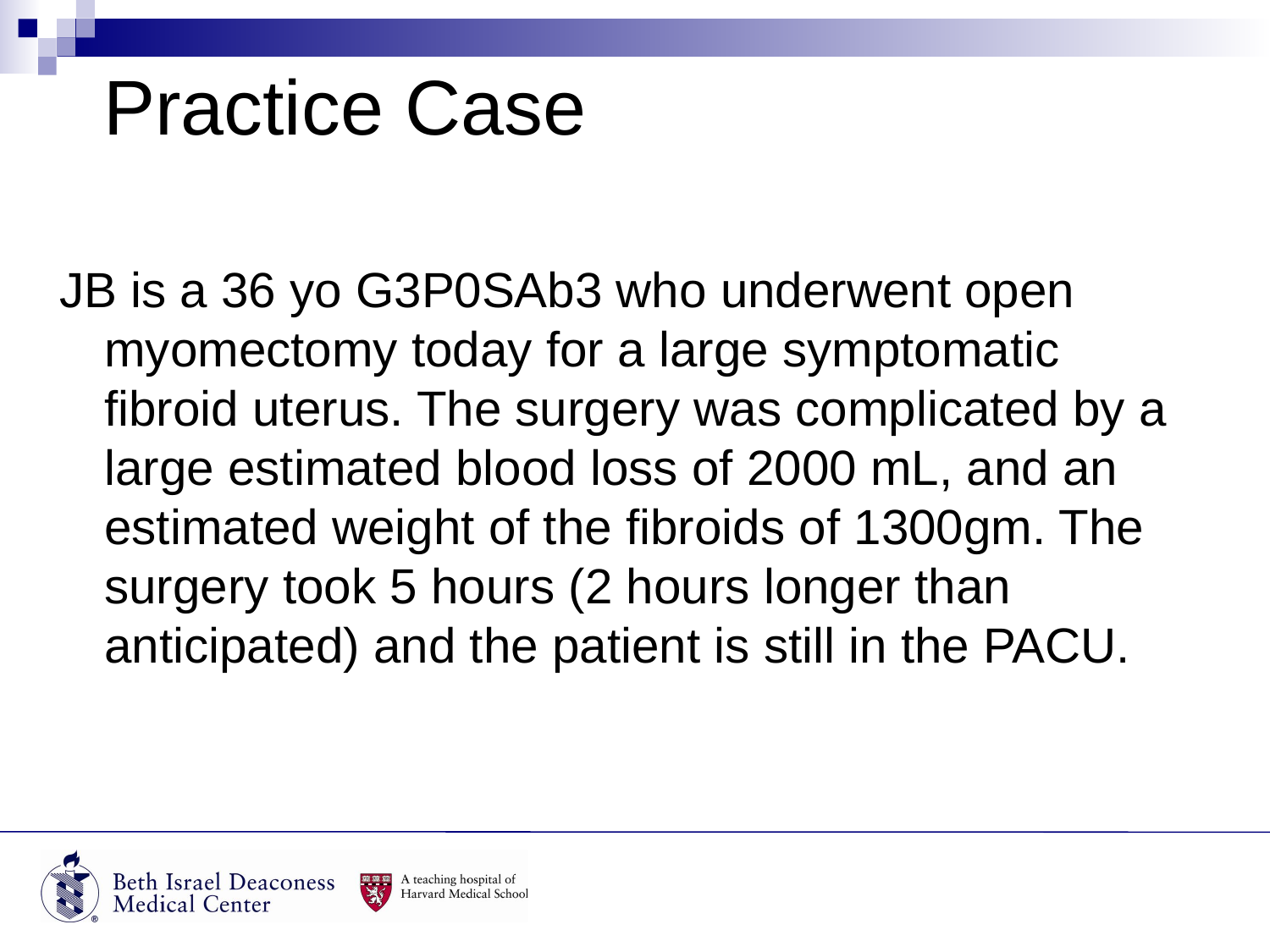

# Practice Case
JB is a 36 yo G3P0SAb3 who underwent open myomectomy today for a large symptomatic fibroid uterus. The surgery was complicated by a large estimated blood loss of 2000 mL, and an estimated weight of the fibroids of 1300gm. The surgery took 5 hours (2 hours longer than anticipated) and the patient is still in the PACU.

## Slide 27
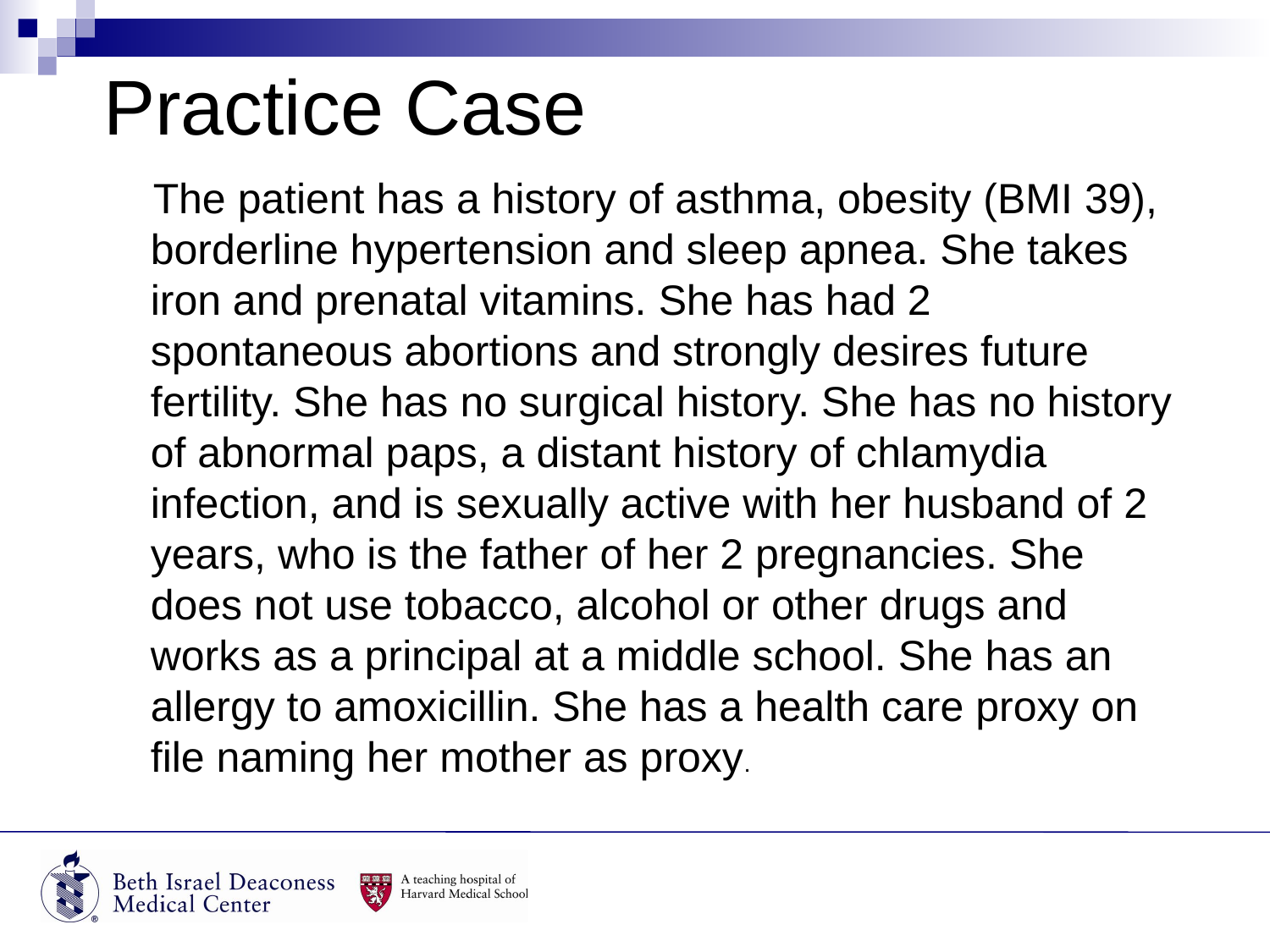

# Practice Case
 The patient has a history of asthma, obesity (BMI 39), borderline hypertension and sleep apnea. She takes iron and prenatal vitamins. She has had 2 spontaneous abortions and strongly desires future fertility. She has no surgical history. She has no history of abnormal paps, a distant history of chlamydia infection, and is sexually active with her husband of 2 years, who is the father of her 2 pregnancies. She does not use tobacco, alcohol or other drugs and works as a principal at a middle school. She has an allergy to amoxicillin. She has a health care proxy on file naming her mother as proxy.

## Slide 28
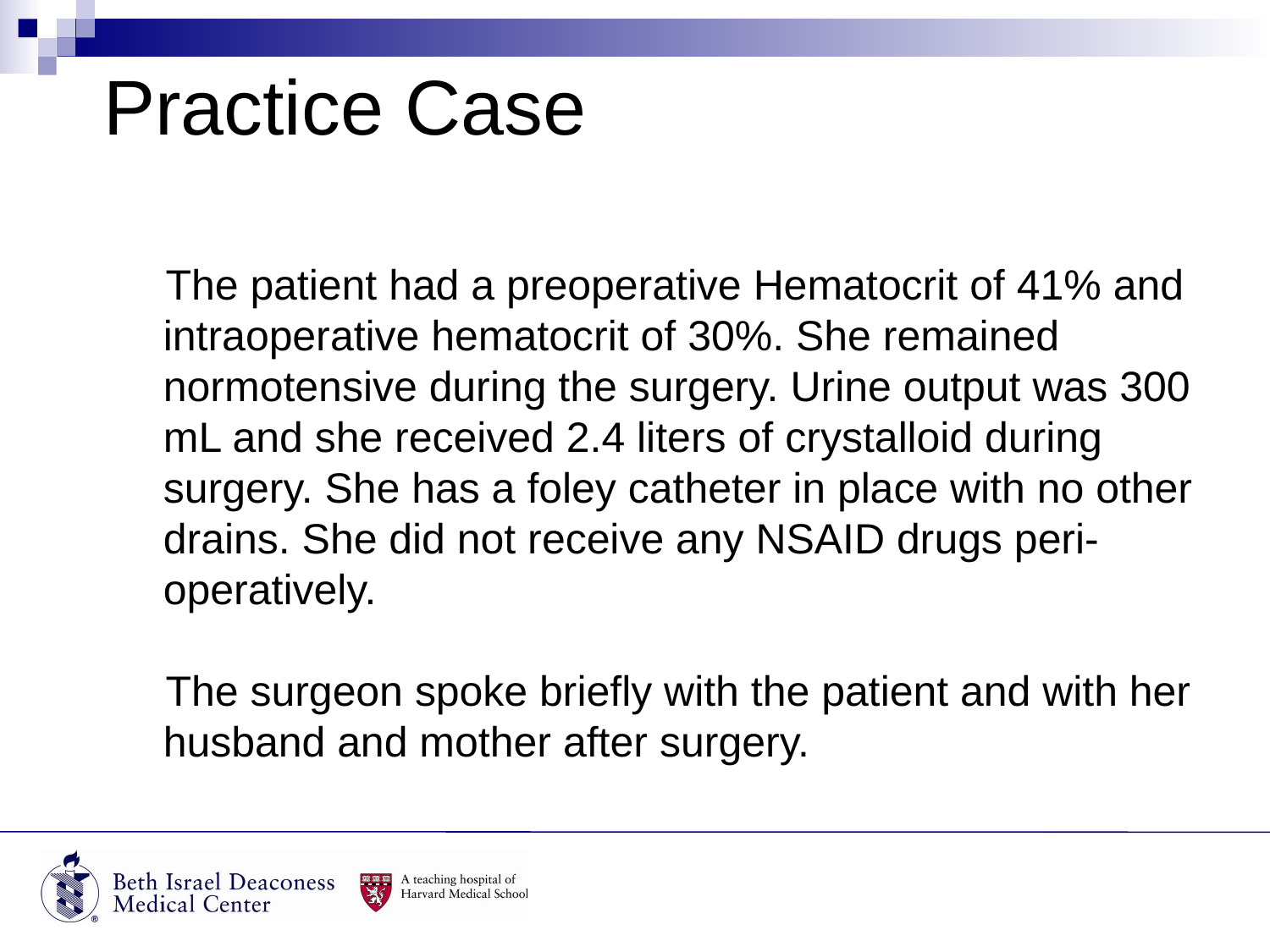

# Practice Case
 The patient had a preoperative Hematocrit of 41% and intraoperative hematocrit of 30%. She remained normotensive during the surgery. Urine output was 300 mL and she received 2.4 liters of crystalloid during surgery. She has a foley catheter in place with no other drains. She did not receive any NSAID drugs peri-operatively.
 The surgeon spoke briefly with the patient and with her husband and mother after surgery.

## Slide 29
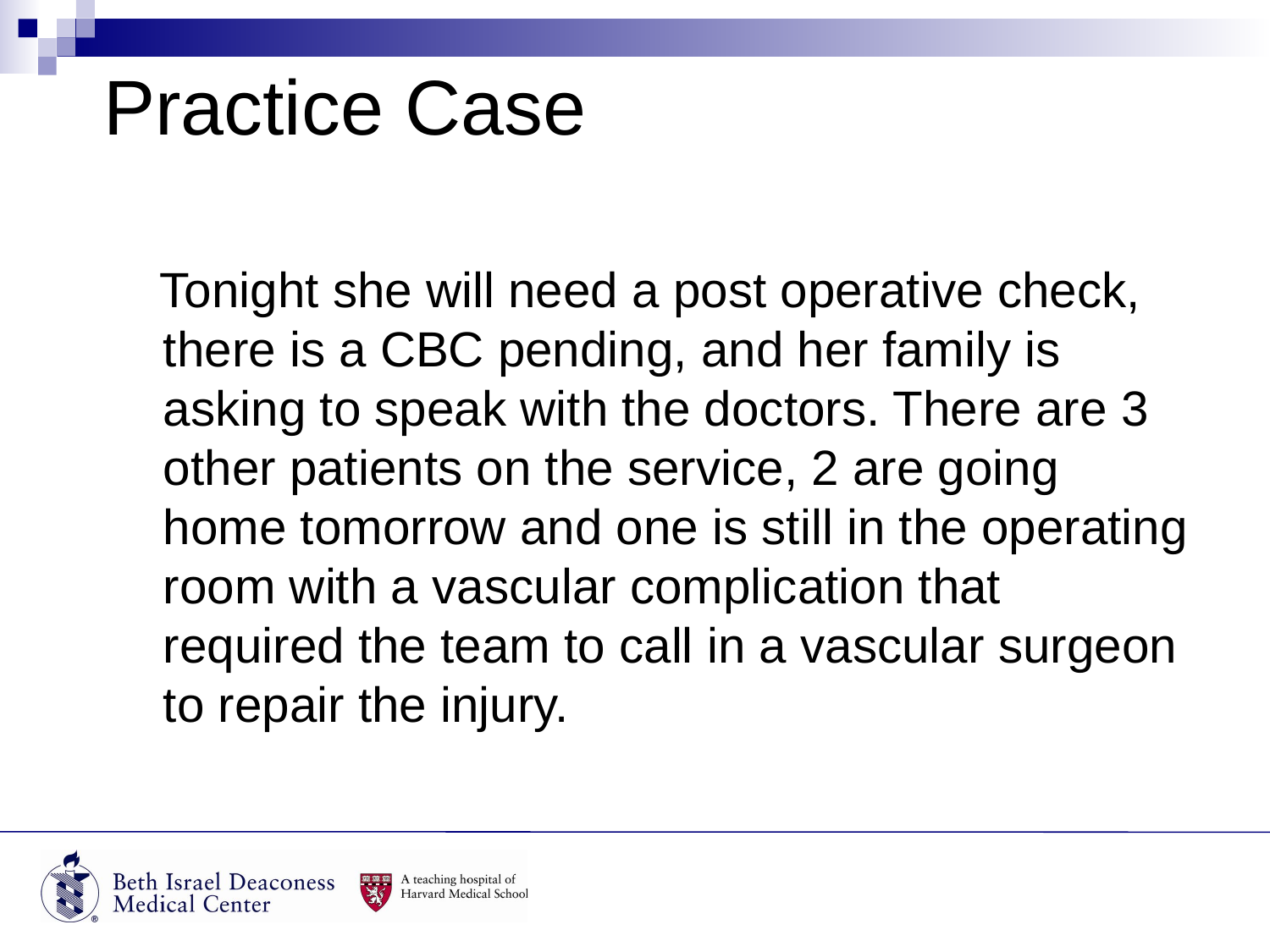

# Practice Case
 Tonight she will need a post operative check, there is a CBC pending, and her family is asking to speak with the doctors. There are 3 other patients on the service, 2 are going home tomorrow and one is still in the operating room with a vascular complication that required the team to call in a vascular surgeon to repair the injury.

## Slide 30
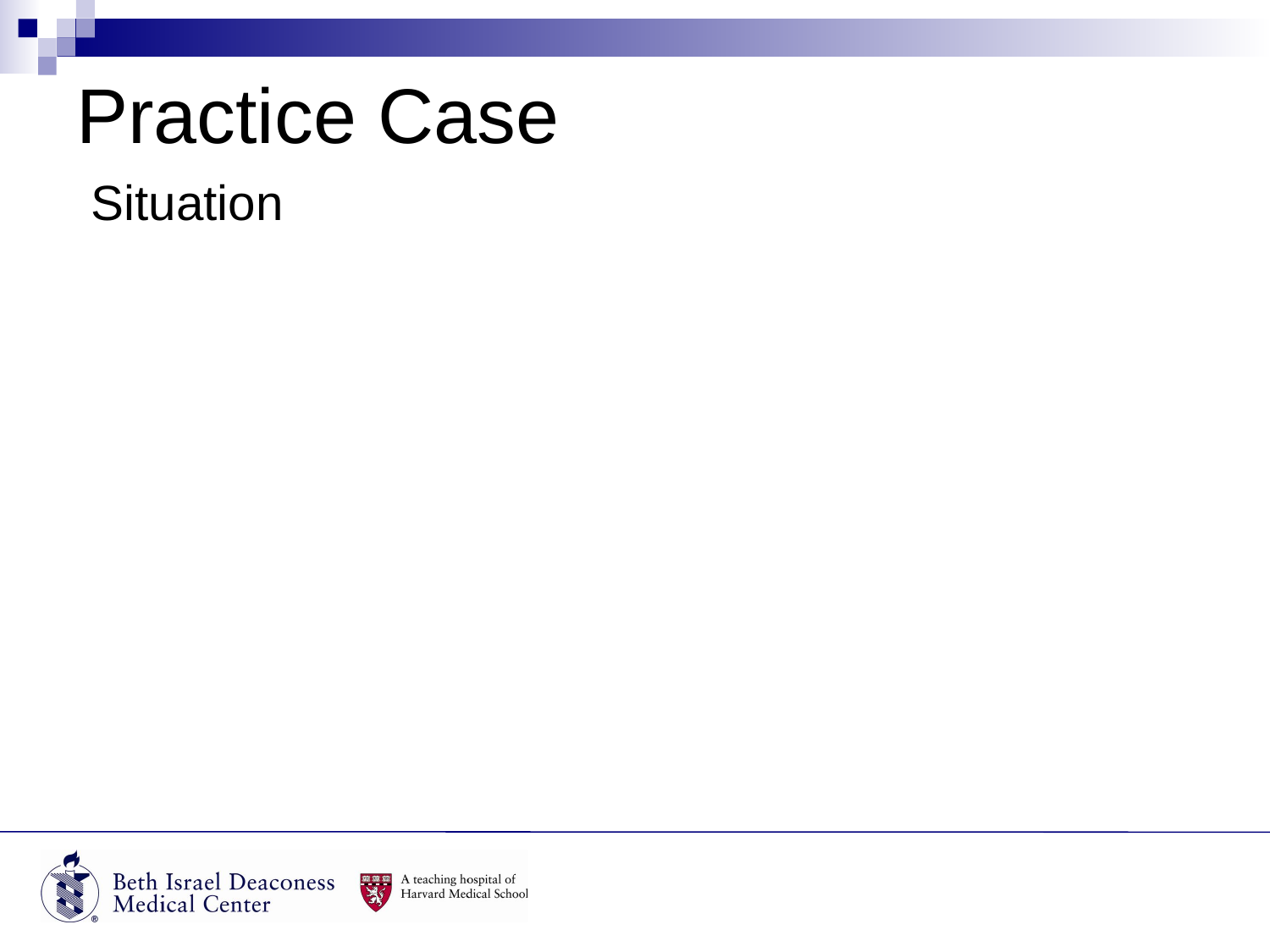

# Practice Case
Situation

## Slide 31
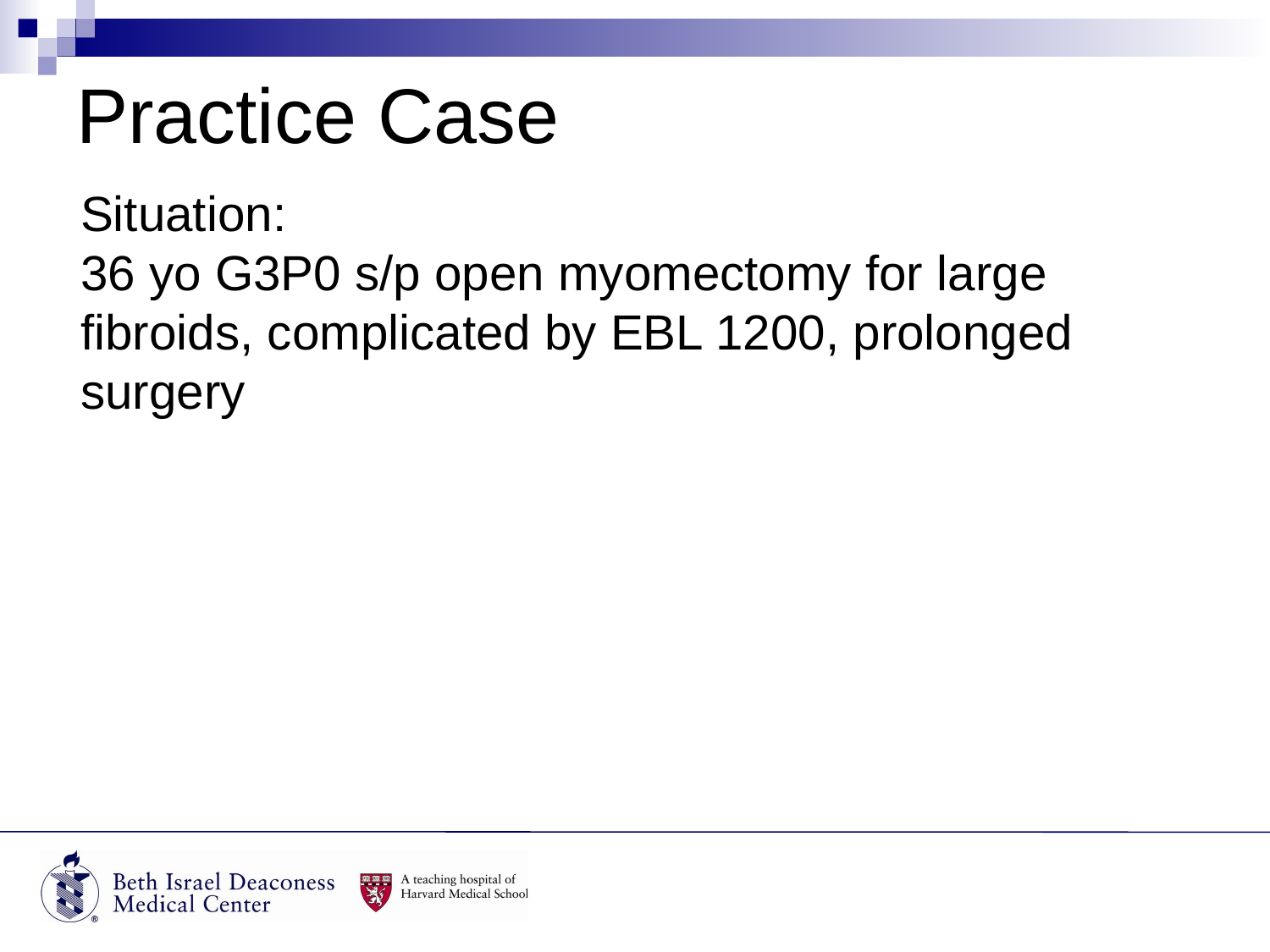

# Practice Case
Situation:
36 yo G3P0 s/p open myomectomy for large fibroids, complicated by EBL 1200, prolonged surgery

## Slide 32
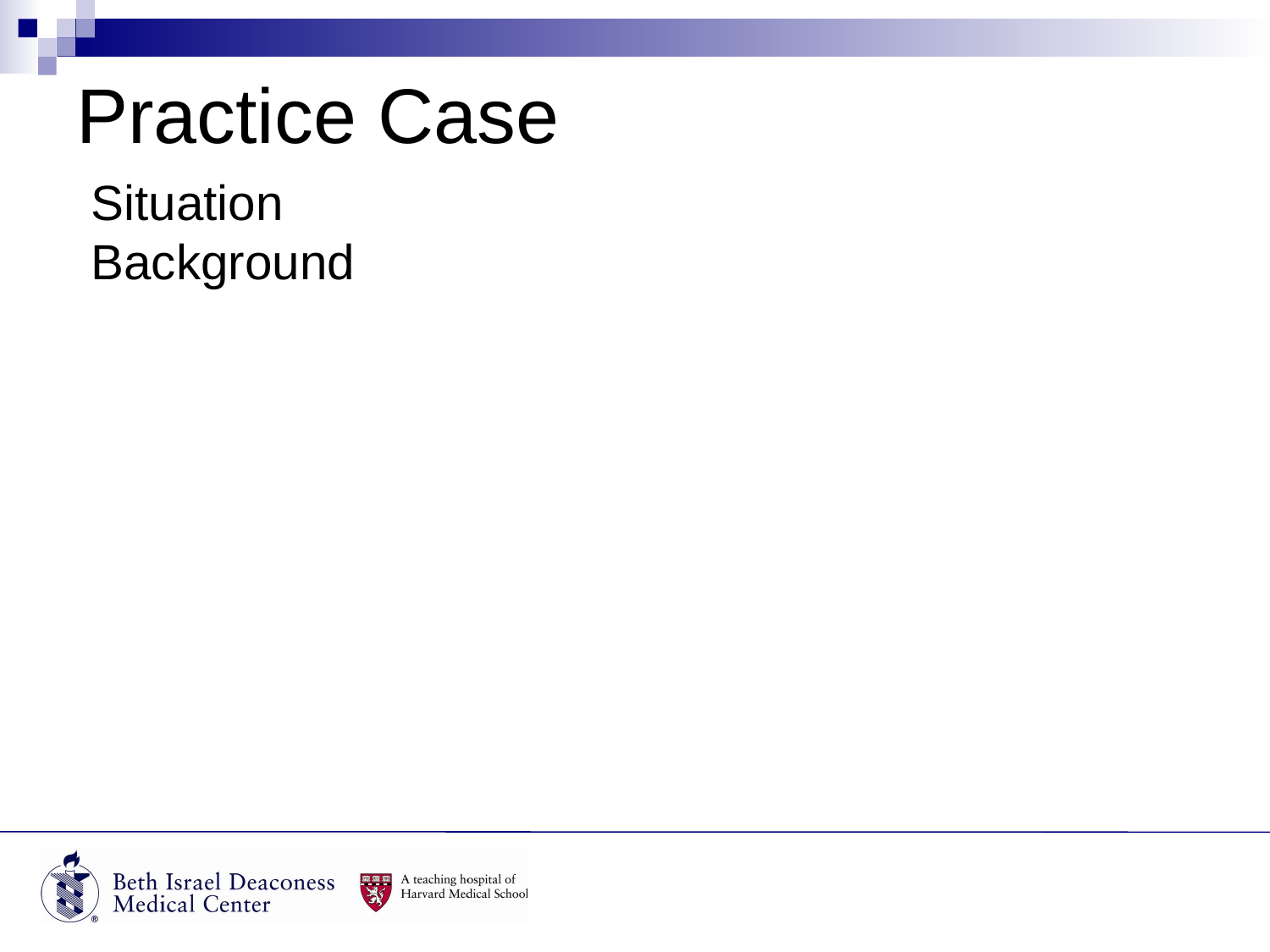

# Practice Case
Situation
Background

## Slide 33
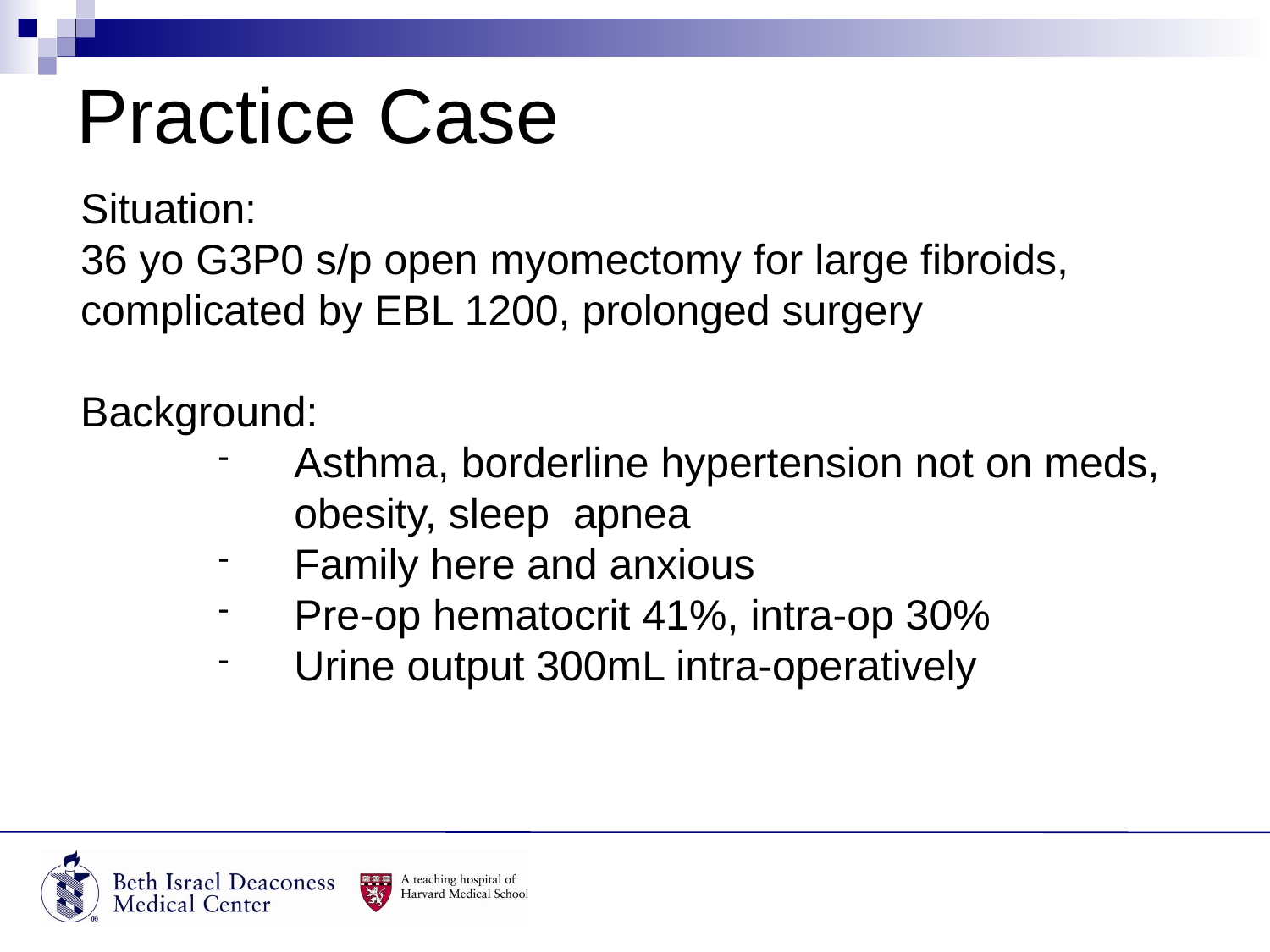

# Practice Case
Situation:
36 yo G3P0 s/p open myomectomy for large fibroids, complicated by EBL 1200, prolonged surgery
Background:
Asthma, borderline hypertension not on meds, obesity, sleep apnea
Family here and anxious
Pre-op hematocrit 41%, intra-op 30%
Urine output 300mL intra-operatively

## Slide 34
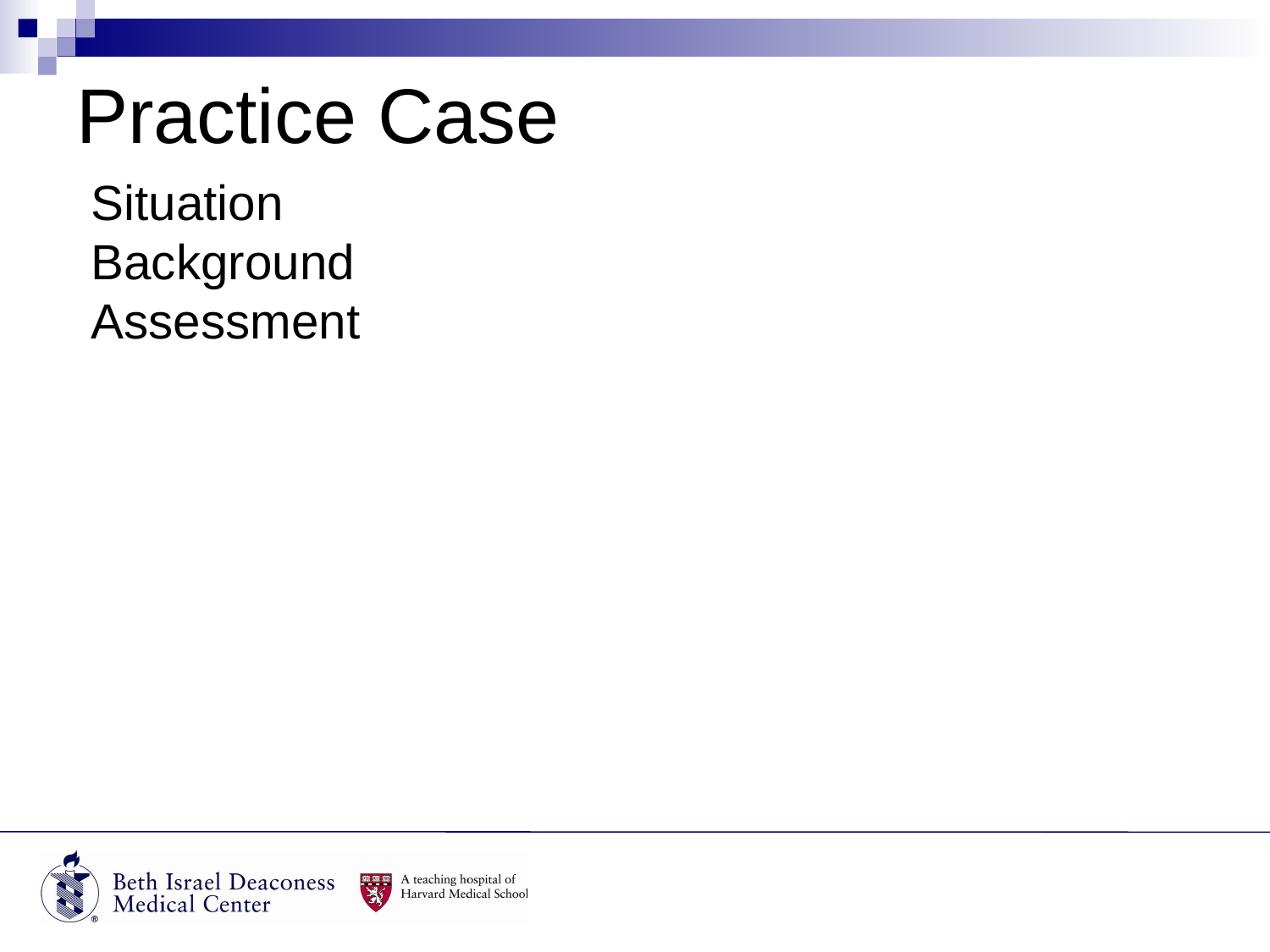

# Practice Case
Situation
Background
Assessment

## Slide 35
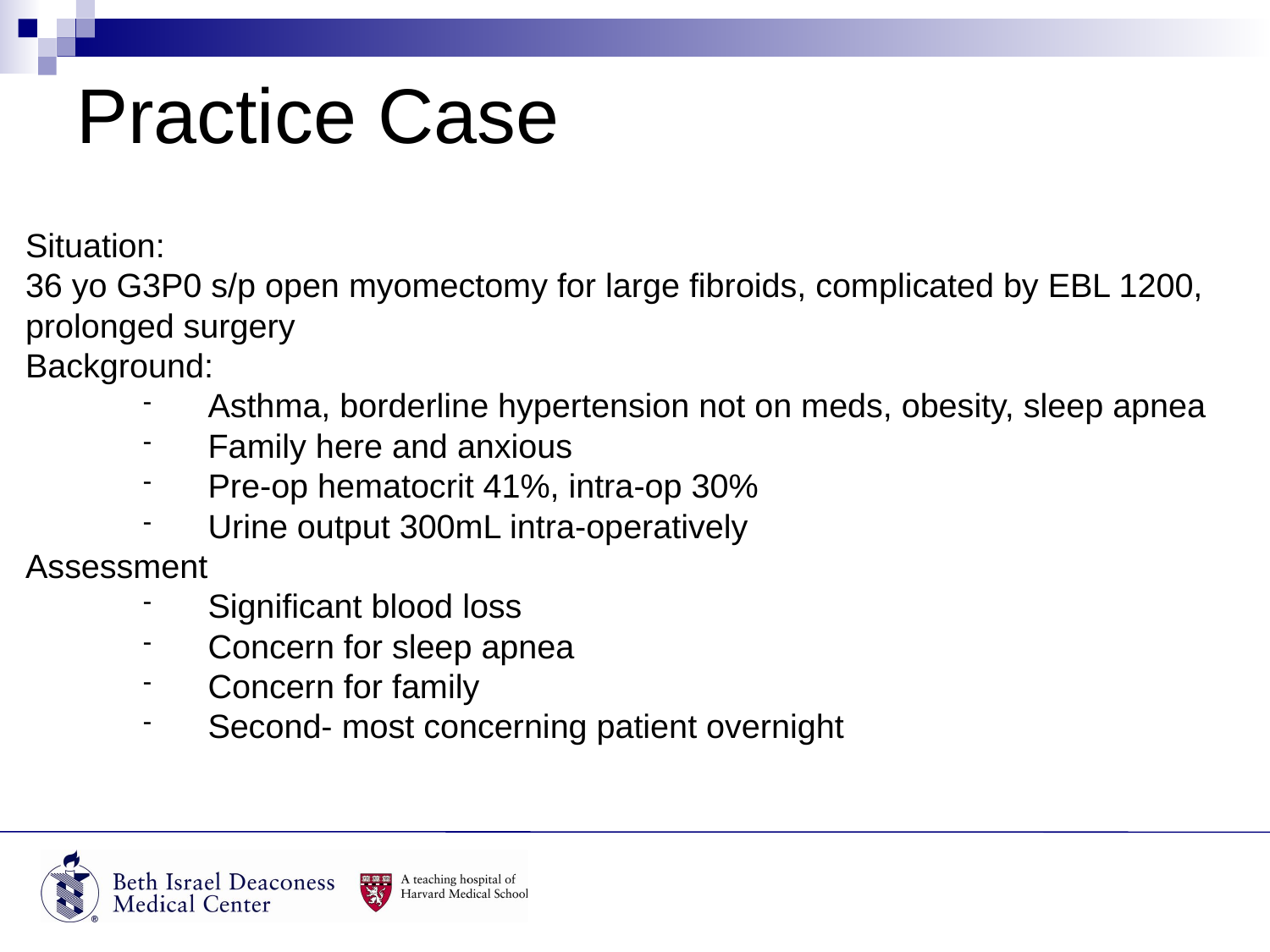

# Practice Case
Situation:
36 yo G3P0 s/p open myomectomy for large fibroids, complicated by EBL 1200, prolonged surgery
Background:
Asthma, borderline hypertension not on meds, obesity, sleep apnea
Family here and anxious
Pre-op hematocrit 41%, intra-op 30%
Urine output 300mL intra-operatively
Assessment
Significant blood loss
Concern for sleep apnea
Concern for family
Second- most concerning patient overnight

## Slide 36
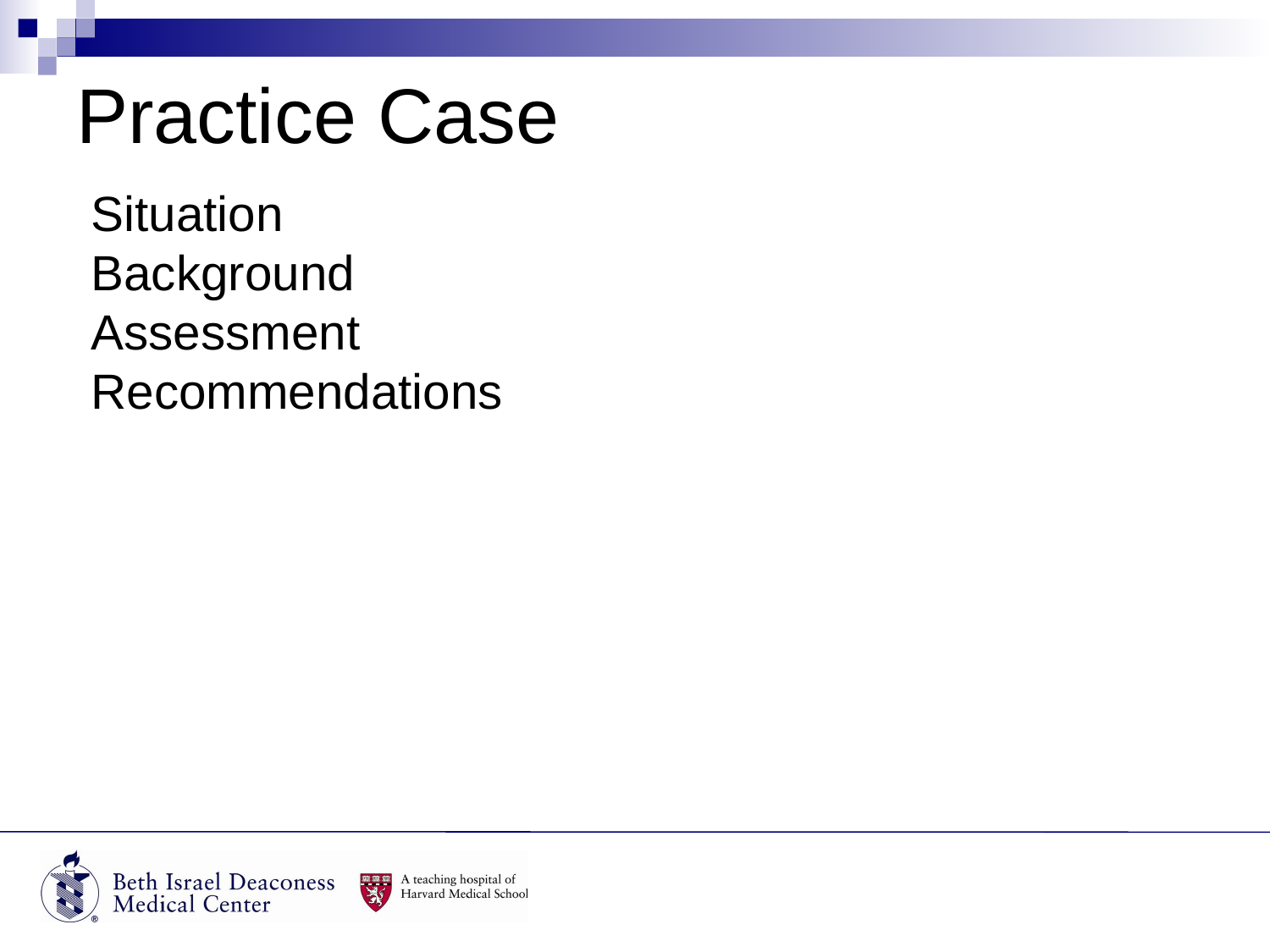

# Practice Case
Situation
Background
Assessment
Recommendations

## Slide 37
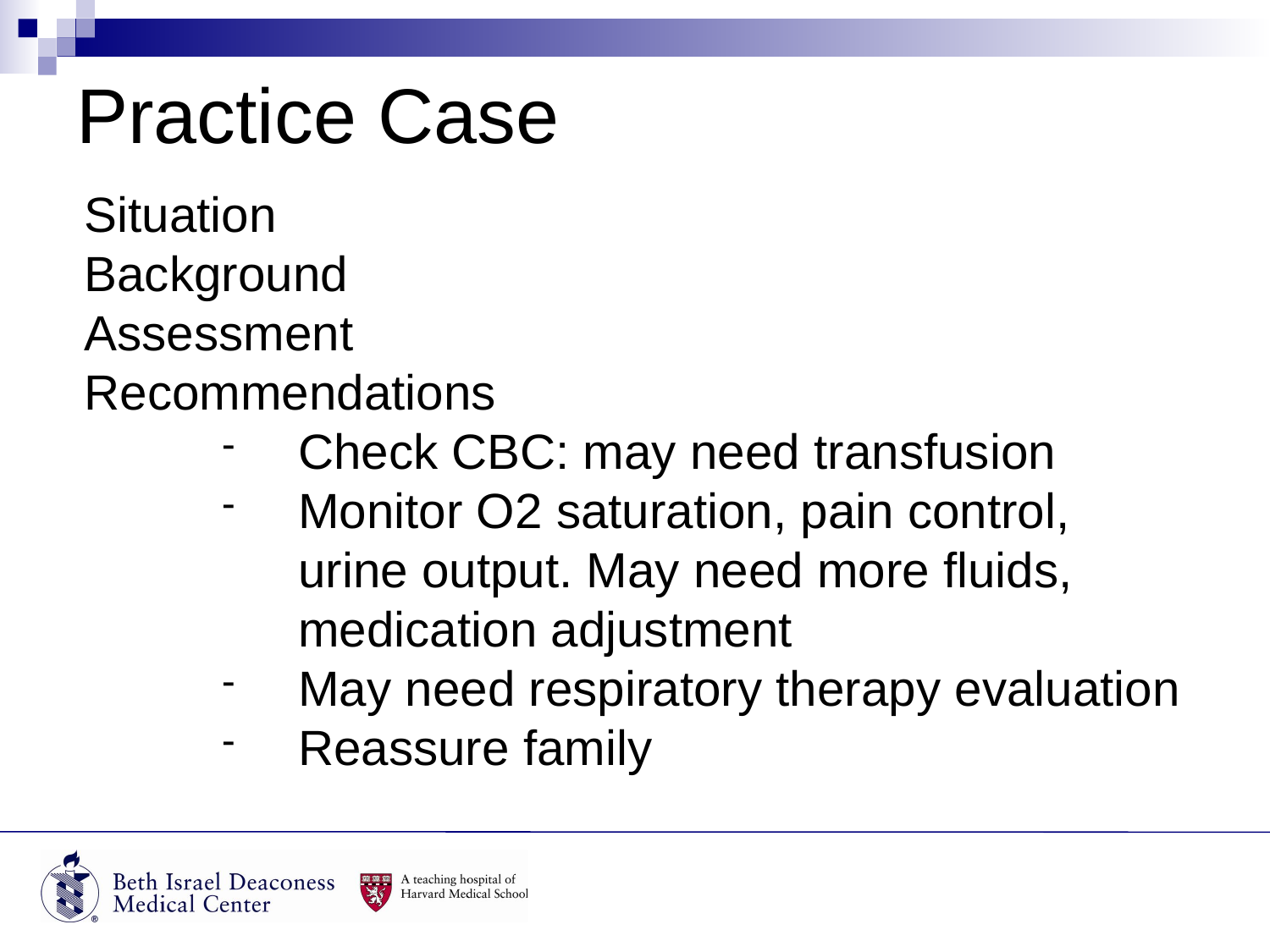

# Practice Case
Situation
Background
Assessment
Recommendations
Check CBC: may need transfusion
Monitor O2 saturation, pain control, urine output. May need more fluids, medication adjustment
May need respiratory therapy evaluation
Reassure family
