# Supplementary material for: Teaching Patient Handoffs to Medical Students in Obstetrics and Gynecology: Simulation Curriculum and Assessment Tool
Source: MedEdPORTAL. 2016 Oct 2;12:10479. doi: 10.15766/mep_2374-8265.10479 (PMC6440488; doi:10.15766/mep_2374-8265.10479)
Supplement: Supplementary file 1 — A. Patient Handoffs in Obstetrics and Gynecology.pptx B. Approach to Diagnosis and Management of First Trimester Bleeding.pptx C. Patient Handoffs in Obstetrics and Gynecology Narrated.mp4 D. Approach to Diagnosis and Management of First Trimester Bleeding Narrated.mp4 E. Handoff Skills Speakers Notes.docx F. First Trimester Bleeding Speakers Notes.docx G. Simulation Guide.docx H. Role Play Description.docx I. Trainee Simulation Information Cards.doc J. Ultrasound Report.docx K. Student Assessment Tool.docx L. Debrief Checklists.docx [file mep-12-10479-s001.zip › B. Approach to Diagnosis and Management of First Trimester Bleeding.pptx]

## Slide 1
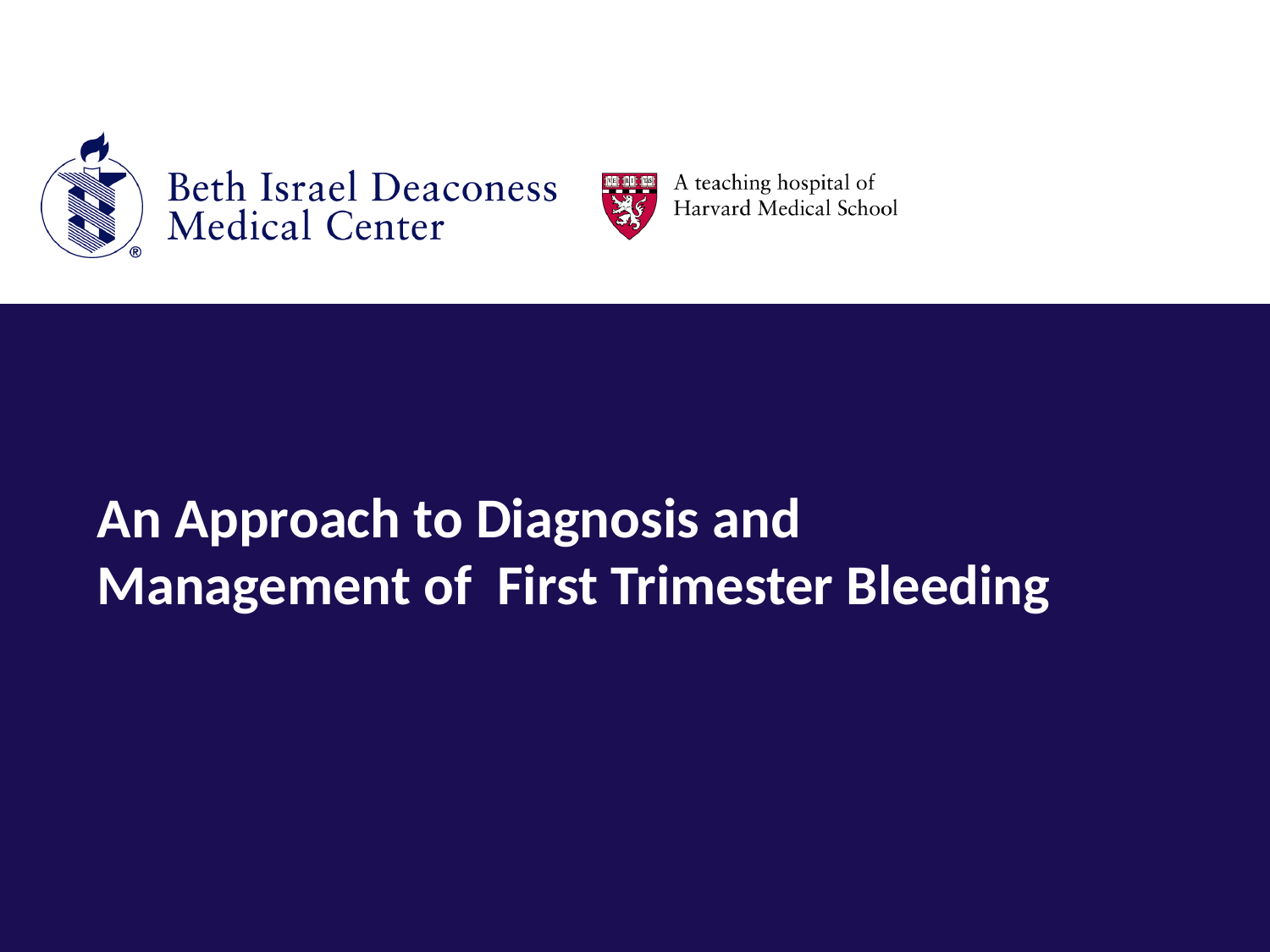

# An Approach to Diagnosis and Management of First Trimester Bleeding

## Slide 2
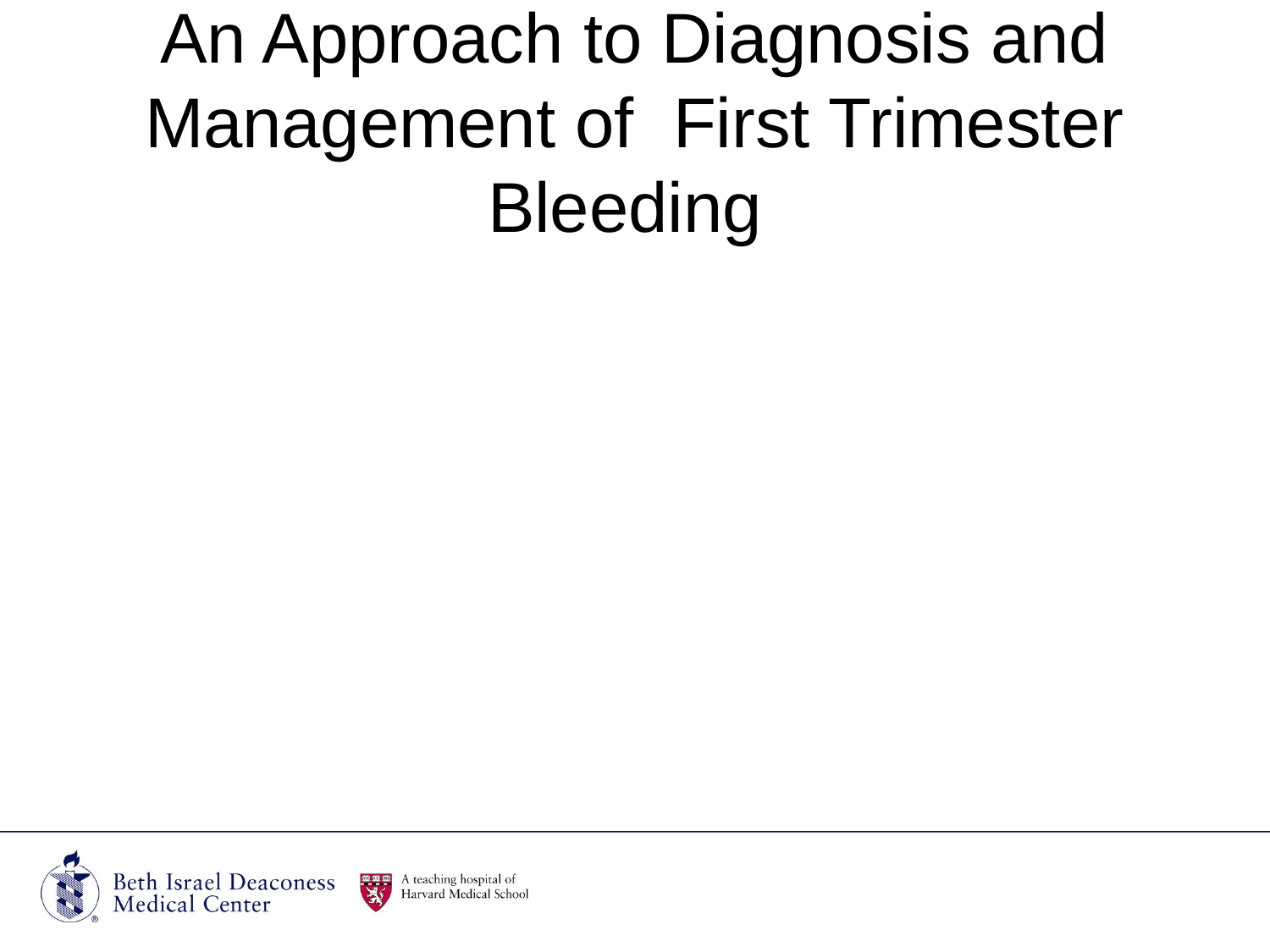

An Approach to Diagnosis and Management of First Trimester Bleeding

## Slide 3
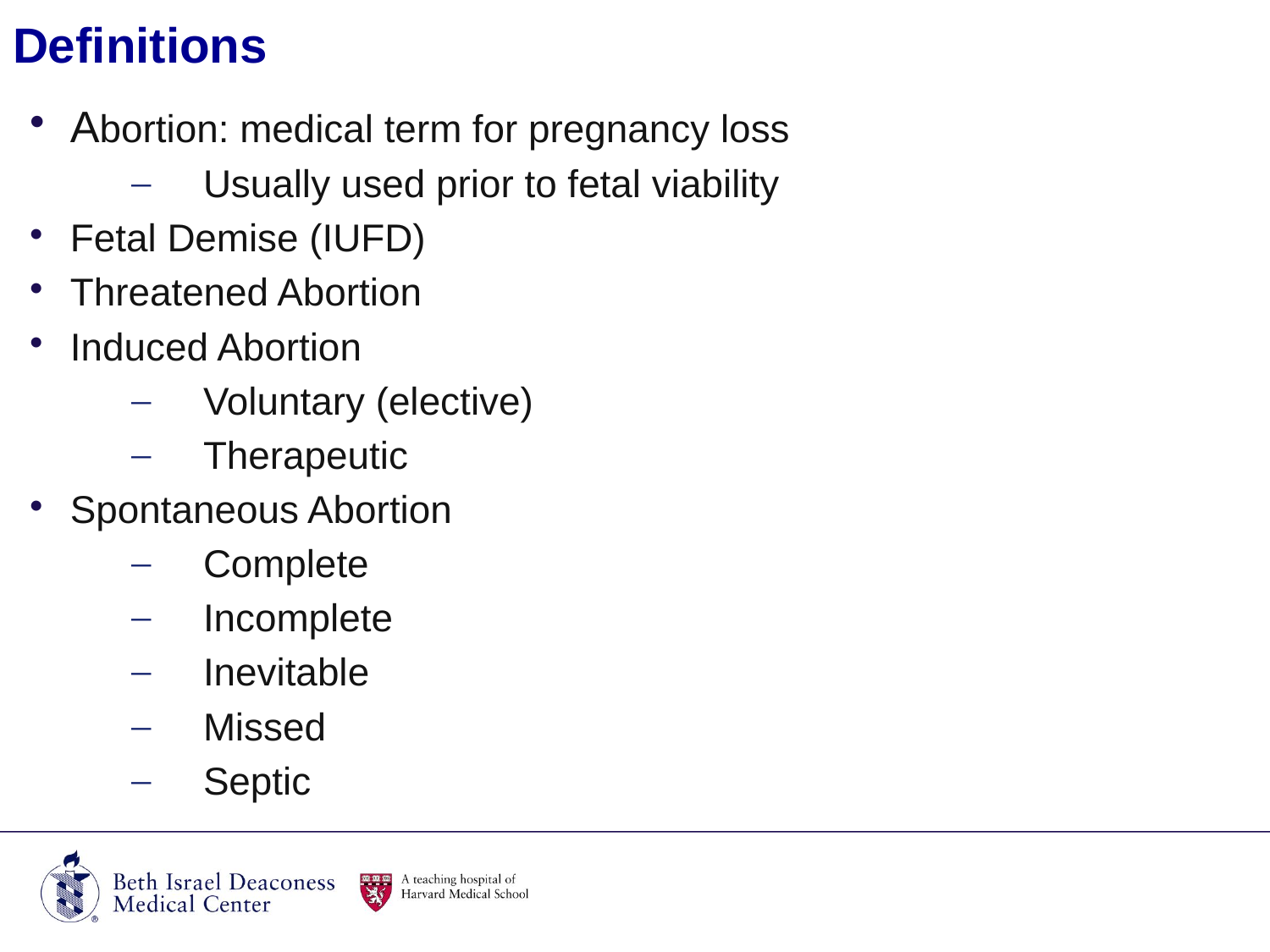

# Definitions
Abortion: medical term for pregnancy loss
Usually used prior to fetal viability
Fetal Demise (IUFD)
Threatened Abortion
Induced Abortion
Voluntary (elective)
Therapeutic
Spontaneous Abortion
Complete
Incomplete
Inevitable
Missed
Septic

## Slide 4
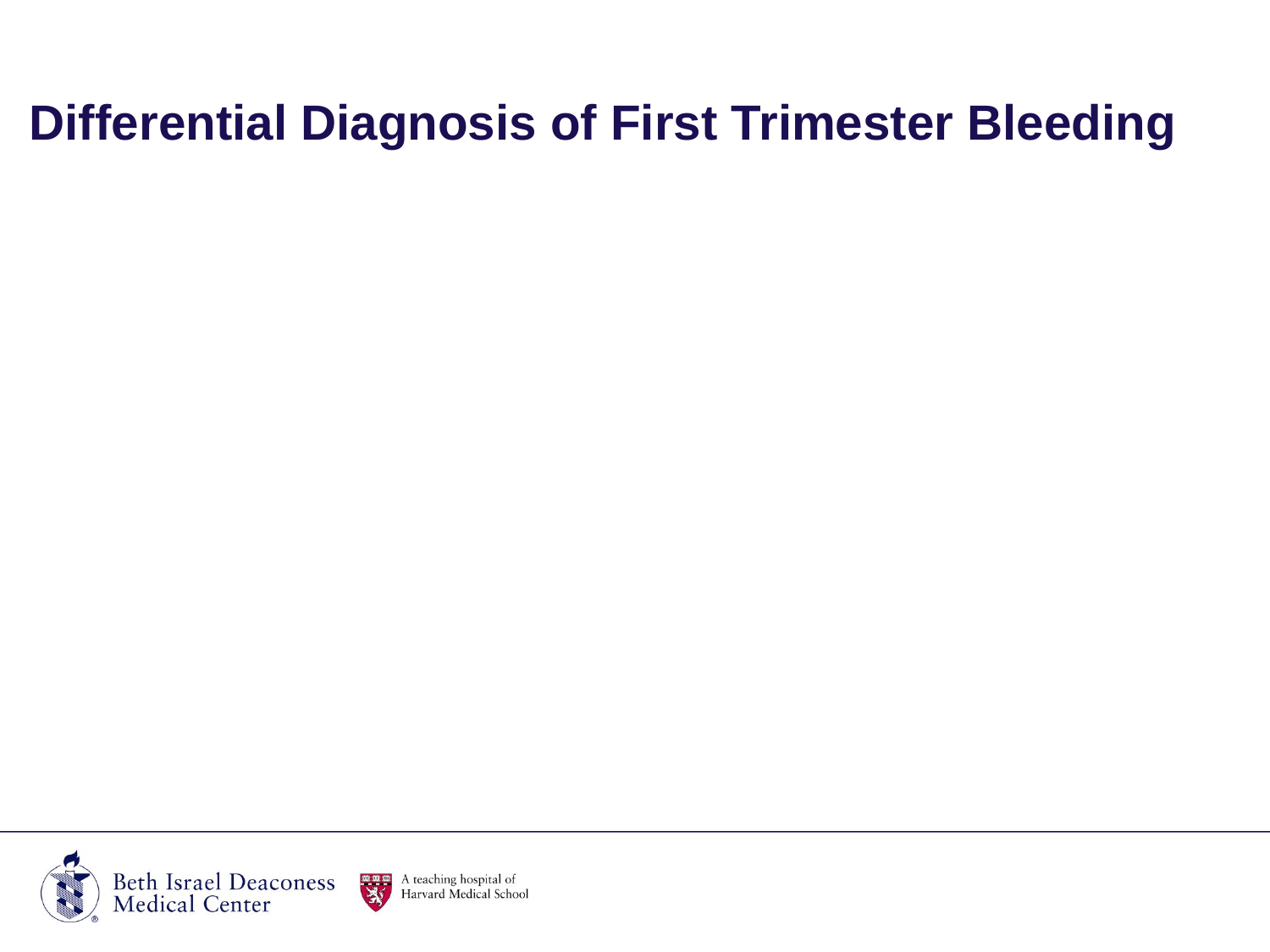

Differential Diagnosis of First Trimester Bleeding

## Slide 5
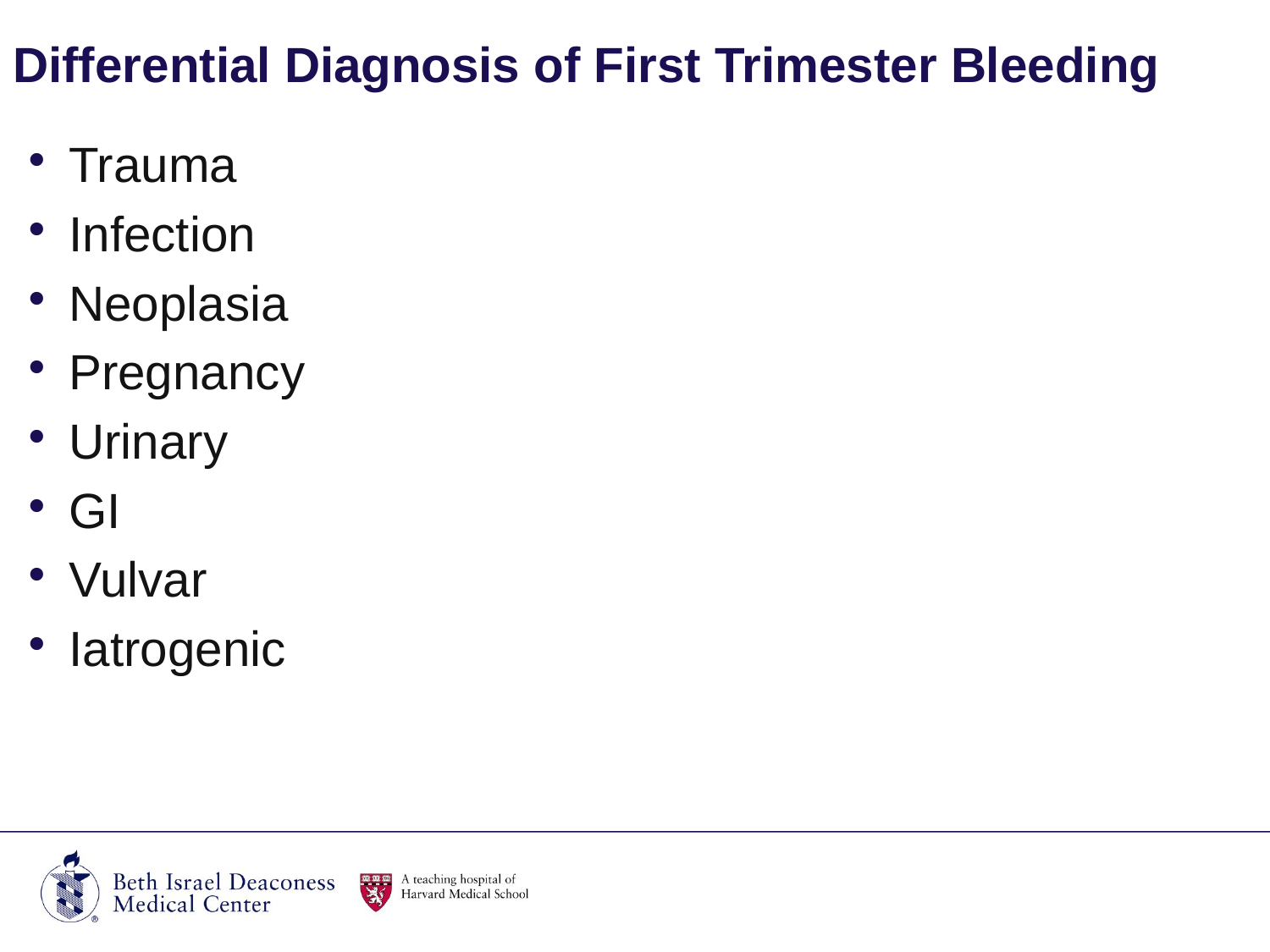

# Differential Diagnosis of First Trimester Bleeding
Trauma
Infection
Neoplasia
Pregnancy
Urinary
GI
Vulvar
Iatrogenic

## Slide 6
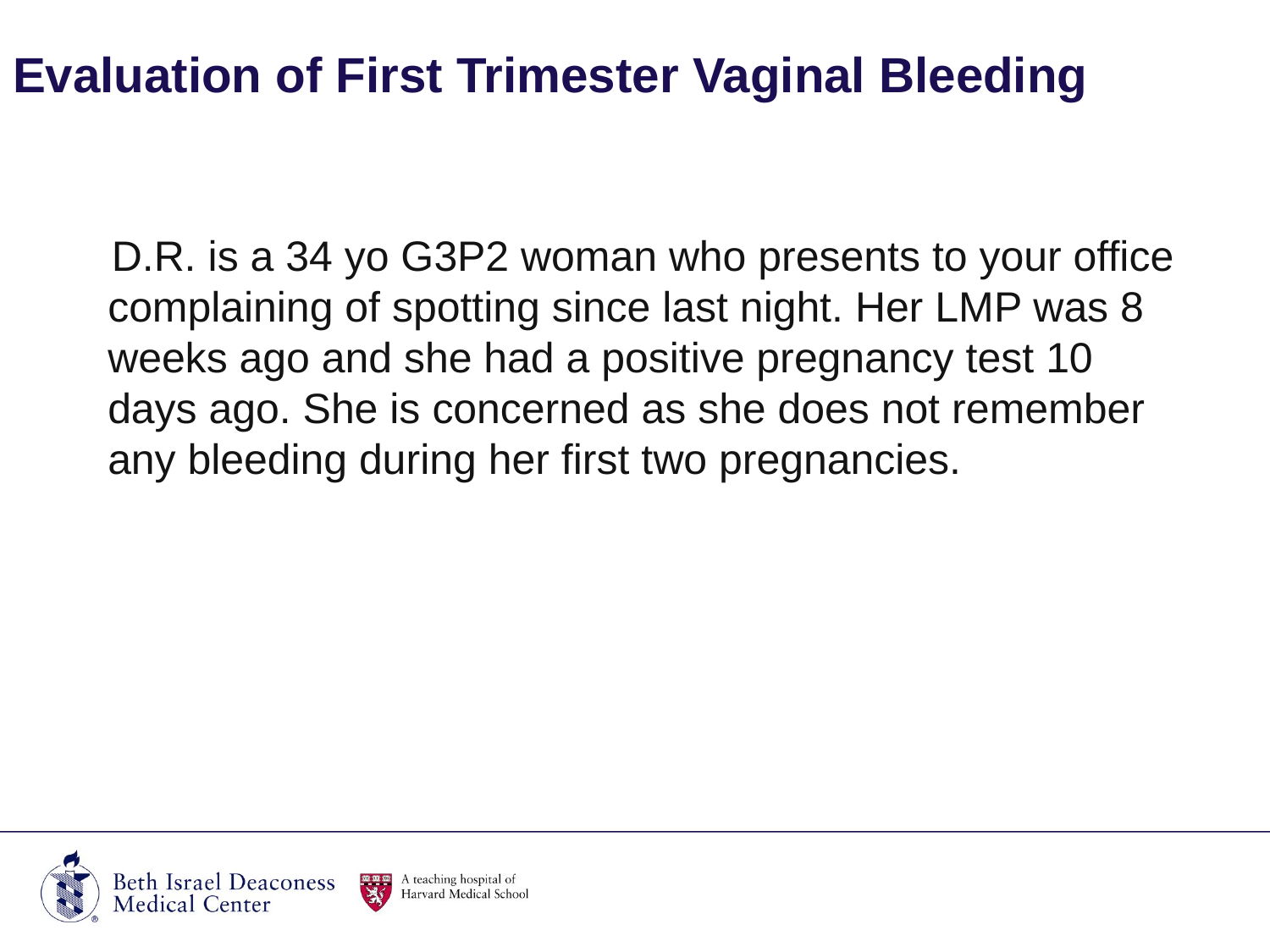

# Evaluation of First Trimester Vaginal Bleeding
 D.R. is a 34 yo G3P2 woman who presents to your office complaining of spotting since last night. Her LMP was 8 weeks ago and she had a positive pregnancy test 10 days ago. She is concerned as she does not remember any bleeding during her first two pregnancies.

## Slide 7
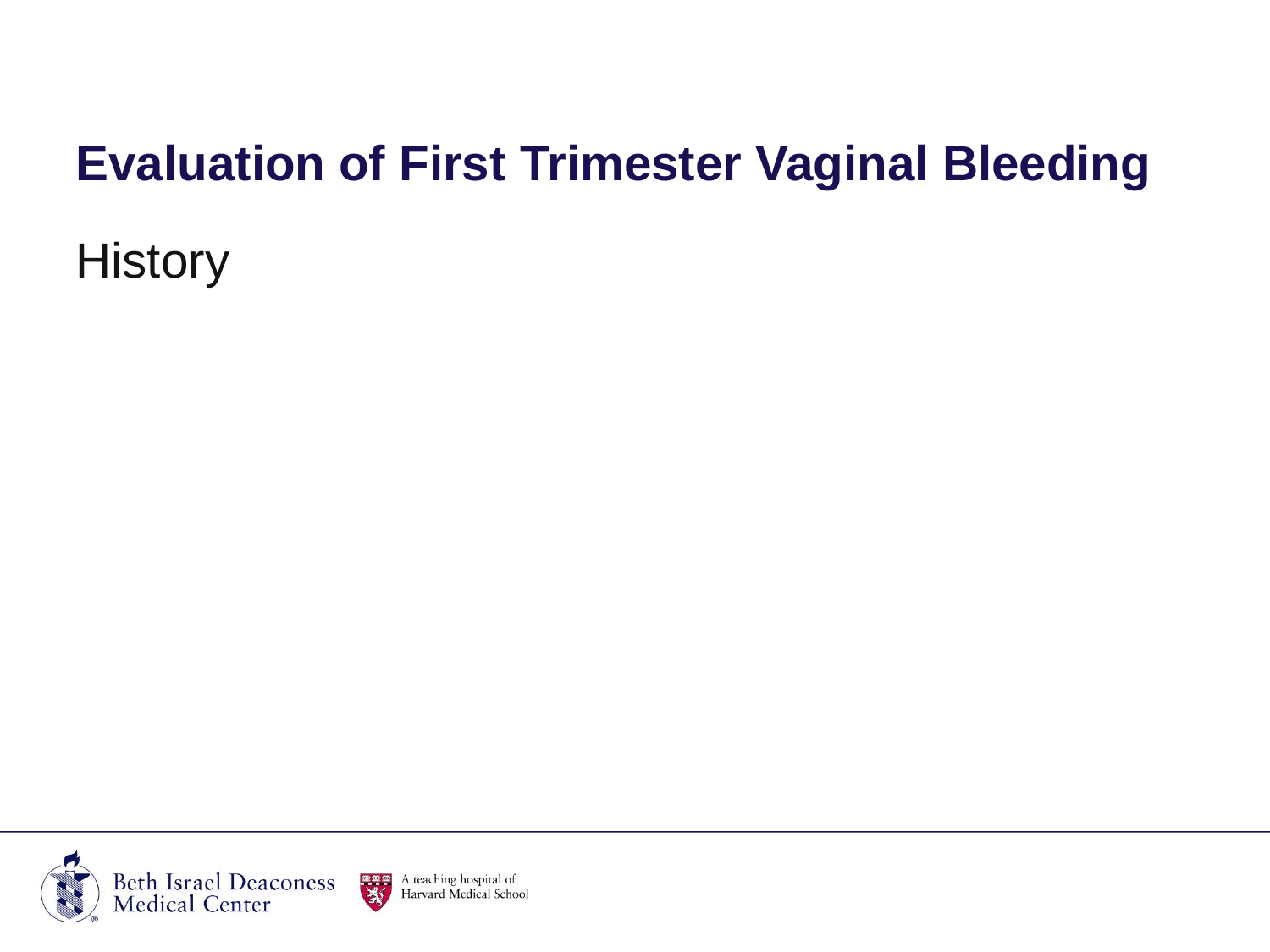

# Evaluation of First Trimester Vaginal Bleeding
History

## Slide 8
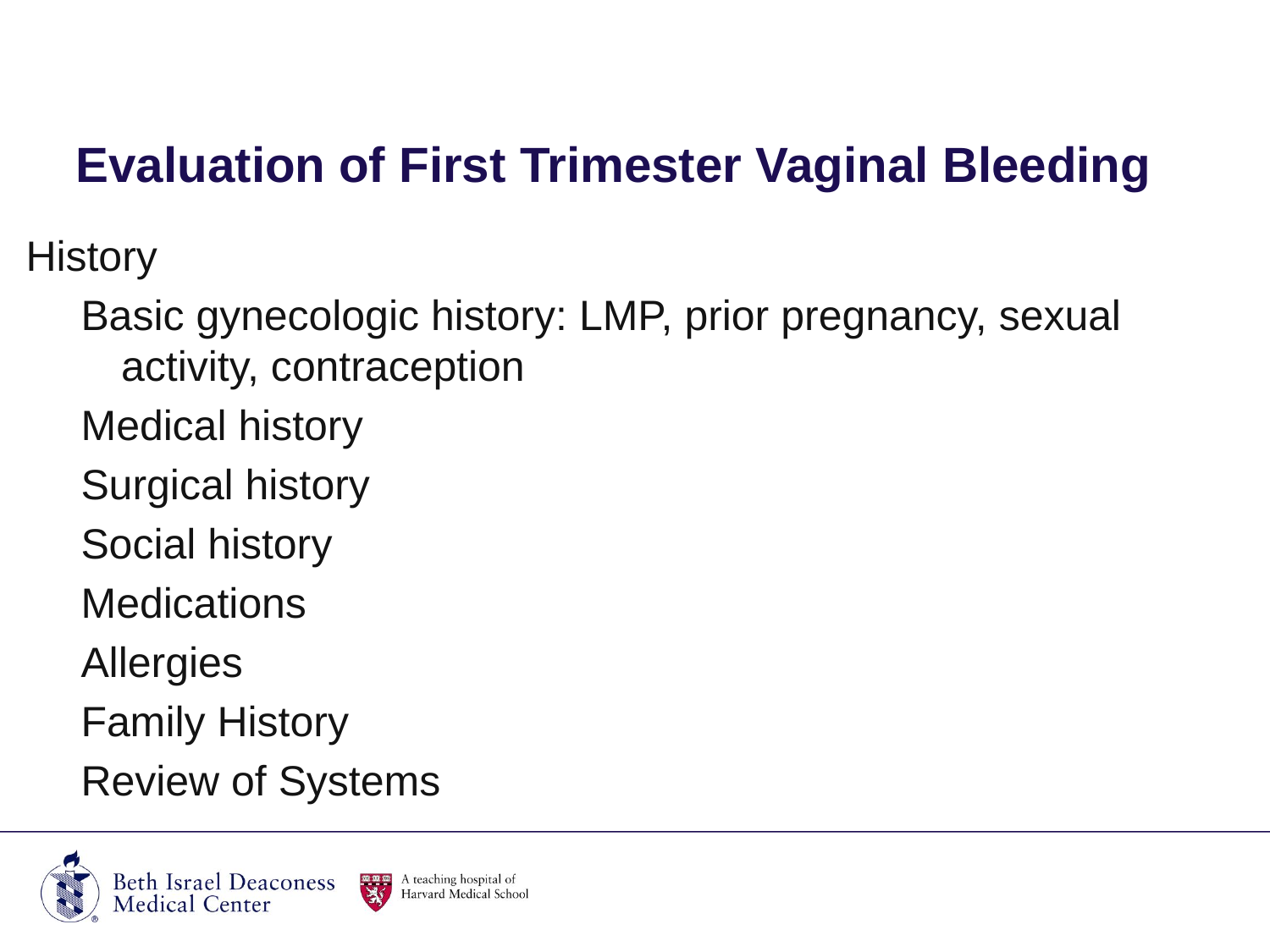

# Evaluation of First Trimester Vaginal Bleeding
History
Basic gynecologic history: LMP, prior pregnancy, sexual activity, contraception
Medical history
Surgical history
Social history
Medications
Allergies
Family History
Review of Systems

## Slide 9
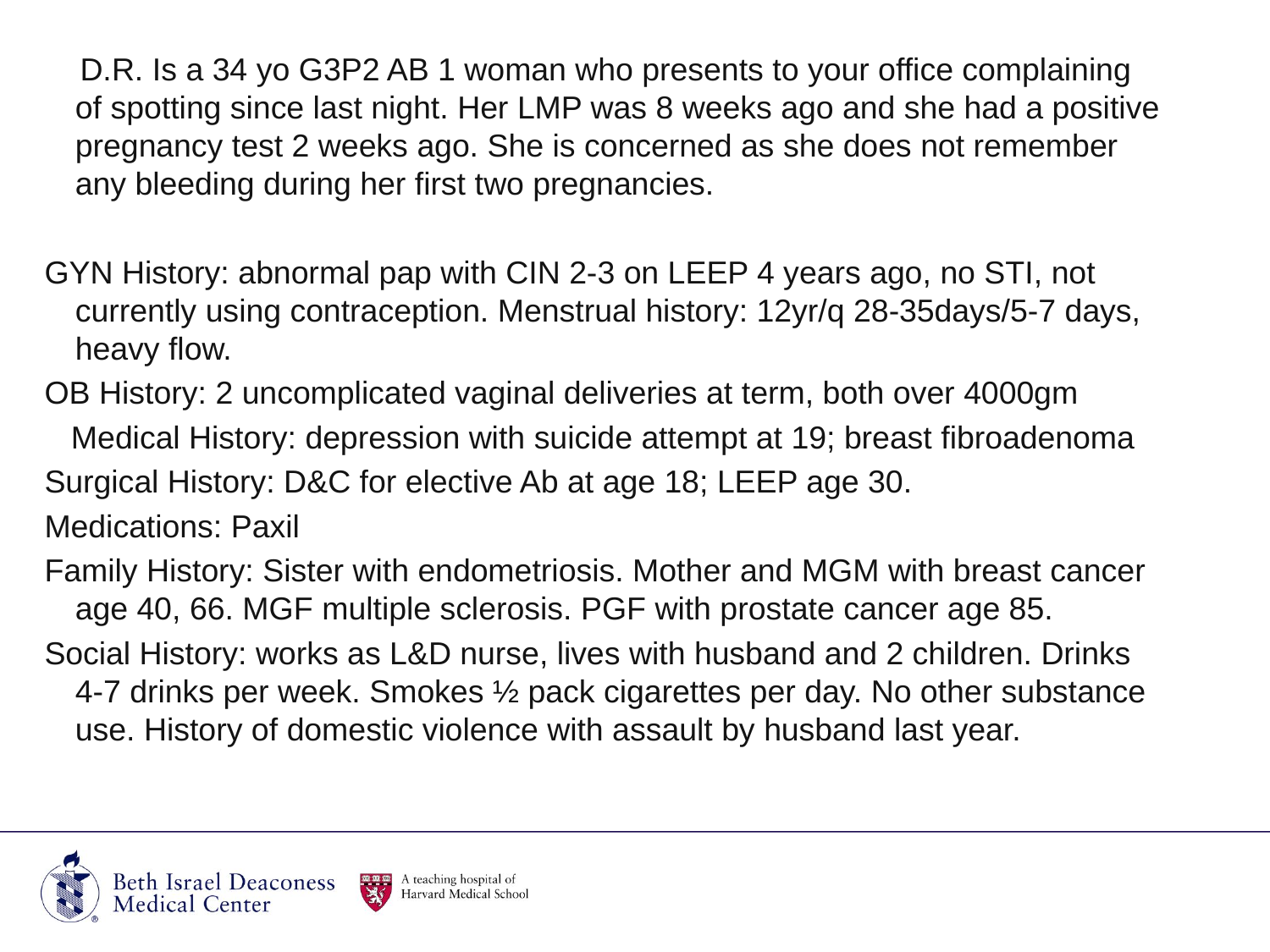

D.R. Is a 34 yo G3P2 AB 1 woman who presents to your office complaining of spotting since last night. Her LMP was 8 weeks ago and she had a positive pregnancy test 2 weeks ago. She is concerned as she does not remember any bleeding during her first two pregnancies.
GYN History: abnormal pap with CIN 2-3 on LEEP 4 years ago, no STI, not currently using contraception. Menstrual history: 12yr/q 28-35days/5-7 days, heavy flow.
OB History: 2 uncomplicated vaginal deliveries at term, both over 4000gm
 Medical History: depression with suicide attempt at 19; breast fibroadenoma
Surgical History: D&C for elective Ab at age 18; LEEP age 30.
Medications: Paxil
Family History: Sister with endometriosis. Mother and MGM with breast cancer age 40, 66. MGF multiple sclerosis. PGF with prostate cancer age 85.
Social History: works as L&D nurse, lives with husband and 2 children. Drinks 4-7 drinks per week. Smokes ½ pack cigarettes per day. No other substance use. History of domestic violence with assault by husband last year.

## Slide 10
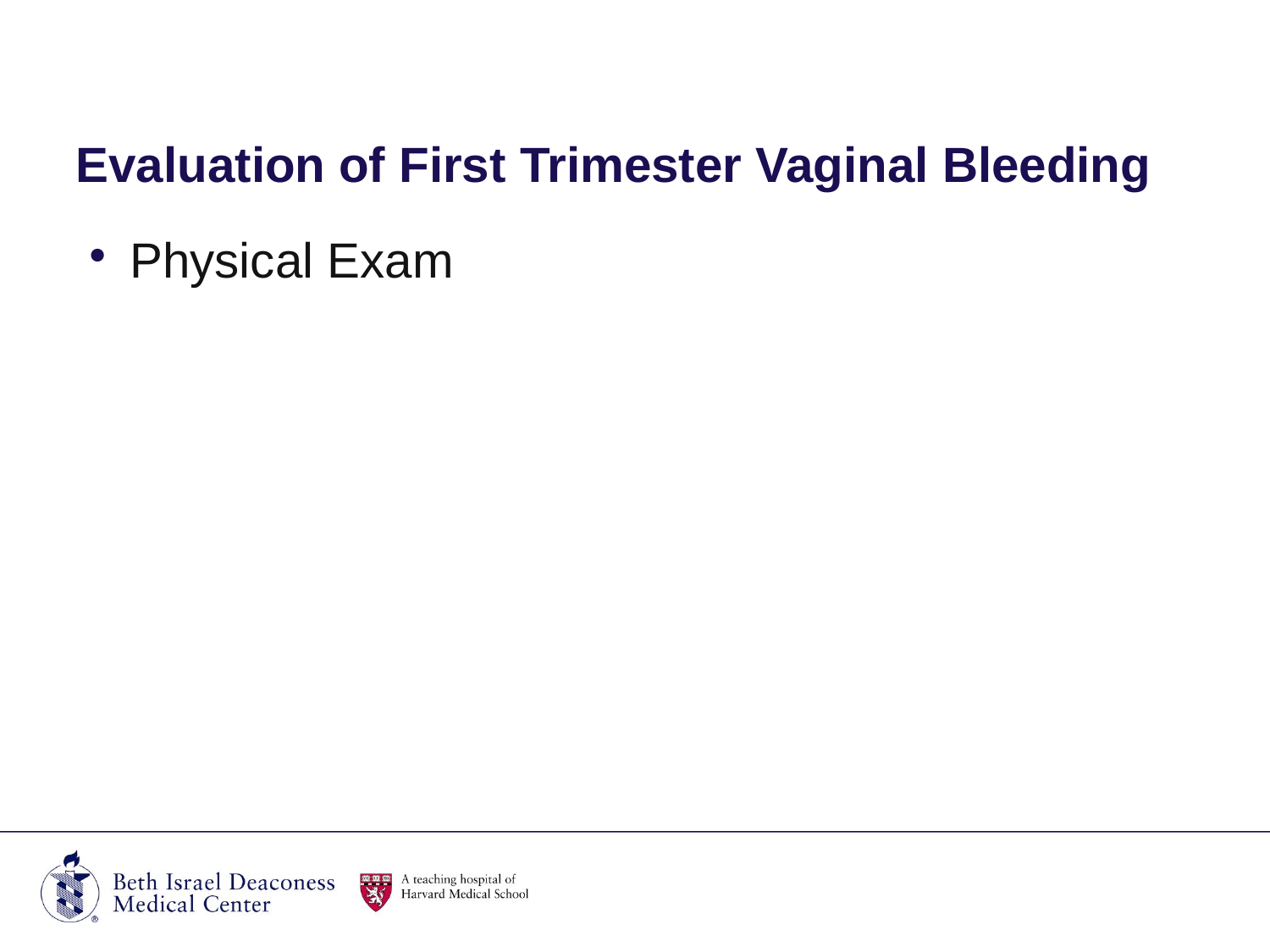

# Evaluation of First Trimester Vaginal Bleeding
Physical Exam

## Slide 11
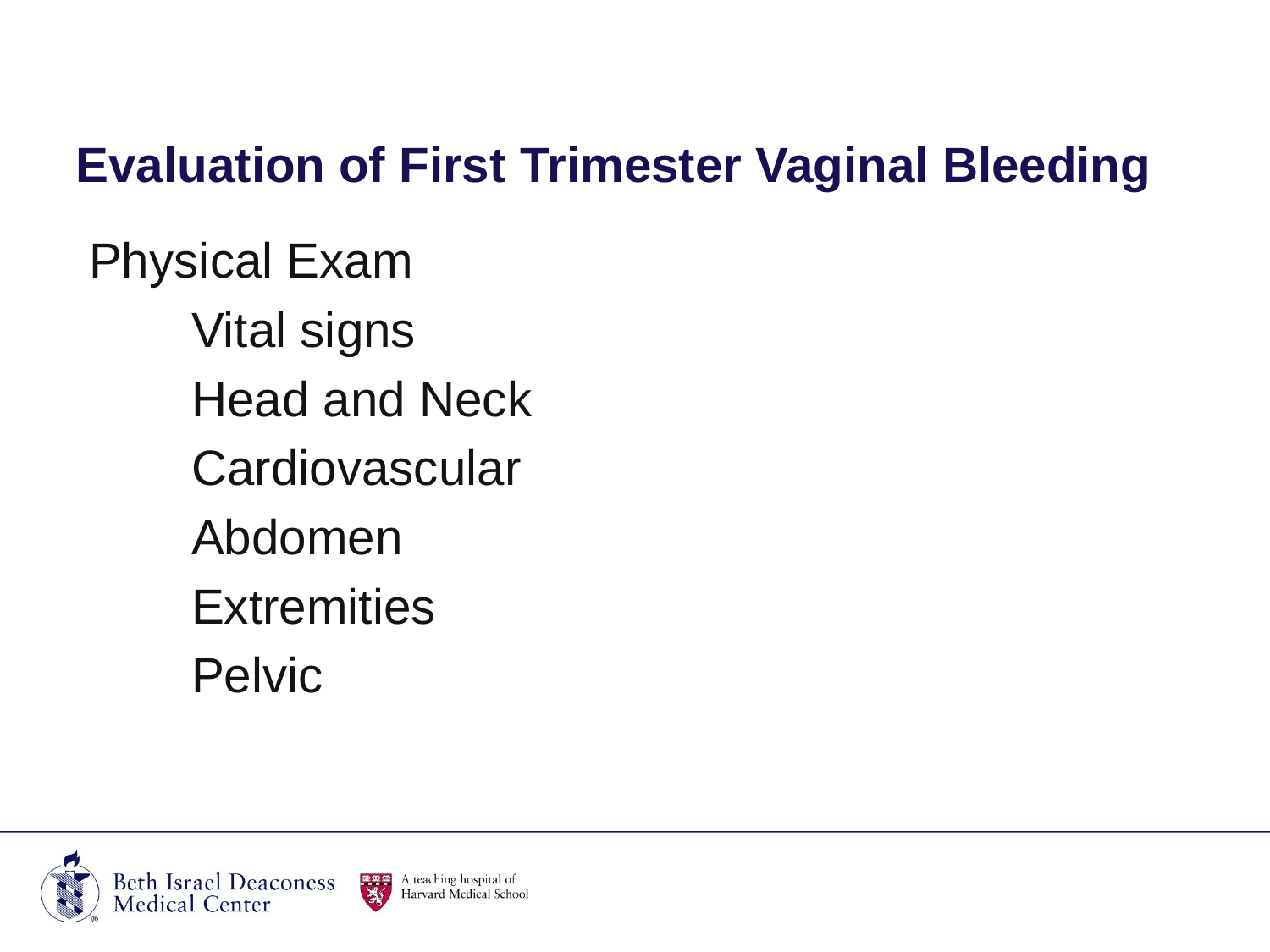

# Evaluation of First Trimester Vaginal Bleeding
Physical Exam
Vital signs
Head and Neck
Cardiovascular
Abdomen
Extremities
Pelvic

## Slide 12
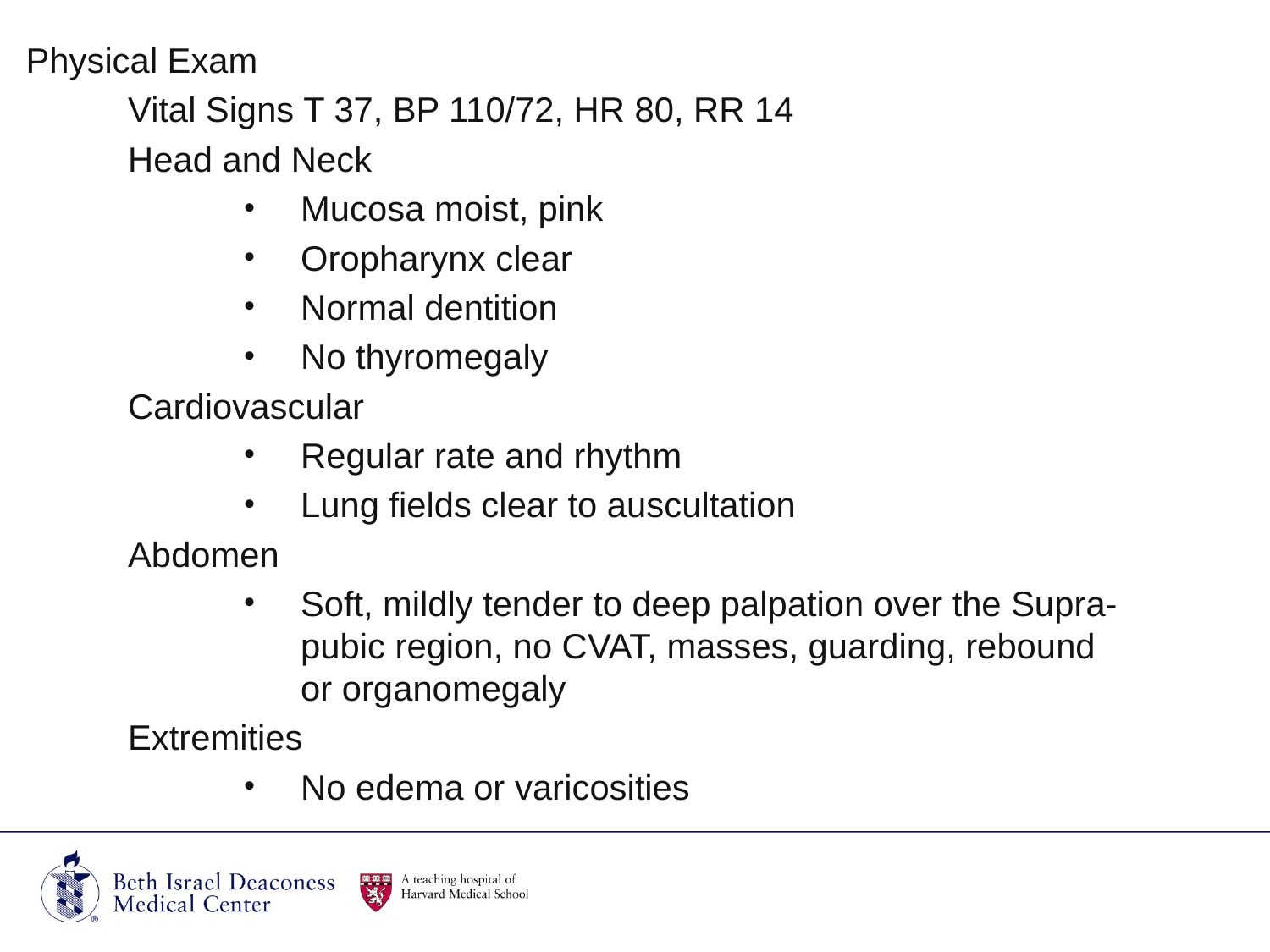

Physical Exam
Vital Signs T 37, BP 110/72, HR 80, RR 14
Head and Neck
Mucosa moist, pink
Oropharynx clear
Normal dentition
No thyromegaly
Cardiovascular
Regular rate and rhythm
Lung fields clear to auscultation
Abdomen
Soft, mildly tender to deep palpation over the Supra-pubic region, no CVAT, masses, guarding, rebound or organomegaly
Extremities
No edema or varicosities

## Slide 13
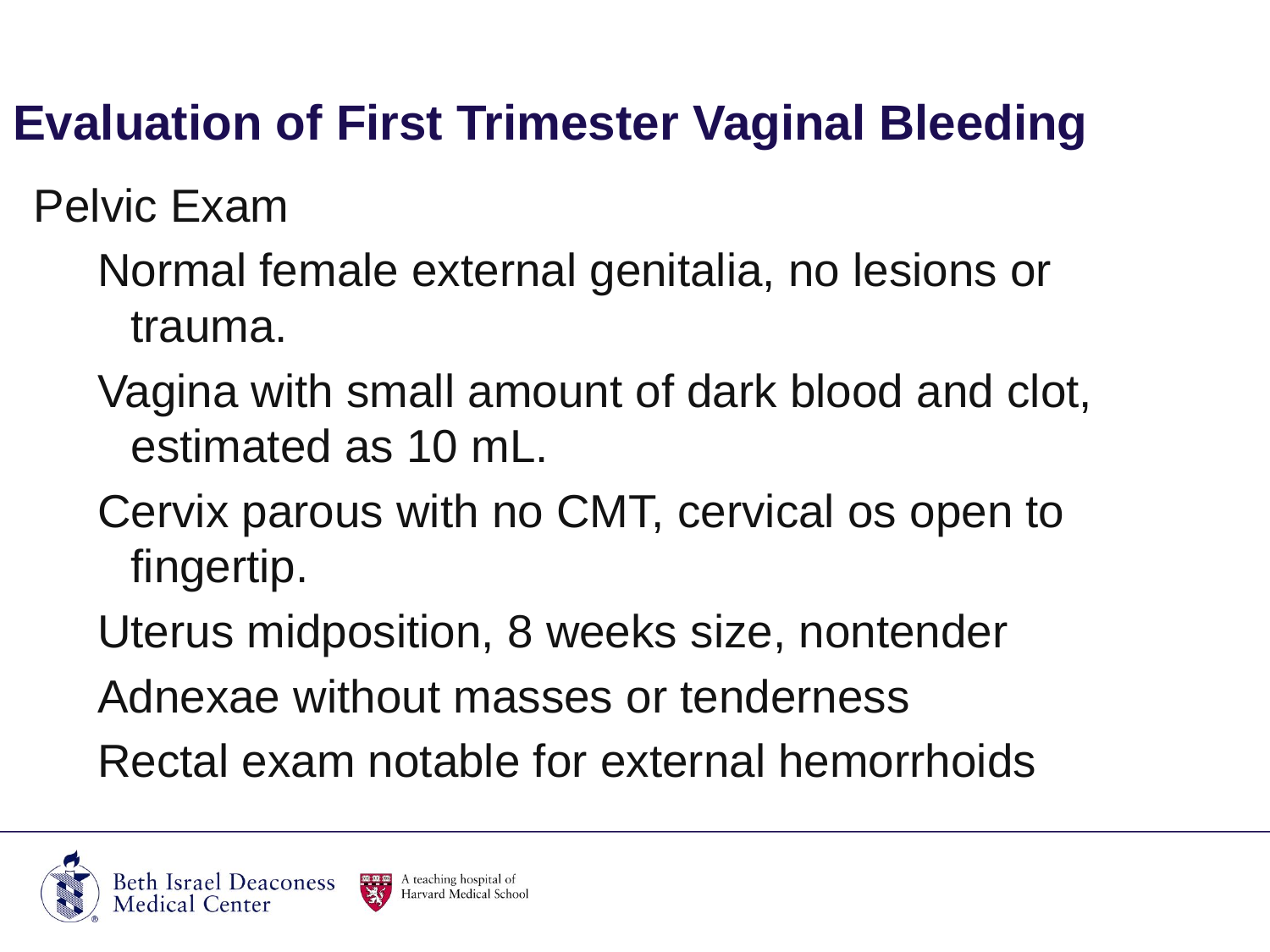

# Evaluation of First Trimester Vaginal Bleeding
Pelvic Exam
Normal female external genitalia, no lesions or trauma.
Vagina with small amount of dark blood and clot, estimated as 10 mL.
Cervix parous with no CMT, cervical os open to fingertip.
Uterus midposition, 8 weeks size, nontender
Adnexae without masses or tenderness
Rectal exam notable for external hemorrhoids

## Slide 14
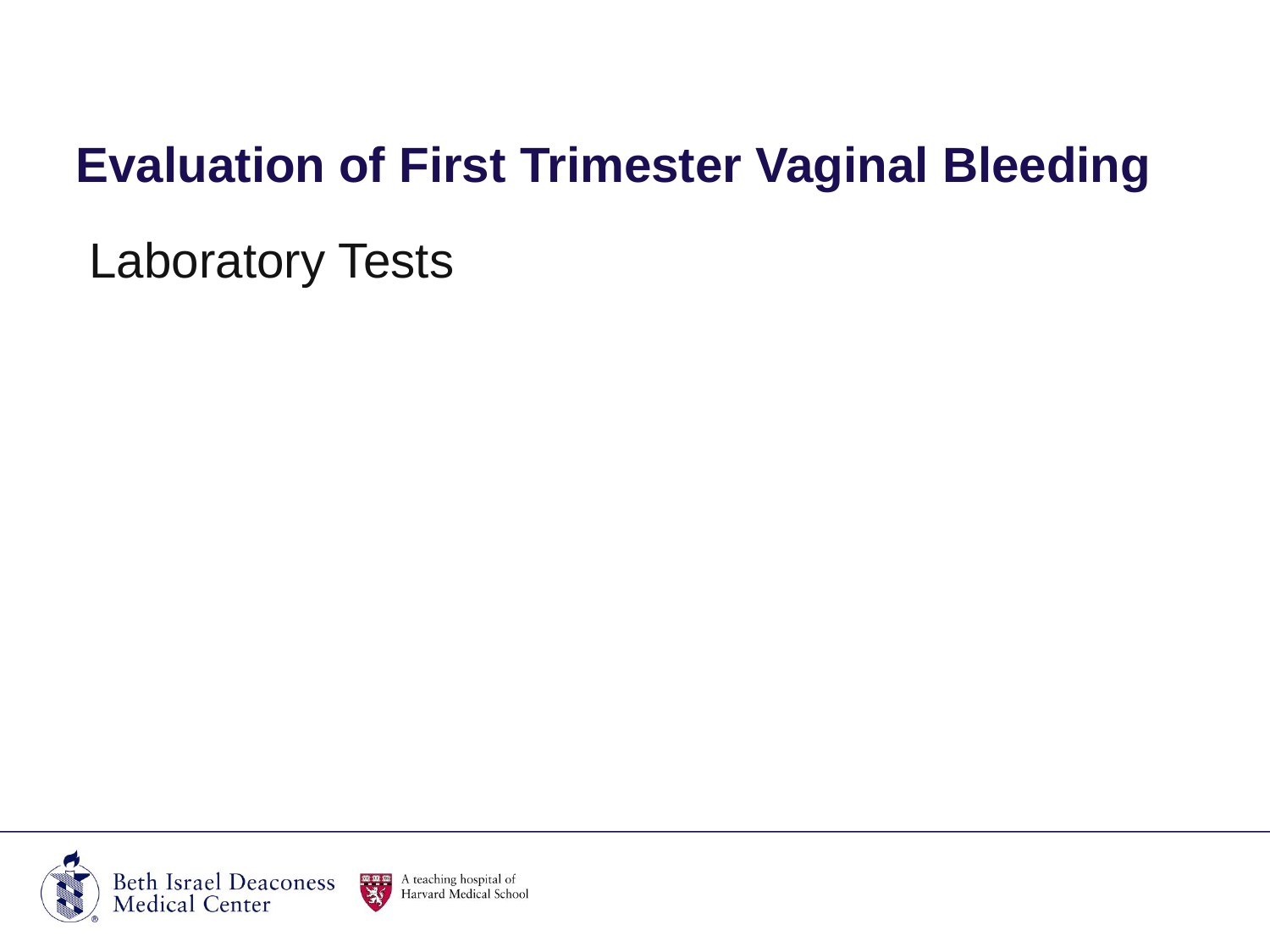

# Evaluation of First Trimester Vaginal Bleeding
Laboratory Tests

## Slide 15
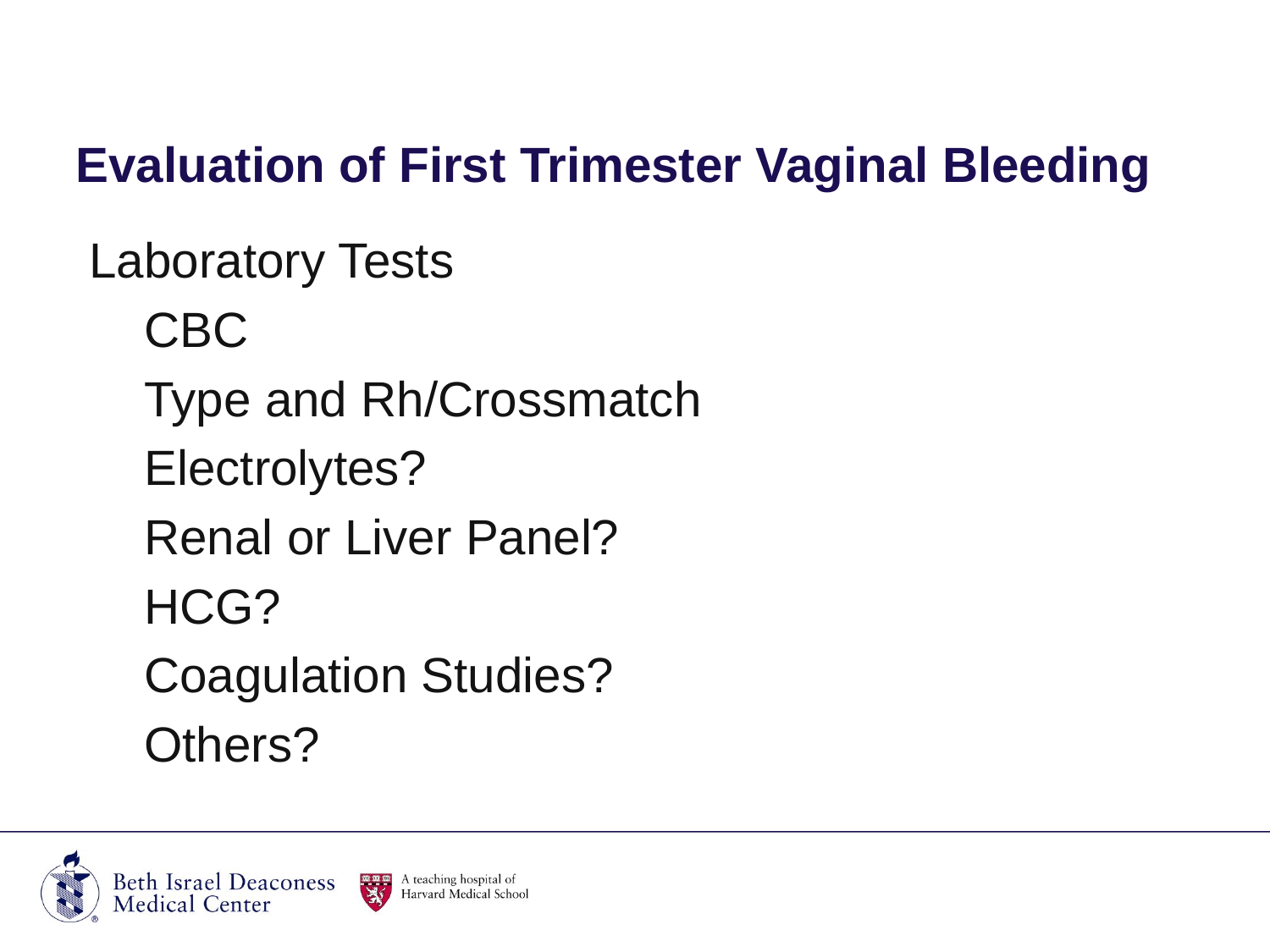

# Evaluation of First Trimester Vaginal Bleeding
Laboratory Tests
CBC
Type and Rh/Crossmatch
Electrolytes?
Renal or Liver Panel?
HCG?
Coagulation Studies?
Others?

## Slide 16
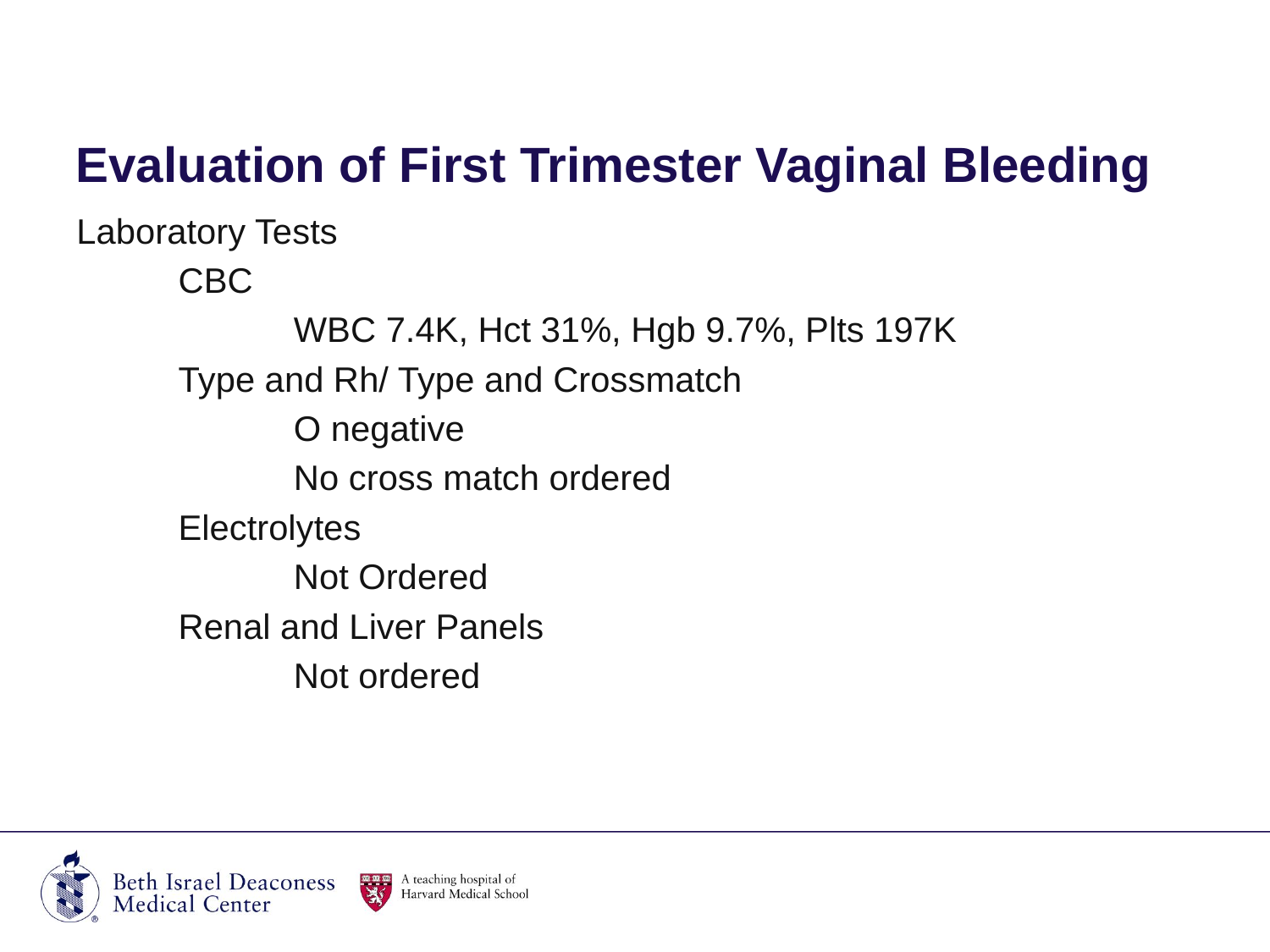

# Evaluation of First Trimester Vaginal Bleeding
Laboratory Tests
CBC
WBC 7.4K, Hct 31%, Hgb 9.7%, Plts 197K
Type and Rh/ Type and Crossmatch
O negative
No cross match ordered
Electrolytes
Not Ordered
Renal and Liver Panels
Not ordered

## Slide 17
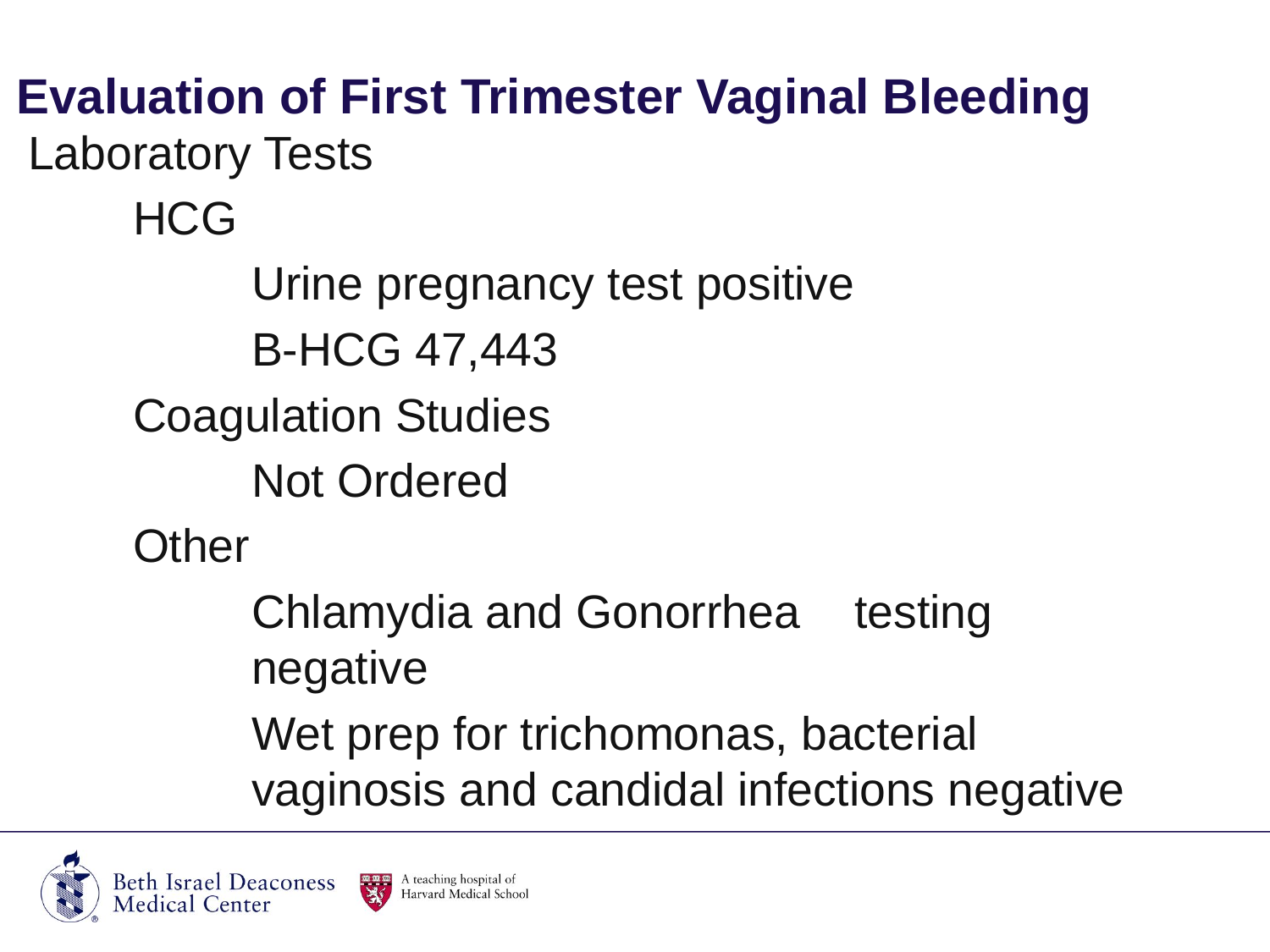

# Evaluation of First Trimester Vaginal Bleeding
Laboratory Tests
HCG
Urine pregnancy test positive
B-HCG 47,443
Coagulation Studies
Not Ordered
Other
Chlamydia and Gonorrhea	testing negative
Wet prep for trichomonas, bacterial vaginosis and candidal infections negative

## Slide 18
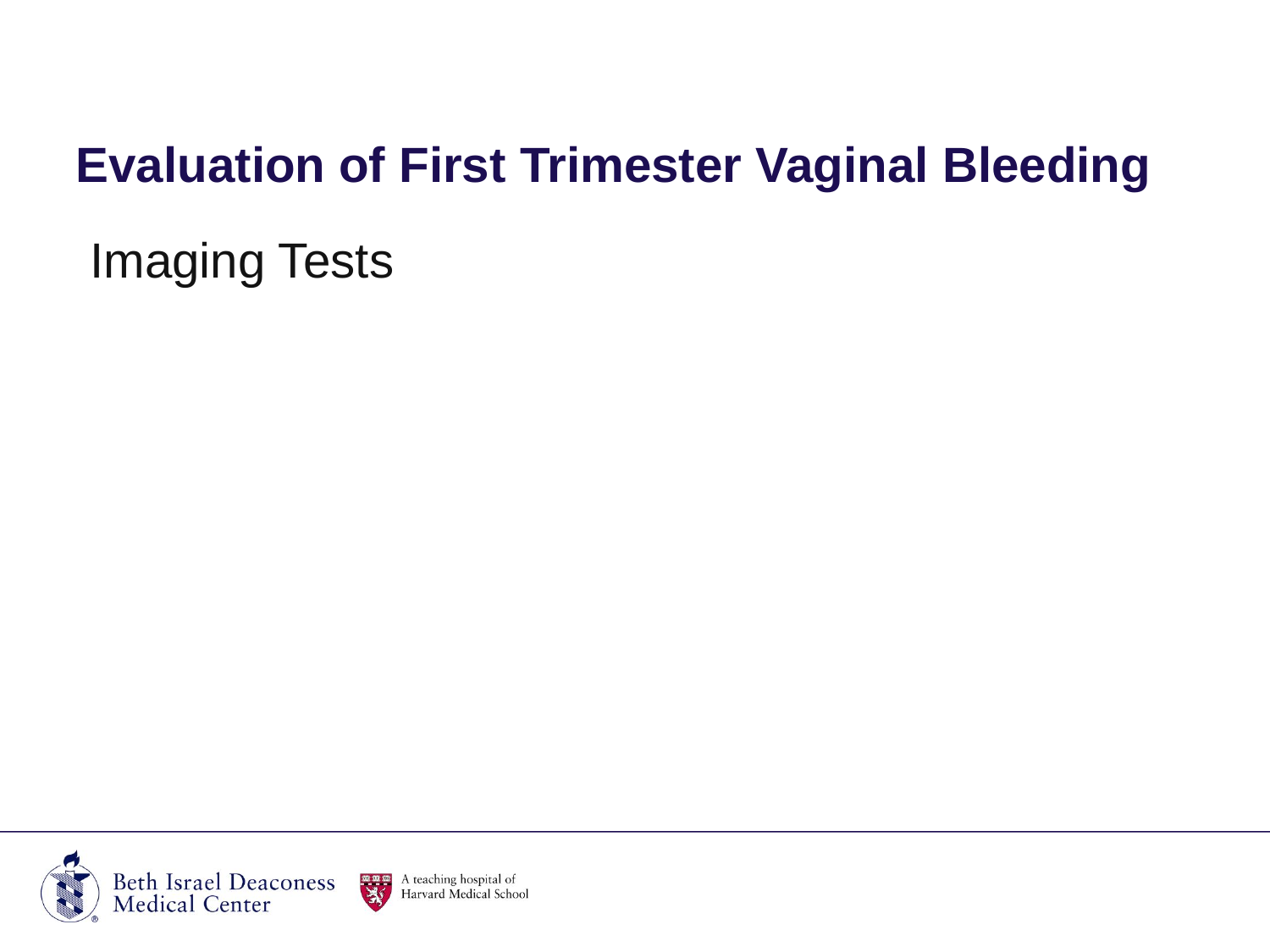

# Evaluation of First Trimester Vaginal Bleeding
Imaging Tests

## Slide 19
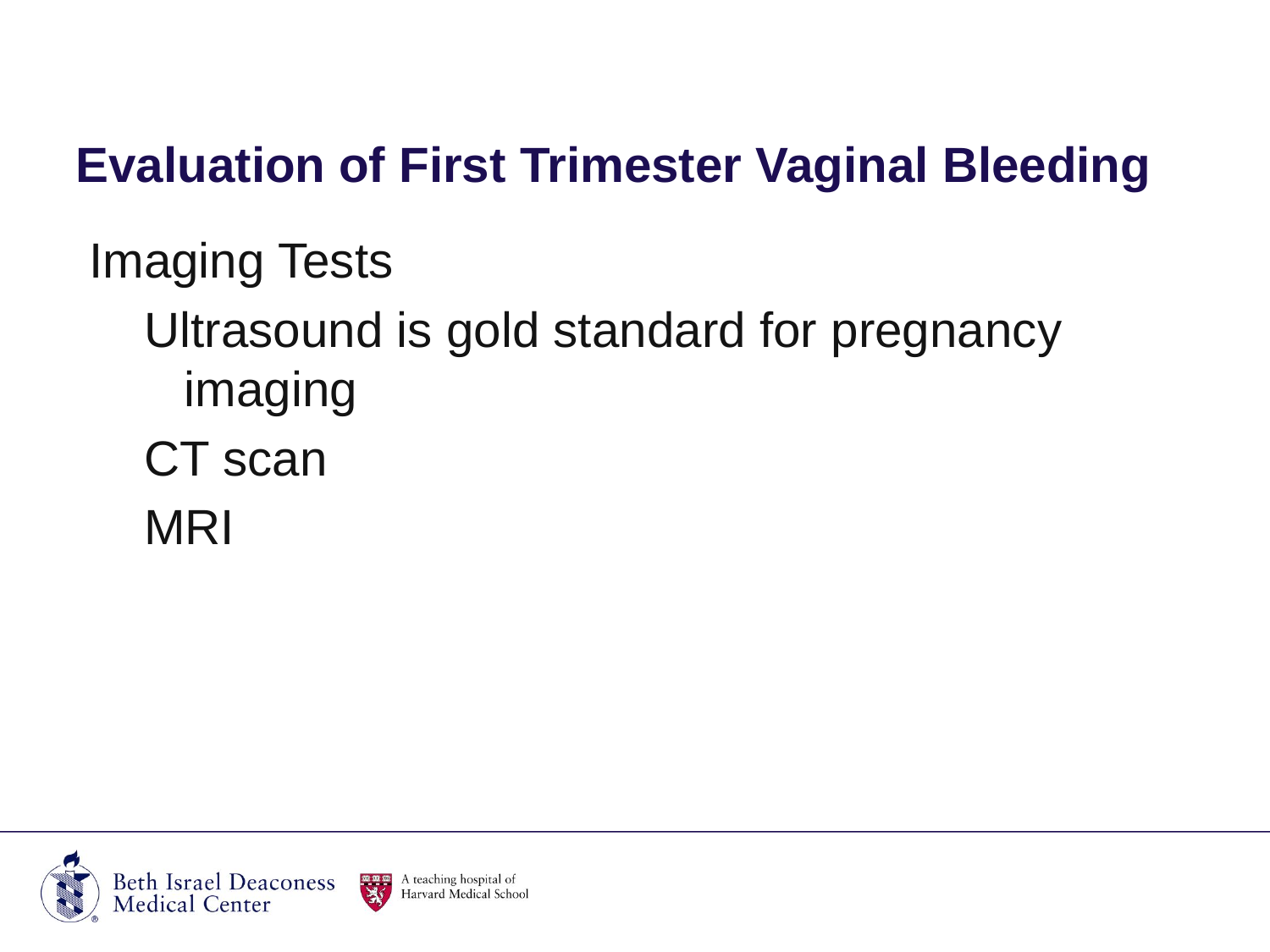

# Evaluation of First Trimester Vaginal Bleeding
Imaging Tests
Ultrasound is gold standard for pregnancy imaging
CT scan
MRI

## Slide 20
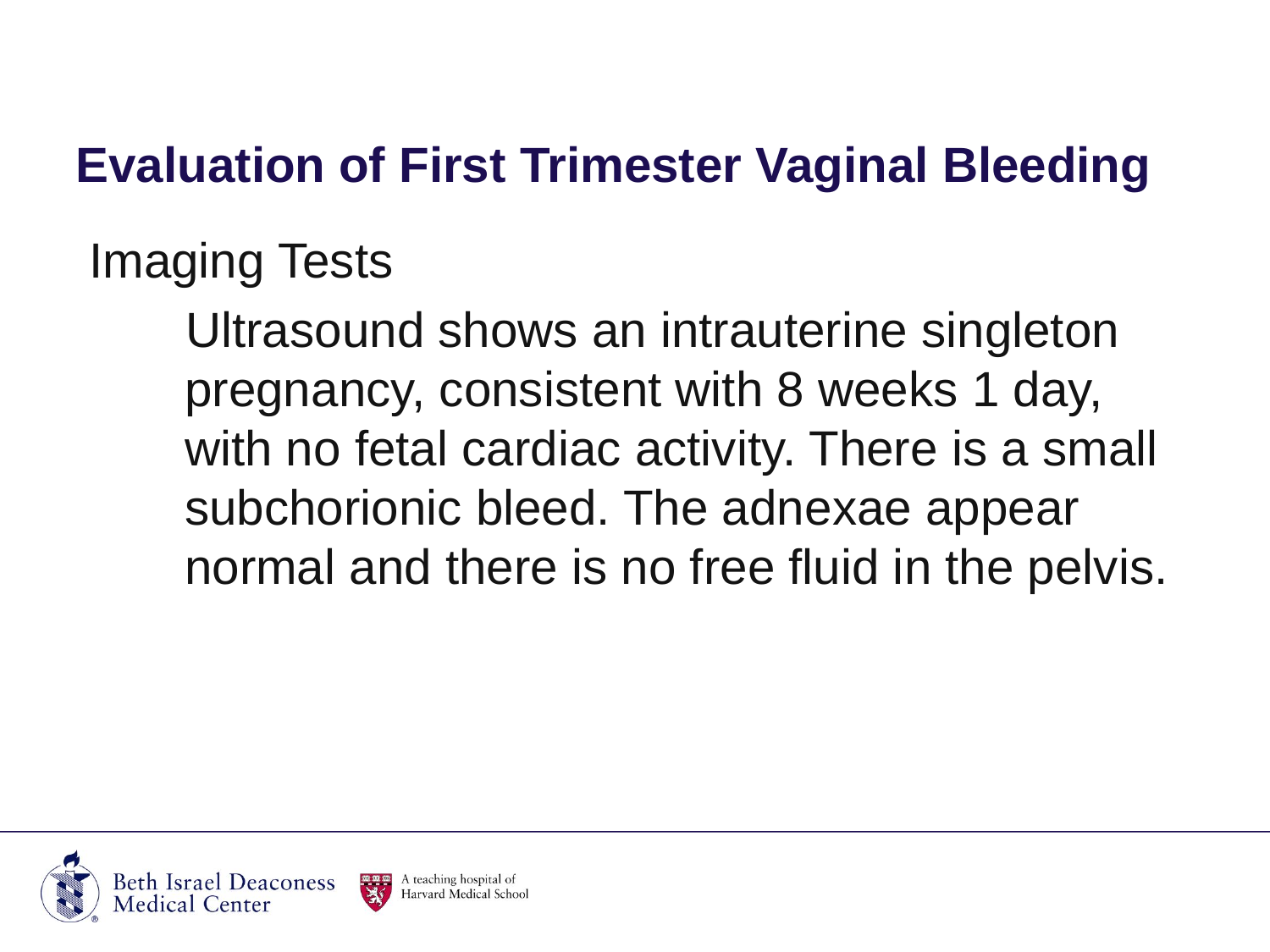

# Evaluation of First Trimester Vaginal Bleeding
Imaging Tests
 Ultrasound shows an intrauterine singleton pregnancy, consistent with 8 weeks 1 day, with no fetal cardiac activity. There is a small subchorionic bleed. The adnexae appear normal and there is no free fluid in the pelvis.

## Slide 21
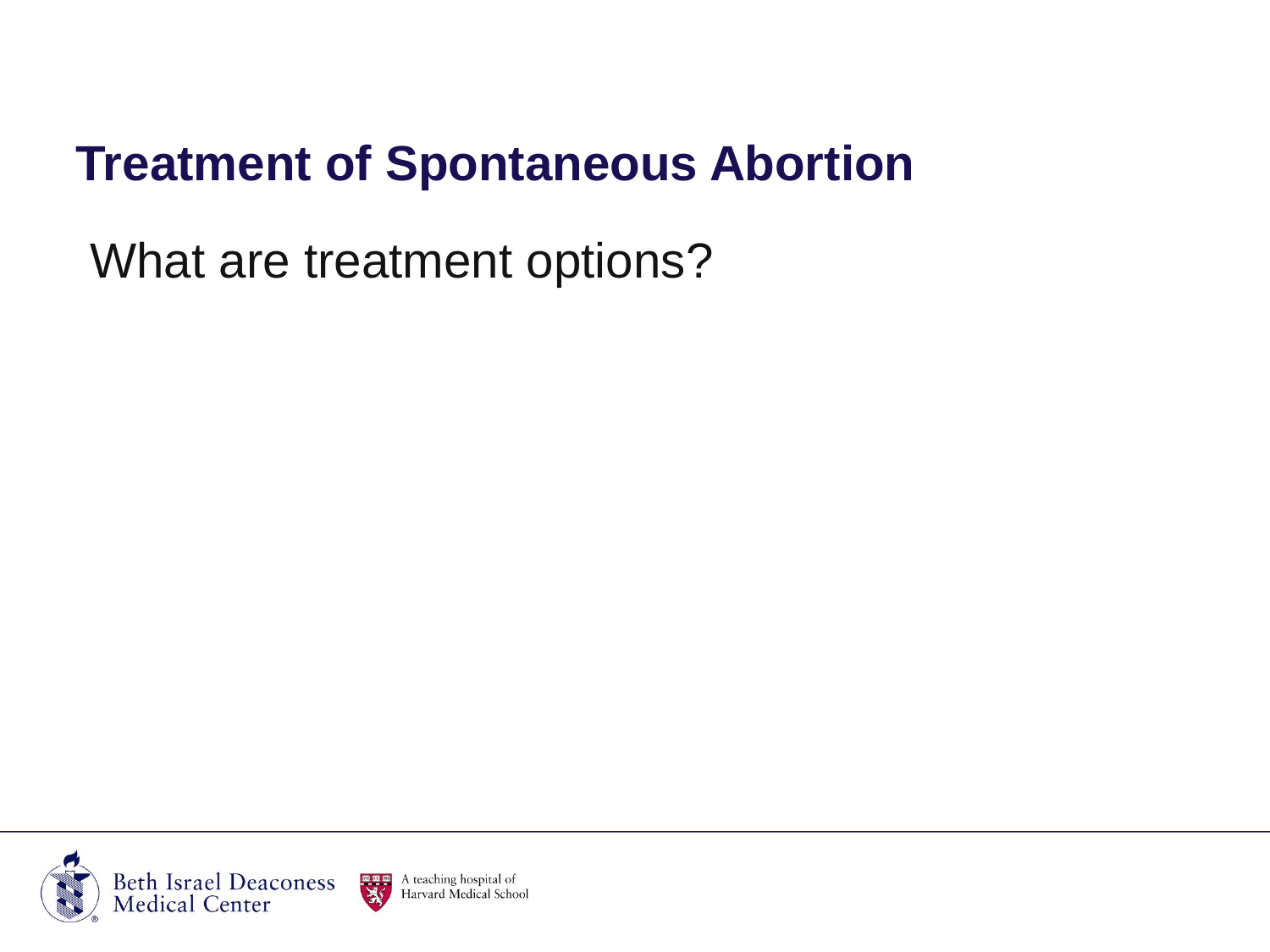

# Treatment of Spontaneous Abortion
What are treatment options?

## Slide 22
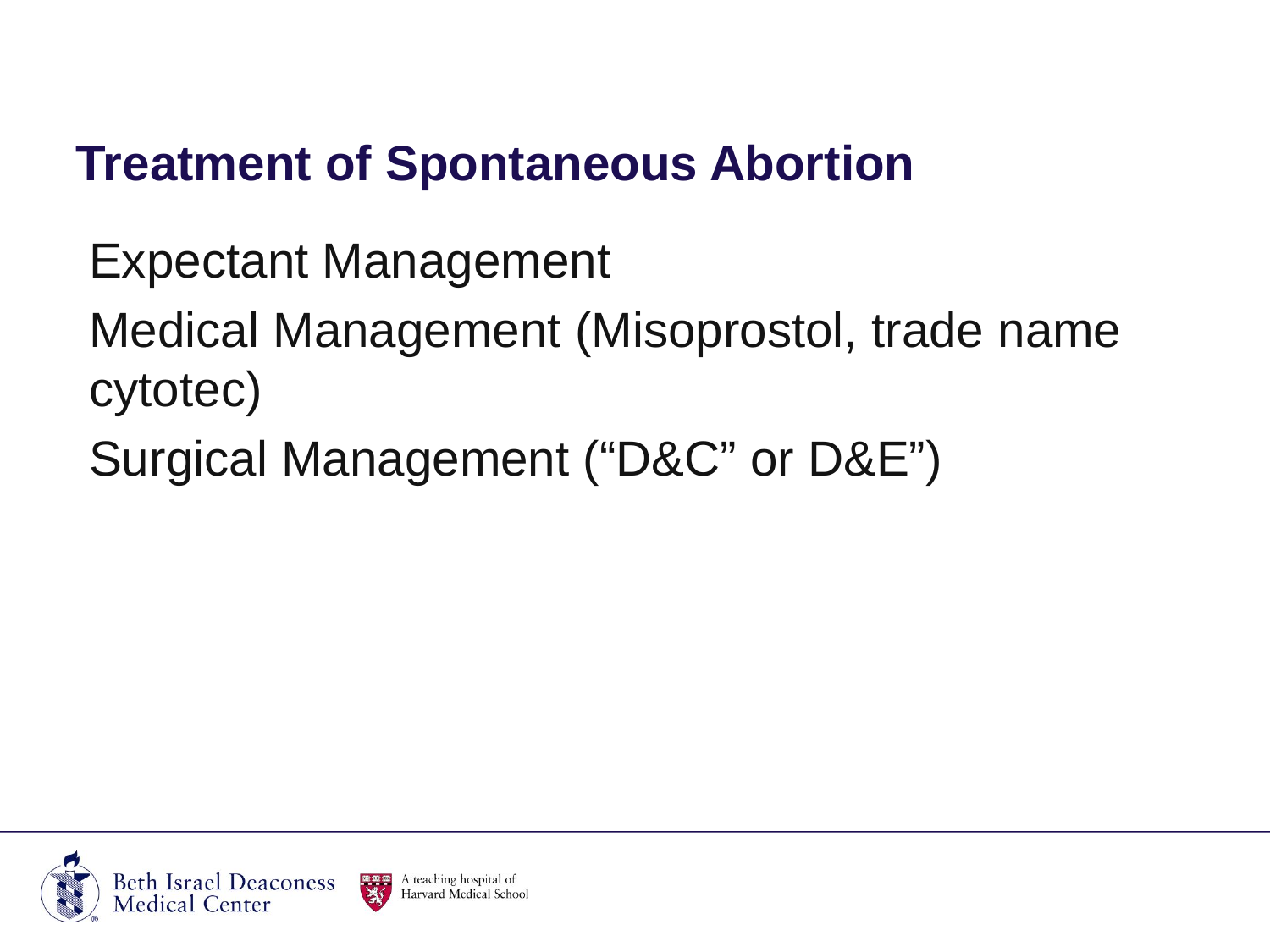

# Treatment of Spontaneous Abortion
Expectant Management
Medical Management (Misoprostol, trade name cytotec)
Surgical Management (“D&C” or D&E”)

## Slide 23
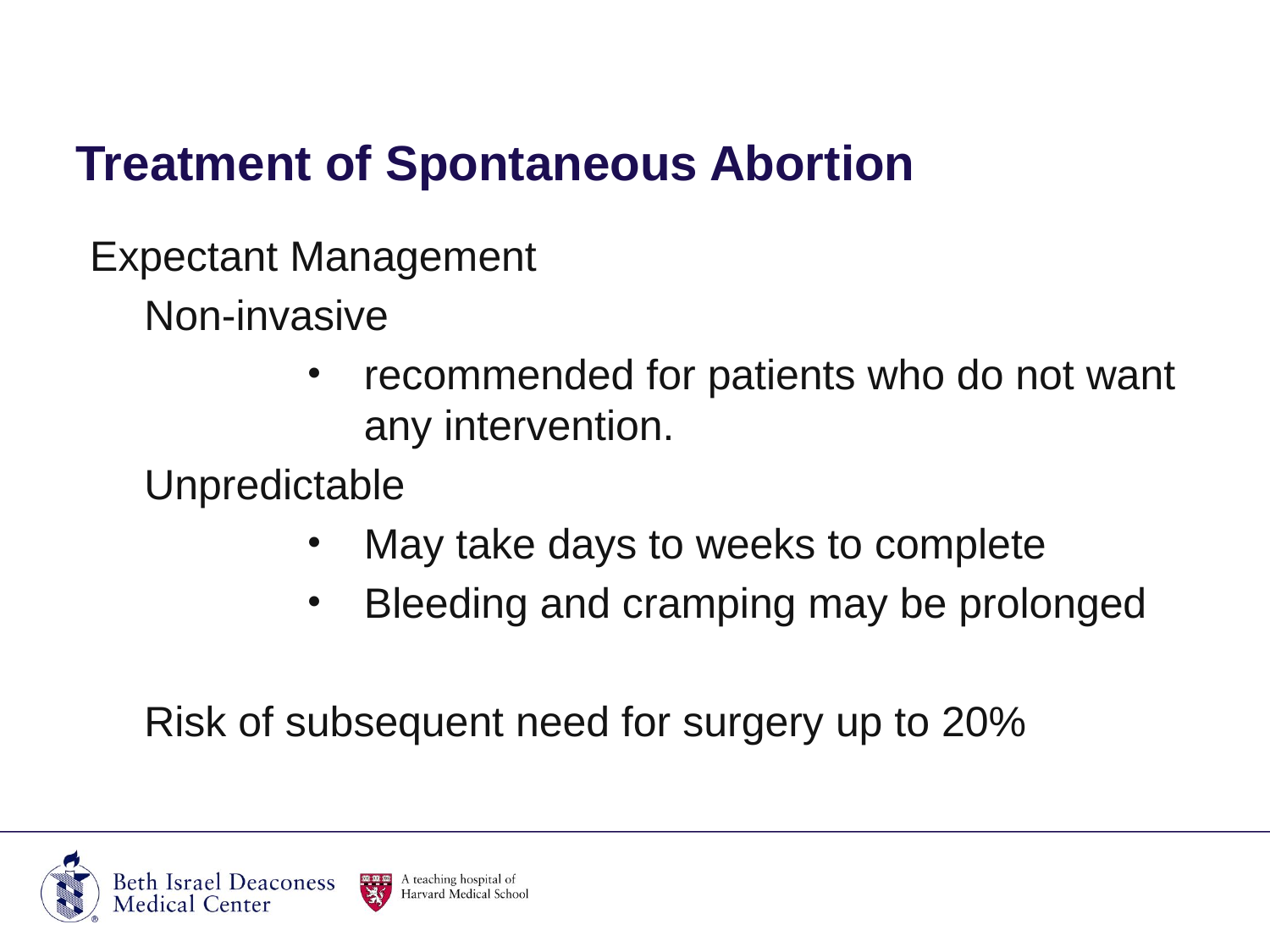

# Treatment of Spontaneous Abortion
Expectant Management
Non-invasive
recommended for patients who do not want any intervention.
Unpredictable
May take days to weeks to complete
Bleeding and cramping may be prolonged
Risk of subsequent need for surgery up to 20%

## Slide 24
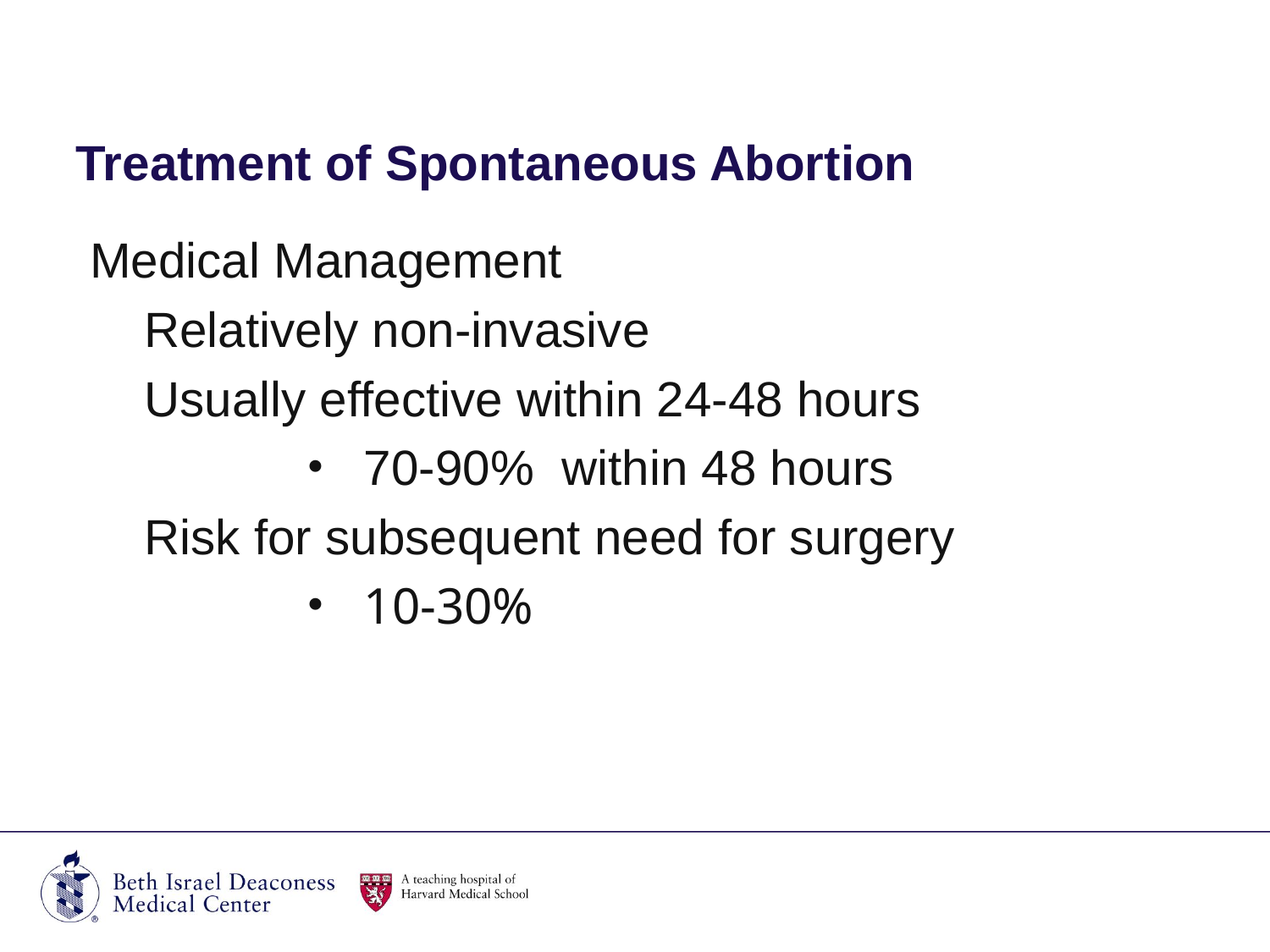

# Treatment of Spontaneous Abortion
Medical Management
Relatively non-invasive
Usually effective within 24-48 hours
70-90% within 48 hours
Risk for subsequent need for surgery
10-30%

## Slide 25
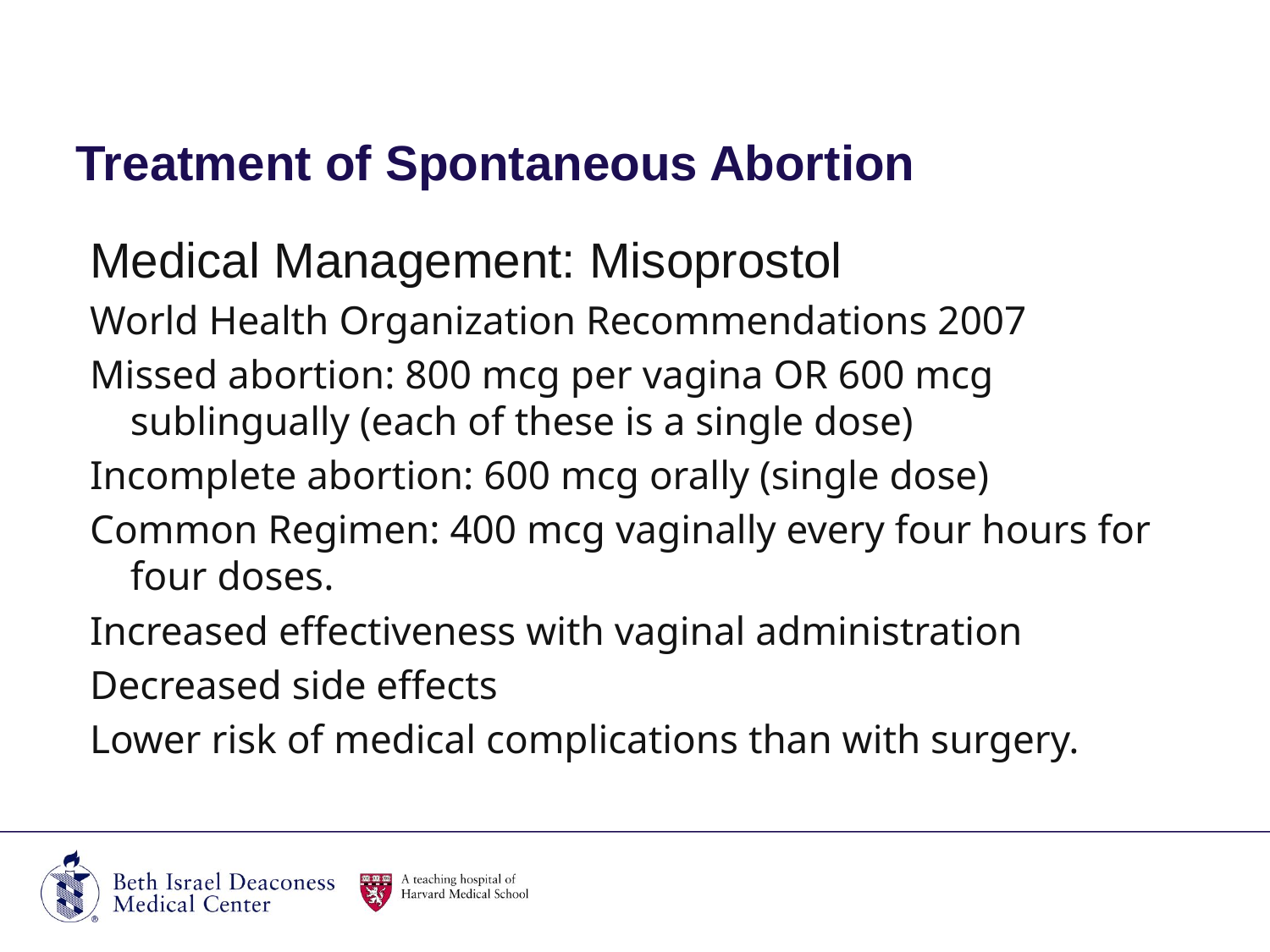

# Treatment of Spontaneous Abortion
Medical Management: Misoprostol
World Health Organization Recommendations 2007
Missed abortion: 800 mcg per vagina OR 600 mcg sublingually (each of these is a single dose)
Incomplete abortion: 600 mcg orally (single dose)
Common Regimen: 400 mcg vaginally every four hours for four doses.
Increased effectiveness with vaginal administration
Decreased side effects
Lower risk of medical complications than with surgery.

## Slide 26
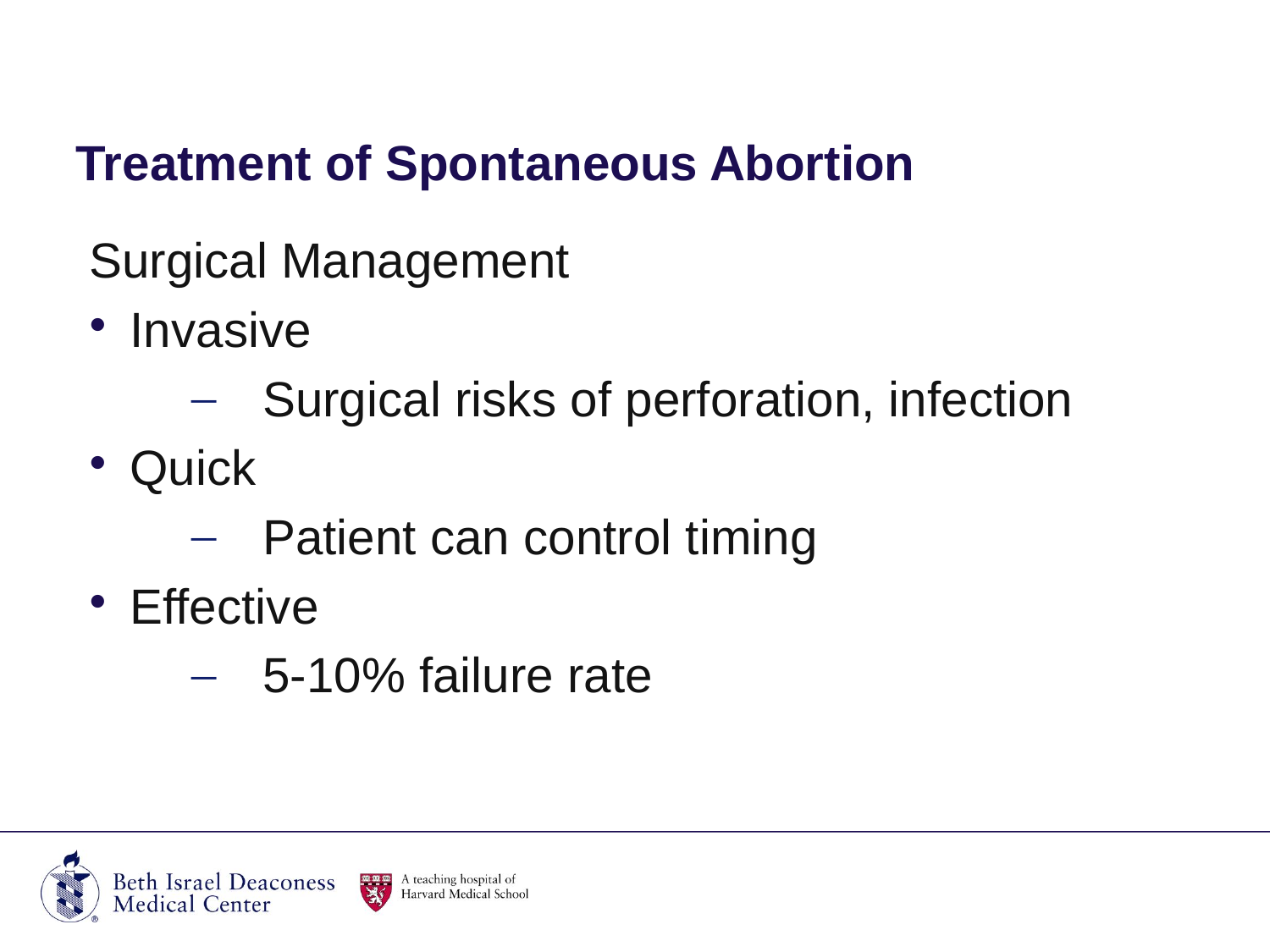

# Treatment of Spontaneous Abortion
Surgical Management
Invasive
Surgical risks of perforation, infection
Quick
Patient can control timing
Effective
5-10% failure rate

## Slide 27
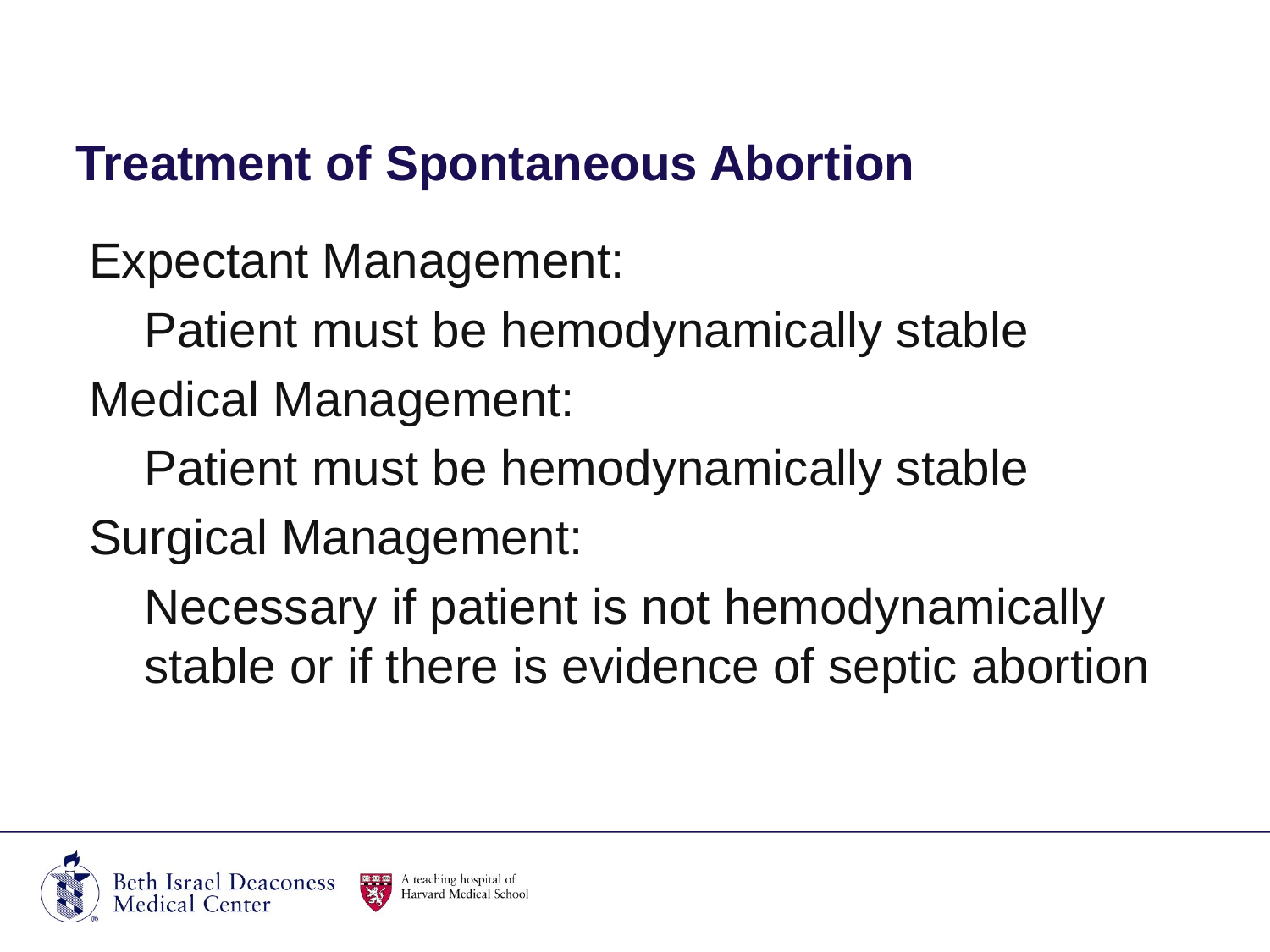

# Treatment of Spontaneous Abortion
Expectant Management:
Patient must be hemodynamically stable
Medical Management:
Patient must be hemodynamically stable
Surgical Management:
Necessary if patient is not hemodynamically stable or if there is evidence of septic abortion

## Slide 28
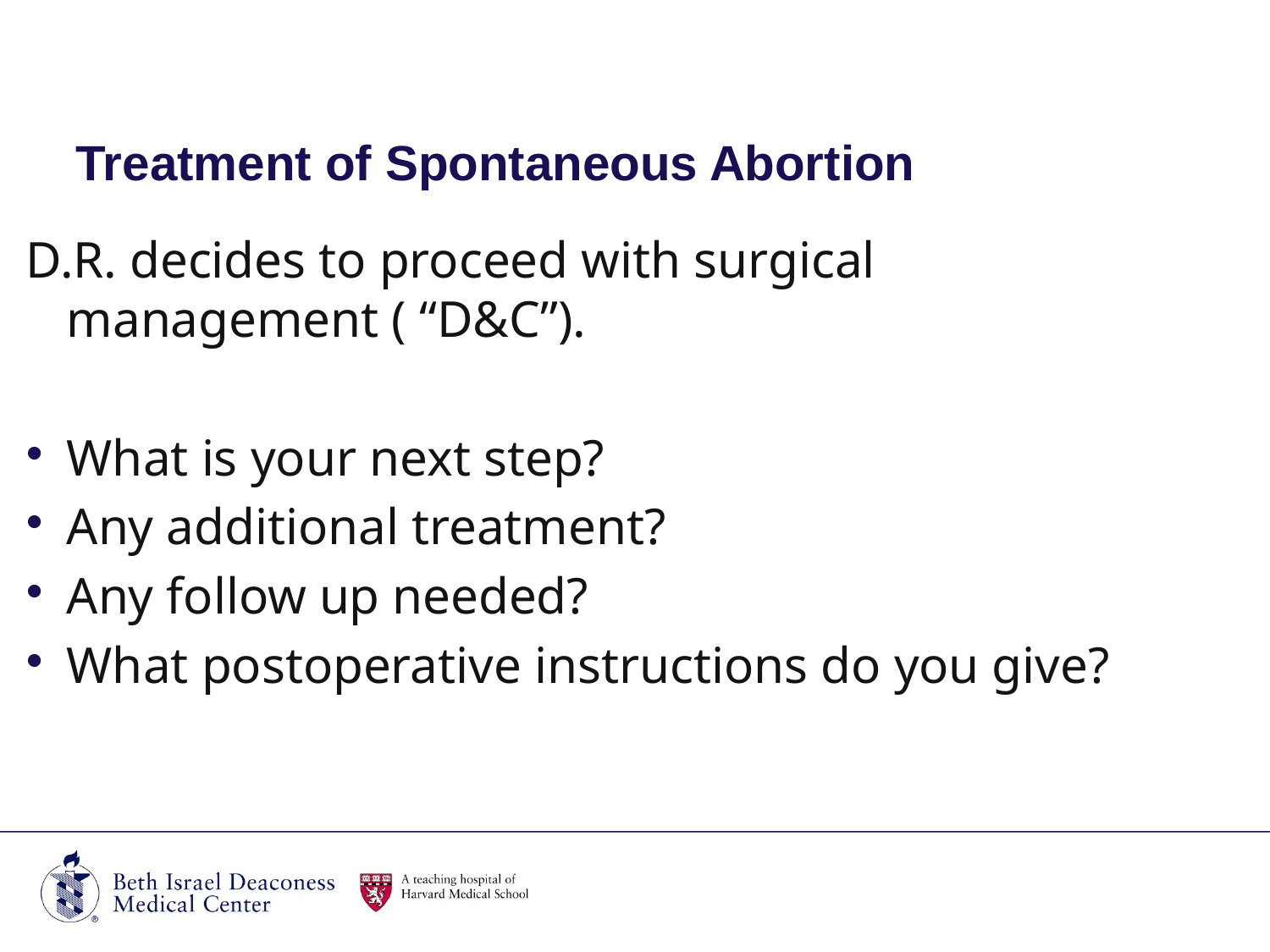

# Treatment of Spontaneous Abortion
D.R. decides to proceed with surgical management ( “D&C”).
What is your next step?
Any additional treatment?
Any follow up needed?
What postoperative instructions do you give?

## Slide 29
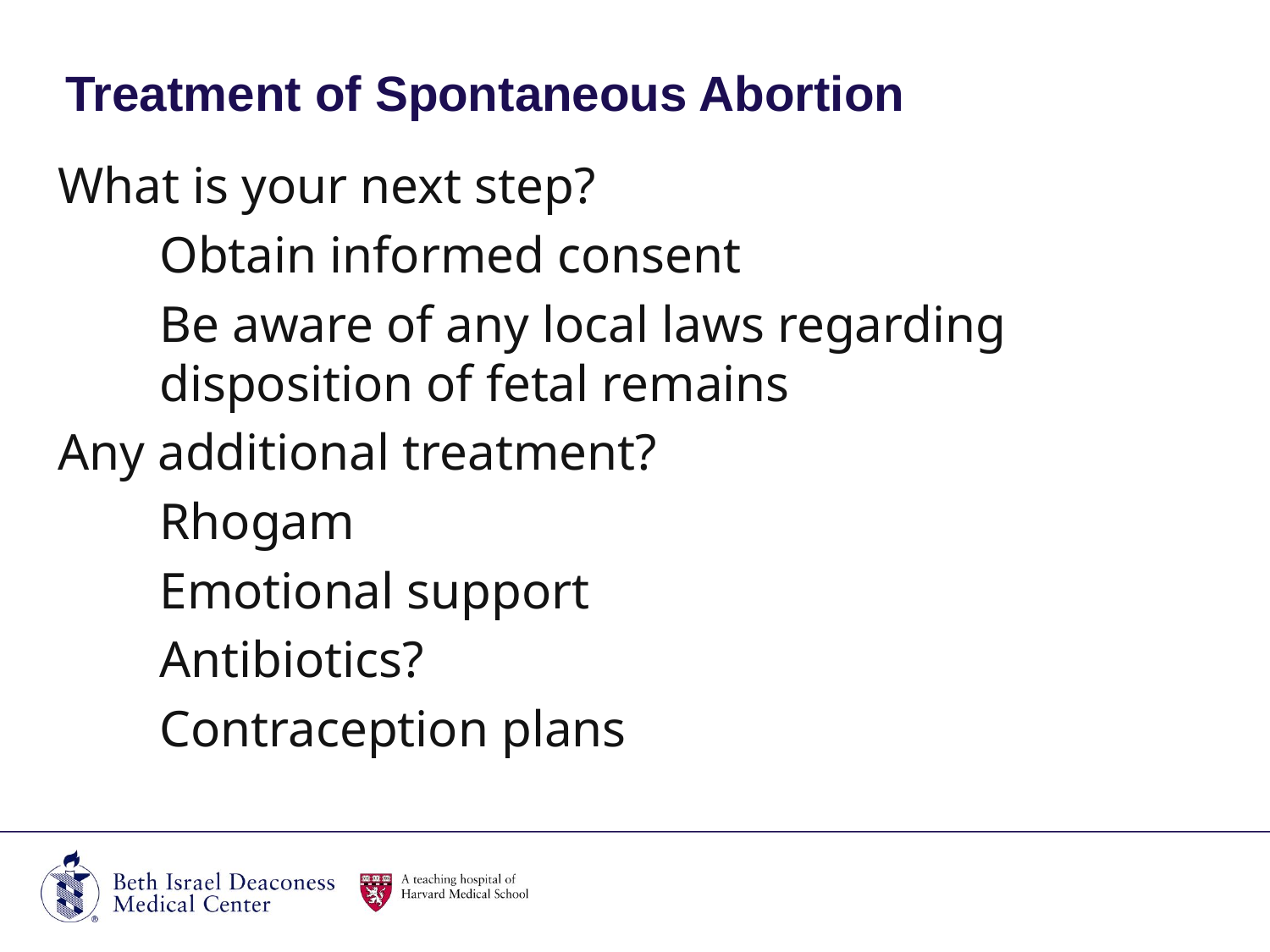

# Treatment of Spontaneous Abortion
What is your next step?
Obtain informed consent
Be aware of any local laws regarding disposition of fetal remains
Any additional treatment?
Rhogam
Emotional support
Antibiotics?
Contraception plans

## Slide 30
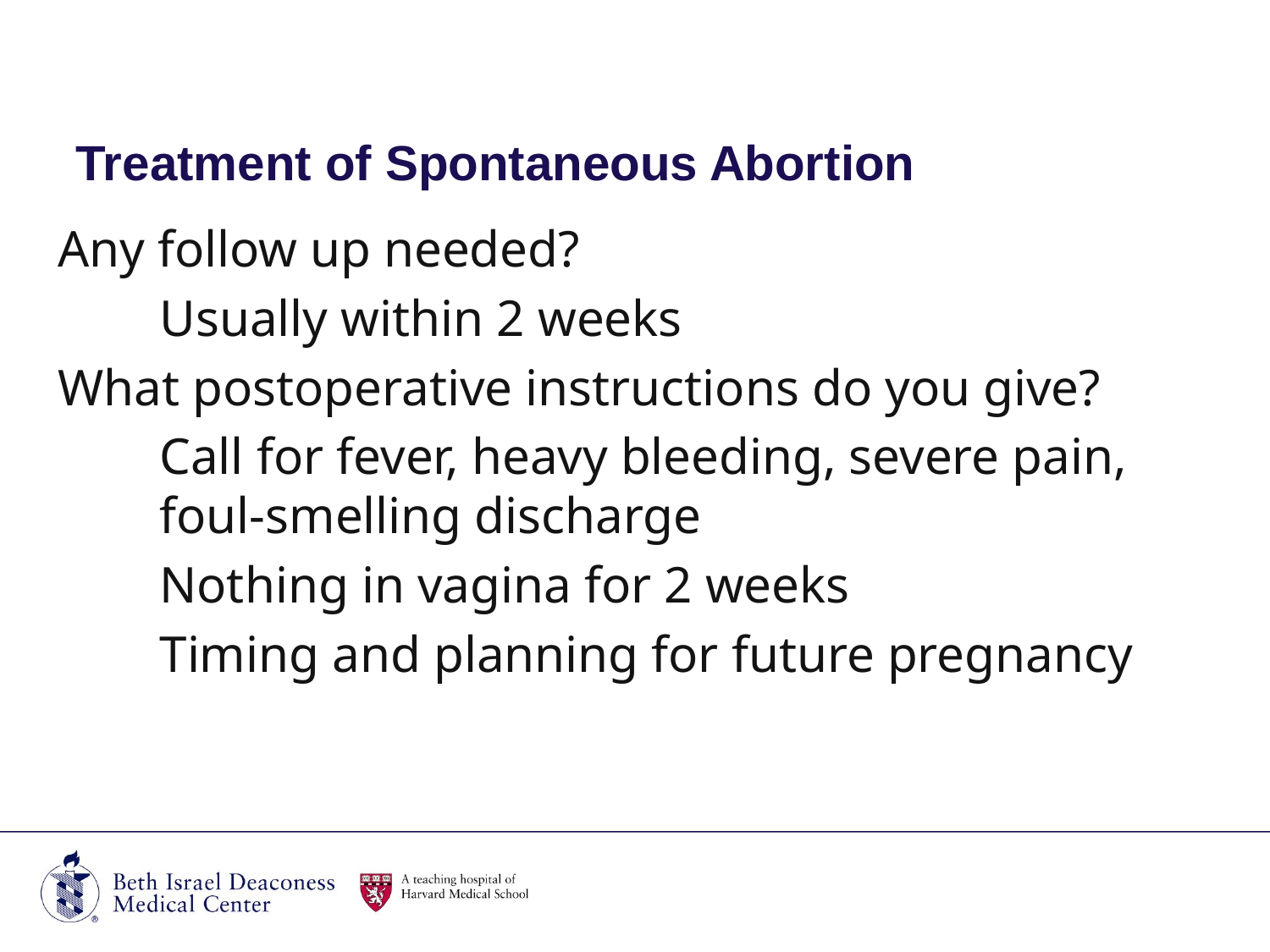

# Treatment of Spontaneous Abortion
Any follow up needed?
Usually within 2 weeks
What postoperative instructions do you give?
Call for fever, heavy bleeding, severe pain, foul-smelling discharge
Nothing in vagina for 2 weeks
Timing and planning for future pregnancy
