# Supplementary material for: Teaching Patient Handoffs to Medical Students in Obstetrics and Gynecology: Simulation Curriculum and Assessment Tool
Source: MedEdPORTAL. 2016 Oct 2;12:10479. doi: 10.15766/mep_2374-8265.10479 (PMC6440488; doi:10.15766/mep_2374-8265.10479)
Supplement: Supplementary file 1 — A. Patient Handoffs in Obstetrics and Gynecology.pptx B. Approach to Diagnosis and Management of First Trimester Bleeding.pptx C. Patient Handoffs in Obstetrics and Gynecology Narrated.mp4 D. Approach to Diagnosis and Management of First Trimester Bleeding Narrated.mp4 E. Handoff Skills Speakers Notes.docx F. First Trimester Bleeding Speakers Notes.docx G. Simulation Guide.docx H. Role Play Description.docx I. Trainee Simulation Information Cards.doc J. Ultrasound Report.docx K. Student Assessment Tool.docx L. Debrief Checklists.docx [file mep-12-10479-s001.zip › E. Handoff Skills Speakers Notes.docx]

**Appendix E: Speakers Notes for Patient Handoffs in Obstetrics and Gynecology: A Primer**

Slide 1

Patient Hand Offs in Obstetrics and Gynecology: a Primer

Slide 2

In this module, we will review the importance of patient hand offs, talk about what a hand off is and is not, review types of hand offs, and provide an model for structuring an efficient hand off.

Slide 3

There are no financial disclosures for the authors of this module.

Slide 4-5

To begin, we can review why patient hand offs are important. Good Hand offes helpo to prevent medical error. Many studies have shown that medical error is common, serious and preventable. Studies going back to the 1980s, such as this report on over 30 thousand patients in 1984, demonstrate 3-16% of admissions are complicated by adverse events, with 50-70% due to inadequate hand off.

Slide 5

Slide 6

Let’s think about which patients might be at greatest risk for medical error. What characteristics of patients or providers might increase the chances of a medical error?

Slide 7

Higher risk patients include

Post-operative patients

Patients with complex or multiple medical conditions

Patients in the emergency department

Older patients

Patient with poor communication abilities:

Including non-native speakers, hearing impaired, patients with developmental delay

Another group of patients at risk are patients under the care of inexperienced providers.

Slide 8

To put the risk of medical error in perspective, how risky is being an in-patient?

Activities such as working in a nuclear power plant, riding a European railway, or flying on a commercial airline have a risk of death of approximately 1 in 100 thousand.

Driving a car and working in chemical manufacturing have a risk of death per encounter of between 1 in 1000 and 1 in 100 thousand,

Whereas activities such as bungee jumping and extreme mountain climbing have a risk of approximately 1 in 1000. Unfortunately, the risk of death for inpatient medical care ranks among the last group.

Slide 9

In 2001 the Institute of Medicine published *To Err is Human: Building a Safer Health System*. The report highlighted areas where the organizational systems of medical care might be improved, and noted that “handoffs are where safety often fails first.”

Slide 10

In 2006, the World Health Organization Collaborating Centre on Patient Safety (Solutions), the World Alliance for Patient Safety, and the Commonwealth Fund joined to launch the “High 5s” initiative, which among other initiatives prioritized prevention of patient care errors through better hand offs.

Slide 11

There are many areas of medicine which can be improved. The use of time outs and checklists have decreased the risks of surgical errors such as wrong site, wrong side, wrong patient, and retained objects. Electronic medical records can help prevent medication errors and improve stewardship of antibiotic use. Infrastructure improvements can help prevent falls and reduce injury in medical facilities. Individual effort at hand hygiene is one of the most effective infection prevention tools available. Finally, health care is a 24 hour a day, team activity. In order to provide excellent, ongoing continuity of care in a team-based setting, performing effective hand offs is essential to patient safety.

Slide 12

Why do we need to learn to perform a hand off?

Patient hand offs are essential to patient safety

Communication failure is the leading cause of preventable medical errors

A concise summary of detailed information is needed to provide ongoing care, particularly for complicated patients

A patient may have 2,3 or more care teams in a day.

Often the least experienced member of a care team is responsible for giving or receiving a hand off, particularly overnight and on weekends, when there might be less supervision or support.

We can learn to use algorithms to communicate more effectively.

Slide 13

There are regulatory reasons for learning to perform effective hand offs; for example The Joint Commission lists as its National Patient Safety Goal 2:

Implement a standardized approach to hand-off communications, including the opportunity to ask and respond to questions

Slide 13

Additionally the ACGME states that for residencies, “Sponsoring institutions and programs must ensure and *monitor* effective, structured handover processes to facilitate both continuity of care and patient safety. Programs must ensure that residents are competent in communicating with team members in the handover process”.

Slide 14

Let’s now look at what makes up an effective hand off.

The American College of Obstetrics and Gynecology states that an hand off is : The transfer of patient information and knowledge, along with authority and responsibility. It should include an opportunity to answer questions, clarify, and confirm the information being transmitted.”

This implies that a hand off includes both clinical information about a patient, but also important personal details to provide context.

The hand off means that the on-coming provider accepts responsibility and also is given authority to act, and make clinical decisions. This implies that the hand off gives the receiver all essential information, but also a guide as to what the likely concerns may be.

Finally the hand off needs to use closed loop communication- the receiver needs to be able to ask questions for clarification, and the giver needs to be able to confirm.

Slide 15

The hand off can occur virtually, over the phone, on in person. There are benefits to each method.

Slide 16

Computerized hand offs allow for more comprehensive patient information, give a more standardized organization of information, and can help prevent missing or overlooking key facts. However, a computerized list must be updated regularly, and more importantly, does not allow for non-verbal communication, and tends to be uni-directional, with little opportunity to ask questions or receive clarification.

Slide 18

Face-to-face sign out allows for back and forth communication, gives the chance for non-verbal communication, but can be more easily interrupted by distractions.

Slide 19

When participating in a face-to-face hand off it is important to

- Minimize background noise- go to a private area
- Stop ongoing clinical duties if at all possible
- Be in an area with Appropriate lighting- especially for a late night signout
- Use electronic or paper media with Ample space to take notes
- It’s important to have a consistent time, place and structure to standardize the hand off as much as possible and limit errors.

Slide 20

There is also good evidence that practice makes perfect: a study of students who had the opportunity to practice standardized hand off skills showed improvement in reaching “expert” level hand offs in a OSCE, from 27% rated as expert to 67% rated as expert.

Slide 21

Hand offs work best if there is a standardized format. We use the SBAR method. SBAR stands for Situation, Background, Assessment and Recommendations. This method was chosen for its simplicity, ease of use, and generalizability to virtually any service or type of patient encounter. Additionally, SBAR is used by other professionals, particularly in nursing. Using SBAR can improve inter-disciplinary communication. By creating a shared mental model, communication is enhanced.

This slide illustrates an SBAR sign out sheet for a hypothetical post-partum patient.

Slide 22

Here are a few examples:

Situation: this gives a one-line introduction to the patient and the reason for hospitalization:

- *48 yo G2P2 POD 1 s/p uncomplicated TAH for fibroids*
- *75 yo G5P3 POD 5 s/p extensive debulking procedure including bowel resection for ovarian cancer*

Slide 23

Background: this section gives pertinent details about patient status and history, for example

*Pertinent details about the post-operative care (diet, hematocrit drop, epidural in place, etc.)*

*Relevant Past Medical history (for example: history of DVT, HTN, asthma)*

*Include Family or Social History only if relevant (anxious family, smoker)*

Slide 24

Assessment is the area to report any abnormal findings, current concerns and expected, likely or possible events that the oncoming provider can anticipate.

It is also helpful when giving hand off for a service, to let your colleagues know which patients are most ill, which patients will need more work, and which patients are more stable. Giving a list of patients with their acuity will help your colleagues structure their workflow.

Slide 25

Under recommendations, you do not need to re-summarize, but simply recommend aplan of action or care.

This includes any to-do items and if-then statements for predictable complications, such as , a CBC is pending for a patient with a large intr-operative blood loss. check the CBC, and if Hematocrit is <22% transfuse 2 units Packed Red Blood Cells.

These recommendations are not intended to replace clinical decision making but to pass on to a colleague any decisions that have already been made pending results, alert them to potential or expected complications and give guidance based on known patient information.

Slide 26

Let’s work through a few examples. Remember, we want to

*Give a single sentence introduction for Situation*

*Concentrate on synthesis and consolidation of the history for Background*

*Provide a list of concerns including a comparison of severity of illness with other patients on the service for Assessment*

*Provide a to-do list and a set of if/then instructions for the Recommendations*

Slide 27

Here is a patient to practice your SBAR technique.

*JB is a 36 yo G3P0SAb3 who underwent open myomectomy today for a large symptomatic fibroid uterus. The surgery was complicated by a large estimated blood loss of 2000 mL, and an estimated weight of the fibroids of 1300gm. The surgery took 5 hours (2 hours longer than anticipated) and the patient is still in the PACU.*

Slide 28

*The patient has a history of asthma, obesity (BMI 39), borderline hypertension and sleep apnea. She takes iron and prenatal vitamins. She has had 2 spontaneous abortions and strongly desires future fertility. She has no surgical history. She has no history of abnormal paps, a distant history of chlamydia infection, and is sexually active with her husband of 2 years, who is the father of her 2 pregnancies. She does not use tobacco, alcohol or other drugs and works as a principal at a middle school. She has an allergy to amoxicillin. She has a health care proxy on file naming her mother as proxy.*

Slide 29

*The patient had a preoperative Hematocrit of 41% and intraoperative hematocrit of 30%. She remained normotensive during the surgery.Urine output was 300 mL and she received 2.4 liters of crystalloid during surgery. She has a foley catheter in place with no other drains. She did not receive any NSAID drugs peri-operatively.*

*The surgeon spoke briefly with the patient and with her husband and mother after surgery.*

Slide 30

*Tonight she will need a post operative check, there is a CBC pending, and her family is asking to speak with the doctors. There are 3 other patients on the service, 2 are going home tomorrow and one is still in the operating room with a vascular complication that required the team to call in a vascular surgeon to repair the injury.*

Slide 31

How will you describe the **Situation** for this patient?

Slide 32

Our team gave the **Situation** as: 36 yo G3P0 s/p open myomectomy for large fibroids, complicated by EBL 1200 and a prolonged surgery.

Slide 33

How will you describe the **Background** for this patient?

Slide 34

Our team gave the **Background** as: she has Asthma, borderline hypertension not on meds, obesity, and sleep apnea

- - Her family are here and anxious
  - Her pre-op hematocrit 41%, intra-op 30%
  - And her urine output 300mL was intra-operatively

Slide 35

How will you describe the **Assessment** for this patient?

Slide 36

Our team gave the **Assessment** as: she has had Significant blood loss

- - There is a Concern for sleep apnea
  - there is a Concern for her family
  - and she is the Second- most concerning patient overnight

Slide 37

How will you describe the **Recommendations** for this patient?

Slide 38

Our team gave the **Recommendations** as:

- - you need to Check the CBC: she may need transfusion
  - you should Monitor O2 saturation, pain control, urine output. May need more fluids, or medication adjustment
  - she May need respiratory therapy evaluation
  - Reassure family.

Using SBAR can help you to communicate efficiently and effectively, help avoid medical errors, and decrease the stress of caring for patients for yourself and your colleagues. A good hand off can lead to a good night’s sleep for you, smoother work flow for your colleagues, and safer care for your patients.
