# Supplementary material for: Teaching Patient Handoffs to Medical Students in Obstetrics and Gynecology: Simulation Curriculum and Assessment Tool
Source: MedEdPORTAL. 2016 Oct 2;12:10479. doi: 10.15766/mep_2374-8265.10479 (PMC6440488; doi:10.15766/mep_2374-8265.10479)
Supplement: Supplementary file 1 — A. Patient Handoffs in Obstetrics and Gynecology.pptx B. Approach to Diagnosis and Management of First Trimester Bleeding.pptx C. Patient Handoffs in Obstetrics and Gynecology Narrated.mp4 D. Approach to Diagnosis and Management of First Trimester Bleeding Narrated.mp4 E. Handoff Skills Speakers Notes.docx F. First Trimester Bleeding Speakers Notes.docx G. Simulation Guide.docx H. Role Play Description.docx I. Trainee Simulation Information Cards.doc J. Ultrasound Report.docx K. Student Assessment Tool.docx L. Debrief Checklists.docx [file mep-12-10479-s001.zip › F. First Trimester Bleeding Speakers Notes.docx]

**Appendix F: Speakers Notes for An Approach to Diagnosis and Management of First Trimester Bleeding**

Slide 1

An Approach to Diagnosis and Management of First Trimester Bleeding

Slide 2

This presentation will discuss the definitions, etiology and management of first trimester bleeding. First trimester bleeding is one of the most common complications of pregnancy, affecting as many as ¼ of all pregnancies. Although half of pregnancies with first trimester bleeding will go on to have normal outcomes, 12-15% of all recognized pregnancies will end in spontaneous abortion, or miscarriage. All physicians should understand the treatment options available to women and their families for a diagnosis of spontaneous abortion.

Slide 3

Let’s review some medical definitions used in referring to pregnancy complications

Abortion is a medical term for a pregnancy loss, and is usually limited to pregnancy loss prior to viability.

Pregnancy loss after viability is referred to as a fetal demise, intrauterine fetal demise, or commonly as a stillbirth.

Any bleeding during the first or early second trimester can be referred to as a threatened abortion.

Induced abortion is the termination of pregnancy, either voluntary, also called elective, or therapeutic, for the health of the woman.

Spontaneous abortion refers to a pregnancy loss that happens from a complication of pregnancy prior to viability of the fetus. The etiology of spontaneous abortion often is not known; cytogenetic studies of the fetal tissue can be performed to evaluate for chromosomal abnormalities, which are thought to be the most common cause for first trimester pregnancy loss.

Complete abortion refers to a spontaneous abortion in which all fetal and placental tissue passes from the uterus, and the cervix closes. Complete abortion can be diagnosed by history and confirmed by ultrasound.

Incomplete abortion refers to a spontaneous abortion in which not all fetal or placental tissue passes, and the cervix remains open. Incomplete abortion is diagnosed by physical exam of the cervix, with ultrasound imaging showing tissue remaining in the endometrial cavity. Commonly the cervix will be evaluated with a sterile ring forceps or sterile digital exam to determine if it is open.

An inevitable abortion usually refers to a pre-viable pregnancy in which the cervix has dilated but fetal demise has not yet occurred, and is diagnosed by physical exam and ultrasound imaging.

A missed abortion refers to a pre-viable pregnancy in which the fetus has died, but the cervix has not yet dilated. This diagnosis is the more common presentation of early pregnancy loss in higher resource settings where early ultrasound is commonly performed.

A septic abortion refers to a pre-viable pregnancy loss complicated by uterine infection. Septic abortion can be lethal for the woman. Septic abortions can occur as a result of complications of unsafe surgical abortions, for example in communities where legal, safe, voluntary termination is not available, and can be significant causes of maternal morbidity and mortality. Septic abortion can also occur from PID, or other maternal infections.

Slide 4

Now that we have some definitions, what diagnoses should be considered when a woman presents with first trimester bleeding? Not all bleeding is from a pregnancy loss, and using an organized approach will help prevent errors of premature closure in our thinking.

One approach to developing a differential diagnosis for any condition is to organize etiologies by anatomical structures or organ systems; another is by type of pathology. We can use both approaches to generate our list.

Bleeding during pregnancy might be due to:

A Vulvar or vaginal source such as a wound or trauma, including accidental injury or assault, infection, or neoplasia such as condyloma acuminata

A cervical source: many infections, such as chlamydia, bacterial vaginosis, Trichomoniasis, or other STI’s; a neoplastic process such as cervical dysplasia or a polyp; trauma from normal sexual activity can also cause cervical bleeding during pregnancy.

A uterine source: a pregnancy may be complicated by bleeding from between the uterine wall and the placenta, referred to as a sub-chorionic hematoma, or perigestational bleeding; any of the types of abortions, such as missed, inevitable, incomplete, or septic. Conditions such as fibroids and adenomyosis do not generally cause bleeding during pregnancy.

The fallopian tubes are the most common location of ectopic pregnancies, which usually present as first trimester bleeding with pelvic pain. Ectopic pregnancies make up approximately 1.5% of pregnancies and need to be considered in any woman who is pregnant with bleeding. In fact, a first trimester pregnancy with bleeding should be assumed to be an ectopic pregnancy until proven otherwise.

The ovaries can be the location of an ectopic pregnancy. The ovaries are unlikely to contribute to first trimester bleeding in other ways, though.

Endometriosis is not thought to play a role in pregnancy loss, although it can be a cause for infertility.

Looking outside the reproductive system, first trimester bleeding from other sources may be mistaken for vaginal bleeding. Hematuria from a urinary tract infection, or a gastrointestinal source such as hemorrhoids may be mistaken for vaginal bleeding and should be considered in the differential diagnosis.

The etiology of pregnancy loss is often specific to the pregnancy and the fetus, for example a genetic or chromosomal abnormality such as a trisomy, a monosomy like Turner syndrome, or a molar pregnancy. Less common causes for pregnancy loss might include maternal medical conditions such as uncontrolled diabetes, smoking, or auto-immune diseases. Although it is often important to a woman and her family to discover the cause of a pregnancy loss, this usually cannot be determined at the time of diagnosis. Testing for the cause of pregnancy loss can be carried out and should be offered to the patient. Additionally, many women will blame themselves for a pregnancy loss. It is appropriate, and kind, to reassure a woman that she did not cause the pregnancy loss.

Slide 5

We can now work through a case history.

D.R. is a 34 yo G3P2 woman who presents to your office complaining of spotting since last night. Her LMP was 8 weeks ago and she had a positive pregnancy test 10 days ago. She is concerned, as she does not remember any bleeding during her first two pregnancies.

Slide 6

What more do you want to know about DR’s history?

Slide 7

We should take a basic gynecologic history, including the last menstrual period, any irregularities to the menstrual cycle; prior pregnancy history; sexual activity including use of contraceptives; a general medical, surgical, social and family history; any medications or allergies, and a review of systems.

Slide 8

We find out that DR had

An abnormal pap with CIN 2-3 on LEEP 4 years ago
she denies any STI, not currently using contraception. She was not planning to become pregnant.

Her Menstrual history includes menarche at age 12, cycles every28-35days and lasting 5-7 days with heavy flow, using 8-10 pads per day.

Her Obstetric History includes 2 uncomplicated vaginal deliveries at term, both over 4000gm

Her medical History is significant for depression with suicide attempt at 19 and a breast fibroadenoma

Her surgical History includes Dilation & Curettage for elective Abortion at age 18 and a Loop electrode excisional cervical biopsy at age 30.

Her only medication is paroxetine

Her family History is notable for a Sister with endometriosis, Mother and MGM with breast cancer age 40 and 66. Maternal grandfather with multiple sclerosis. Paternal grandfather with prostate cancer age 85.

DR works as a labor and delivery nurse, lives with husband and 2 children. Drinks 4-7 alcoholic beverages per week and smokes ½ pack of tobacco cigarettes per day. No other substance use. There is a history of domestic violence with an assault by her husband last year.

Slide 9

At this point, our differential diagnosis is quite wide. Are there findings on physical exam that you might look for to help narrow the possibilities?

Slide 10

As with any patient presenting for evaluation, the physical examination should include vital signs, evaluation of the head and neck, a cardiovascular exam, auscultation of the lungs, and abdominal exam and evaluation of the extremities. Lastly a thorough pelvic exam is indicated.

When performing the physical exam, think of findings you need to know to help eliminate or confirm the possible diagnoses.

Slide 11

On physical exam, our patient has a temperature of 37 degrees, blood pressure 110/72, heart rate of 80 and respiratory rate of 14

The head and neck exam is notable for moist, pink mucosa, a clear oropharynx, normal dentition, and a normal sized thyroid gland.

The cardiovascular exam shows a regular rate and rhythm on auscultation of the heart sounds.

The lung fields are clear.

The abdominal exam is soft, with mild tenderness to deep palpation over the supra-pubic region, with no costo-vertebral angle tenderness, no masses, rebound or guarding.

The extremities show no edema or varicosities.

Do these findings make any diagnosis more or less likely?

Slide 12

On Pelvic Exam,

The external genitalia are those of a normal female, with no lesions or trauma.

Vagina has a small amount of dark blood and clot, estimated as 10 mL.

The Cervix parous with no cervical motion tenderness, and the cervical os open to fingertip.

The Uterus is mid-position, 8 weeks’ size, non-tender

The adnexae are without masses or tenderness

Rectal exam notable for external hemorrhoids

With these findings we can start to limit our differential diagnoses. The cervix is open, suggesting an incomplete or inevitable spontaneous abortion. There is no evidence of trauma, infection of neoplasm. It also seems less likely that the source of bleeding is non-gynecologic, such as a GI or urinary source.

Also, our patient is hemodynamically stable with an unremarkable general physical exam. There is no suggestion from the exam of other medical illnesses, although the history still contains some elements that might affect our management.

Slide 13

What laboratory tests are indicated?

Many tests are commonly ordered to evaluate a patient with a threatened abortion Although it may be tempting to “order everything”, especially if another provider has already drawn blood samples as might occur in an emergency department when starting an IV, we can use the information we have gathered on history and physical exam to limit unnecessary tests while assuring we have all the information we need to care for the patient appropriately.

Slide 14

Some commonly used lab tests include:

Complete blood count, to evaluate for anemia, infection, and thrombocytopenia

Blood type and rh status: it is important to know the rh status for any pregnant woman. Rh-negative women should receive immunoglobulin to prevent allo-immunization, even with a minor first trimester bleeding episode.

Blood type should be determined in case of the need for transfusion.

The utility of electrolytes, renal or liver chemistry panels is limited in the initial evaluation of first trimester bleeding, usually these tests are not necessary in a woman with no underlying medical illnesses. IF a patient is found to have an ectopic pregnancy and medical management is desired, a chemistry panel should be obtained to insure the patient has no contraindication to methotrexate.

Coagulation studies are not generally needed for evaluation or management of a hemodynamically stable patient.

What about a pregnancy test? Pregnancy should be confirmed either by a urine or blood HCG test. A qualitative HCG test can be used to determine pregnancy. If the pregnancy has not been determined to be intra-uterine by ultrasound, a quantitative HCG can be useful. If the HCG level is greater than 1500-2000, an intrauterine pregnancy should be identifiable on ultrasound. If the level is greater than 2000 and no IUP is seen, the concern for an ectopic is much higher.

Other tests that might be considered include a wet prep to look for candidal infection or bacterial vaginosis, nucleic acid amplification tests for gonorrhea, chlamydia, and trichomonas; serology tests for syphilis and HIV. Screening for cervical cancer can be considered if not up to date.

Slide 15

Laboratory test results for our patient include

A white cell count of 7.4, Hematocrit of 31%, and platelets of 197 thousand.

Blood type is O negative.

A urine pregnancy test is positive and a beta HCG level is 47,443.

Slide 16

Tests for sexually transmitted infections and other vaginal infections are all negative.

Slide 17

We have now narrowed our differential diagnosis to inevitable vs incomplete abortion, vs ectopic pregnancy.

Our next step is to obtain imaging studies. What imaging method would you order?

Slide 18

Ultrasound is the gold standard for pregnancy imaging and can locate the pregnancy as well as determine if there is a living fetus present. Additionally, ultrasound is commonly done by the provider at the bedside with little discomfort for the patients. CT scans are not as useful for the pelvic soft tissues and expose both the patient and fetus to radiation. MRI is usually not necessary to determine the location of a pregnancy. Both CT and MRI are considerably more expensive than ultrasound.

Slide 19

For our patient,

Imaging Tests

Ultrasound shows an intrauterine singleton pregnancy, consistent with 8 weeks 1 day, with no fetal cardiac activity. There is a small subchorionic bleed. The adnexae appear normal and there is no free fluid in the pelvis.

What is our diagnosis?

If you said incomplete, spontaneous abortion you are correct. The cervix is dilated and admits a fingertip; the ultrasound shows no cardiac activity.

Slide 20

We can now think about treatment options.

How will we counsel patient DR?

IN general, there are three treatment options available for spontaneous abortion: expectant management with continued surveillance; medical management with misoprostol; and surgical management with D&C, or dilation and curettage (the term D&E, or dilation and evacuation, is used for pregnancy beyond the first trimester).

Slide 21

Expectant management means allowing the body to continue to try to expel the pregnancy naturally. With expectant Management, the advantages include that it is Non-invasive and can be recommended for patients who do not want any intervention.

However, expectant management is:

Unpredictable

And May take days to weeks to complete

The Bleeding and cramping may be prolonged

Risk of subsequent need for surgery up to 20%

Slide 22

We can offer medical management with misoprostol, a synthetic prostaglandin, PGE 1, which causes myometrial contractions.

The advantages of Medical Management include that it is:

Relatively non-invasive

Usually effective within 24-48 hours

- - - 70-90% within 48 hours

Risk for subsequent need for surgery

- - - 10-30%

Slide 23

Surgical management is preferred by some women as it is relatively quick, with less discomfort, provides greater control over timing and is quite effective, with only a 5-10% failure rate.

Slide 24

If a woman chooses misoprostol, there are a variety of medication regimens detailed in the literature.

The World Health Organization from 2007 recommends

Missed abortion: 800 mcg per vagina OR 600 mcg sublingually (each of these is a single dose)

Incomplete abortion: 600 mcg orally (single dose)

Common Regimen: 400 mcg vaginally every four hours for four doses.

Increased effectiveness with vaginal administration

Decreased side effects

Lower risk of medical complications than with surgery.

Slide 25

Where as surgical management does have real risks of uterine perforation or infection.

Slide 26

IF a patient is hemodynamically unstable, or if a patient has a septic abortion, surgery is the only acceptable management

Slide 27

After we inform our patient DR of the sad news, and explain her options, she decides to go forward with surgical management.

- What is your next step?
- Any additional treatment?
- Any follow up needed?
- What postoperative instructions do you give?

Slide 28

When a patient decides on surgical management, we need to obtain informed consent. This includes giving the patient the information required to make a reasonable decision to undergo a procedure, including listing common or predictable adverse effects or outcomes, expected benefits, and alternative treatments or course of action. One needs to be aware of any local laws regarding disposition of fetal remains; some states require offering the option of cremation or burial for fetal remains. If a patient desires cytogenetic testing to help determine the cause of the pregnancy loss, the lab should be contacted to process the tissue.

Additional treatment includes rh immunoglobulin for RH-negative patients. It is appropriate to offer immediate and ongoing emotional support for what is often a difficult transition; some women may benefit from social work or counseling services.

There is no need for antibiotic treatment after a surgical D&C unless the patient has an infection.
All women should be offered contraception after a pregnancy loss. Although a woman might want to conceive again, she may also want to wait, or may have changed her mind regarding pregnancy.

Slide 29

Postoperative instructions should be given to the patient. These should include directions to

Call for fever, heavy bleeding, severe pain, or foul-smelling discharge. Pelvic rest is usually indicated, meaning placing nothing in the vagina for 2 weeks.

A follow up visit with a gynecologist or family practitioner is recommended for within 2 weeks. If cytogenetic testing was performed the results can be reviewed at this time. Timing and planning for future pregnancy can also be discussed.

This concludes the preparatory session, An Approach to Diagnosis and Management of First Trimester Bleeding. You will have a chance to discuss this and other cases with your colleagues and instructors. Good luck with your simulation session.
