# Supplementary material for: Teaching Patient Handoffs to Medical Students in Obstetrics and Gynecology: Simulation Curriculum and Assessment Tool
Source: MedEdPORTAL. 2016 Oct 2;12:10479. doi: 10.15766/mep_2374-8265.10479 (PMC6440488; doi:10.15766/mep_2374-8265.10479)
Supplement: Supplementary file 1 — A. Patient Handoffs in Obstetrics and Gynecology.pptx B. Approach to Diagnosis and Management of First Trimester Bleeding.pptx C. Patient Handoffs in Obstetrics and Gynecology Narrated.mp4 D. Approach to Diagnosis and Management of First Trimester Bleeding Narrated.mp4 E. Handoff Skills Speakers Notes.docx F. First Trimester Bleeding Speakers Notes.docx G. Simulation Guide.docx H. Role Play Description.docx I. Trainee Simulation Information Cards.doc J. Ultrasound Report.docx K. Student Assessment Tool.docx L. Debrief Checklists.docx [file mep-12-10479-s001.zip › G. Simulation Guide.docx]

| **Appendix G: MedEdPORTAL Simulation Case Template**  SIMULATION CASE TITLE: Teaching Patient Handoffs to Medical Students in Obstetrics and Gynecology: A Simulation Curriculum and Assessment Tool  **AUTHORS: Celeste Royce, MD, Monica Mendiola MD, Hope Ricciotti MD, Katharyn Atkins MD** | |
| --- | --- |
| **PATIENT NAME: Susan Quinn**  **PATIENT AGE: 31**  **CHIEF COMPLAINT: “bleeding since getting up this morning”** | |
|  | |
| **Brief narrative description of case**  *Include the presenting patient chief complaint and overall learner goals for this case* | The first learner (“First Intern”) has the role of the OBGYN intern on call. The First Intern is called into the simulation after being told the patient's name, chief complaint of vaginal bleeding for several hours, age and gravidity/parity.  First Intern interviews the patient and communicates with the New Hire Nurse, who stays in the scene unless getting transfusion equipment. First Intern obtains history from patient, then performs physical exam.  When First Intern asks to do pelvic exam, New Hire Nurse hands learner appropriate index card with results of initial physical exam at that time in the scenario (findings change through scenario). Labs and imaging can be ordered by First Intern. The patient’s clinical status may change during the simulation.  The First Intern will be paged out of the simulation and a second learner (“Second Intern”) will enter.  Second Intern is sent in, and First Intern provides a handoff.  Second Intern is expected to have opportunity to ask questions of First Intern.  This repeats for up to four learners as Interns.  Intern Actions Include:  Take History  Interpret vital signs  Order fluids, imaging and labs  Perform physical exam  Interpret physical findings and labs  Order ultrasound  Order blood products and manage fluids  Interpret ultrasound  *Inform patient of diagnosis and recommendations for surgery (*not for clerkship student level learner)  Handoff patient to another provider.  The simulation can be adapted for up to 4 learners.  An alternative scenario allows each learner to manage the entire simulation as the Intern and give handoff directly to Attending physician. |
| **Primary Learning Objectives**  *What should the learners gain in terms of knowledge and skill from this case? Use action verbs and utilize Bloom’s Taxonomy as a conceptual guide* | A. Primary: Perform an appropriate patient handoff  Manage first trimester vaginal bleeding    B. Secondary: Initiate management of acute blood loss  Recognize emergent clinical findings  Deliver bad news-*optional*  Obtain informed consent- *optional* |
| **Critical Actions**  *List which steps the participants should take to successfully manage the simulated patient. These should be listed as concrete actions that are distinct from the overall learning objectives of the case.* | Take history  Interpret vital signs and lab results  Order fluid resuscitation and O2  Order blood products  Interpret physical findings and ultrasound results  Decision to perform surgery  Obtain informed consent (not included in clerkship level learners)  Perform handoff of patient to next provider |
| **Learner Preparation**  *What information should the learners be given prior to initiation of the case?* | Prior to the simulation, learners participate in a review of management of first trimester vaginal bleeding and of handoffs (Appendices A and B). Learners are reminded of the goals of the simulation: management of the patient with acute blood-loss anemia in the setting of spontaneous abortion, and performance of a successful handoff.  Each learner will have an opportunity to give and receive a handoff. |

| Initial Presentation | | | |
| --- | --- | --- | --- |
| **Initial vital signs** | Initial Vital signs:  Blood Pressure 120/70; Heart Rate100; Respirations 18; Temp 37.0; O2 saturation 99% | | |
| **Overall Appearance**  *What do learners see when they first enter the room?* | Each learner walks into simulation room with the New Hire Nurse, who explains s/he is new, and would like to hear everything the learner says, “to learn more”. The New Hire Nurse introduces each learner to patient, Susan Quinn, a 31 year old, G2P0 SAB1 female who presented this morning with vaginal bleeding, and who is 10 weeks pregnant. The New Hire Nurse informs the First Intern that the New Hire Nurse started an IV and drew “some blood”.  Each learner introduces him/herself to the patient and is expected to take or confirm the patient’s history. There are accurate, real-time vital signs present on the bedside monitor. There is an IV set up, O2 available, and a code cart. The learner can write down history and findings on paper, electronic device or wallboard. (In our experience the first learner to enter the scenario usually has adequate time to obtain a history and start to manage the clinical situation.) Each learner should also perform a physical exam. | | |
| **Actors and roles in the room at case start**  *Who is present at the beginning and what is their role? Who may play them?* | A. Roles and who plays them:   - - 1. New Hire Nurse. Provides initial history and explains, “ I am new here so I don't know very much”. This will prompt the learner to be ready to explain clinical reasoning. The nurse is played by one of the simulation center staff, however can be played by RN, resident or MD.     2. Attending physician. The learner gives handoff to the attending physician at the end of the scenario. The attending then provides best practice role modeling for interacting with the patient, which may include discussion of diagnosis, obtaining informed consent, and counseling. This role is usually played by one of the faculty, but could also be played by a resident.     3. Patient. The mannequin responds to questions from the learner and provides history. The voice of the mannequin can be played by anyone familiar with the scenario and does not require the individual to have medical knowledge. Supervising faculty can direct the patient's responses as needed if unexpected questions arise.   B. Behind the scenes personnel to monitor scenario from behind one-way glass, or via video.  1. At least one faculty observer to perform assessment and provide feedback during debriefs. This person may have direct communication with New Hire Nurse.  2. Voice for mannequin- may be performed by personnel monitoring vital signs or by second person.  3. Monitor: this person must be able to communicate directly with New Hire Nurse to give instructions as the scenario unfolds; this is best accomplished by headphone- earpiece, but could be done by text message or display on mobile device screen (the latter will slow down the role-play and decrease believability of simulation). | | |
| **HPI**  *Please specify what info here and below must be asked vs what is volunteered by patient or other participants* | Scenario Background Given to Participants  1. Chief complaint: given to first Intern by New Hire Nurse as they enter the room using SBAR technique.  “Susan is a 31 year old G2P0SAb1 female with LMP 10 weeks ago, came in this morning complaining of bright red bleeding. She has no drug allergies and takes prenatal vitamins and iron.  “She was brought in by her boyfriend, who left and seemed angry. I don't have much more history than that.  “I think she has lost a lot of blood, she has gone through 2 pads since she got here. I brought her right in.  “I started an IV and drew a set of labs. I wasn't sure what you might want. Let me know what you want sent.  “Do you have any questions for me?”  The New Hire Nurse should explain s/he is new here and for the intern to speak loudly as s/he has a hearing problem (this allows observers to be able to hear interactions more clearly).  The remainder of the history is not supplied to the learner unless elicited from the patient. The patient will give only information that is requested. | | |
| **Past Medical/Surgical History** | **Medications** | **Allergies** | **Family History/Social** |
| Must be asked for  Anemia  Fibroids  Prior spontaneous abortion at 8 weeks requiring D&C  Asthma as a child  No abnormal pap tests, no STI, no significant gynecological history | Given in New Hire Nurse handoff  Prenatal vitamins  Iron supplement started after first prenatal visit | Given in New Hire Nurse handoff  No Known Drug Allergies | Must be asked for  Patient's boyfriend waiting outside; he is a smoker.  Patient denies tobacco or drug use, drank 7-10 alcoholic beverages per week before pregnancy, now stopped.  Somewhat evasive when asked about domestic violence, admits to partner yelling, does not directly answer about violence.  Works as a secretary lives with boyfriend who is unemployed.  No significant family history |
| **Physical Examination** | | | |
| **General** | Pale young woman, appears anxious. | | |
| **HEENT** | unremarkable | | |
| **Neck** | No thyromegaly noted | | |
| **Lungs** | clear to auscultation | | |
| **Cardiovascular** | regular rate and rhythm, no murmur noted | | |
| **Abdomen** | Abdomen: soft, fundus palpable to the umbilicus, mildly tender above  Symphysis, no costo-vertebral angle tenderness | | |
| **Neurological** |  | | |
| **Skin** | pale | | |
| **GU** | Normal external female genitalia  Blood at the introitus  If the learner requests to perform a pelvic exam, the New Hire Nurse can explain “I will have to get ready for that”. The First Intern is not expected to perform the pelvic exam, and should tell the Second Intern in the handoff that the internal pelvic exam has yet to be done. | | |
| **Psychiatric** |  | | |

| Instructor Notes - Changes and CASE Branch Points Version one: multiple learners | | | |  |
| --- | --- | --- | --- | --- |
| **Intervention / Time point** | **Change in Case** | **Additional Information** | |  |
| Intern introduces self to patient and obtains history. | . | The patient answers all questions as asked, but does not volunteer any information.  Patient is complaining of lightheadedness, “ I feel really bad”, “I'm so dizzy”. | |  |
| Intern asks to perform physical exam | Initial Vital signs:  Blood Pressure 120/70; Heart Rate100; Respirations 18; Temp 37.0; O2 saturation 99%  Heart exam: regular rate and rhythm, no murmur noted  Lung exam: clear to auscultation  Abdomen: soft, fundus palpable to the umbilicus, mildly tender above  Symphysis, no costo-vertebral angle tenderness  Genitalia: Normal external female genitalia  Blood at the introitus | Can be given on index card for low-fidelity model*.* | |  |
|  |  |  | |  |
| Intern asks for labs | If History and physical exam are not done yet: | New Hire Nurse responds” Those were ordered, and we’re waiting for the results”. | |  |
| Intern asks for labs | If History and physical exam are completed: | New Hire Nurse gives results of labs. (Printed on index card).  Complete Blood Count: White Blood Cells 14.6K, Hemoglobin 5.3, Hematocrit 15.7%,  Platelets 147, 000  Coagulation Studies: PT/PTT 11.9/34 INR 1.8  Fibrinogen: 187  Blood Type O negative | |  |
| Intern asks for ultrasound. | If History and physical exam are not done yet: | New Hire Nurse informs Intern “That was ordered, we’re waiting for the results”. First Intern dose not get results during his/her role play. | |  |
| Intern orders CT, MRI or other clearly not indicated test. |  | | New Hire Nurse can challenge the order: “are you sure you want to order that?”, or “ can you tell me why you want that?” |  |
| Intern asks for ultrasound. | If History and physical exam are completed: | Call into room with ultrasound (Results are found in Appendix F). | |  |
| 4 minutes into case, Patient states “ I think I am bleeding more again” | New Hire Nurse shows Intern chux and pads with increasing blood. | Blood can be hidden in a 60 mL syringe in the bed linens and passed by New Hire Nurse without learner noticing. | |  |
| 5 minutes into case | Vital signs deteriorate..  BP 100/60 Heart Rate 110, Respirations 18 O2 saturation 99% | New Hire Nurse can alert Intern is Intern does not notice change. | |  |
| Intern asks for IV fluids, O2, patient position change |  | Vital signs briefly improve but begin to decrease again within 1 minute. | |  |
| 5-7 minutes into case | First Intern is paged out of simulation | This may be by phone call, overhand page, or other means. | |  |
| First Intern gives handoff to Second Intern |  | Second Intern should have opportunity to ask questions. Once handoff is complete, new Hire Nurse escorts First Intern out, saying s/he is needed in the OR. | |  |
| Second Intern asks to do pelvic exam | Pelvic:  Normal external female genitalia, no trauma or lesions  Blood at the introitus  100 mL blood clot in the vaginal vault  Cervix open, active bleeding from the os  Uterus enlarged approximately to 14 weeks’ size, irregular contour  Ovaries non palpable | Can be given on index card for low-fidelity model*.* | |  |
| 7-10 minutes into case | | Changes in patient's condition  Repeat Vital Signs  BP106/70 HR 110  BP 85/56 HR 130  i |  | |
| 10-12 minutes into case | | Second Intern is paged out of simulation | This may be by phone call, overhand page, or other means. | |
| Second Intern gives handoff to Third Intern |  | Third Intern should have opportunity to ask questions. Once handoff is complete, new Hire Nurse escorts Second Intern out, saying s/he is needed in the OR. | |  |
| Third Intern introduces him/herself and reviews the history with the patient. | . | The patient answers all questions as asked, but does not volunteer any information.  Patient is complaining of lightheadedness, “ I feel really bad”, “I'm so dizzy”. | |  |
| Third Intern asks to repeat exam. | Vital Signs  BP 106/70, Heart Rate 110 bpm  Repeated  BP 85/56, Heart Rate 130bpm  Abdomen: soft, tender over fundus, no rebound or guarding  Pelvic: large blood clot 500mL in vaginal vault  Cervix: apparent fetal membranes visible at os |  | |  |
| Third Intern asks for ultrasound results. |  | Call into room with ultrasound (Results are found in Appendix F). | |  |
| Patient asks if her doctor is available. |  | New Hire Nurse pages Attending. | |  |
| Third intern determines need for surgery | Attending arrives |  | |  |
| Third Intern gives handoff to Attending. |  |  | |  |
| Attending informs patient of diagnosis and need for surgical intervention. Attending asks New Hire Nurse to notify OR of need for urgent D&C. |  | Any unaddressed clinical findings requiring interventions can be ordered by Attending ( transfusions, fluids, oxygen). | |  |

| Instructor Notes - Changes and CASE Branch Points Version one: single learner | | | |  |
| --- | --- | --- | --- | --- |
| **Intervention / Time point** | **Change in Case** | **Additional Information** | |  |
| Intern introduces self to patient and obtains history. | . | The patient answers all questions as asked, but does not volunteer any information.  Patient is complaining of lightheadedness, “ I feel really bad”, “I'm so dizzy”. | |  |
| Intern asks to perform physical exam | Initial Vital signs:  Blood Pressure 120/70; Heart Rate100; Respirations 18; Temp 37.0; O2 saturation 99%  Heart exam: regular rate and rhythm, no murmur noted  Lung exam: clear to auscultation  Abdomen: soft, fundus palpable to the umbilicus, mildly tender above  Symphysis, no costo-vertebral angle tenderness  Genitalia: Normal external female genitalia  Blood at the introitus | Can be given on index card for low-fidelity model*.* | |  |
| Intern asks to do pelvic exam | Pelvic:  Normal external female genitalia, no trauma or lesions  Blood at the introitus  100 mL blood clot in the vaginal vault  Cervix open, active bleeding from the os  Uterus enlarged approximately to 14 weeks’ size, irregular contour  Ovaries non palpable | Can be given on index card for low-fidelity model*.* | |  |
|  |  |  | |  |
| Intern asks for labs | If History and physical exam are not done yet: | New Hire Nurse responds” Those were ordered, and we’re waiting for the results”. | |  |
| Intern asks for labs | If History and physical exam are completed: | New Hire Nurse gives results of labs. (Printed on index card).  Complete Blood Count: White Blood Cells 14.6K, Hemoglobin 5.3, Hematocrit 15.7%,  Platelets 147, 000  Coagulation Studies: PT/PTT 11.9/34 INR 1.8  Fibrinogen: 187  Blood Type O negative | |  |
| Intern asks for ultrasound. | If History and physical exam are not done yet: | New Hire Nurse informs Intern “That was ordered, we’re waiting for the results”. First Intern dose not get results during his/her role play. | |  |
| Intern orders CT, MRI or other clearly not indicated test. |  | | New Hire Nurse can challenge the order: “are you sure you want to order that?”, or “ can you tell me why you want that?” |  |
| 4 minutes into case, Patient states “ I think I am bleeding more again” | New Hire Nurse shows Intern chux and pads with increasing blood. | Blood can be hidden in a 60 mL syringe in the bed linens and passed by New Hire Nurse without learner noticing. | |  |
| 5 minutes into case | Vital signs deteriorate..  BP 100/60 Heart Rate 110, Respirations 18 O2 saturation 99% | New Hire Nurse can alert Intern is Intern does not notice change. | |  |
| Intern asks for ultrasound. | If History and physical exam are completed *and* at least 6 minutes of simulation are completed. | Call into room with ultrasound (Results are found in Appendix F). | |  |
| Intern asks for IV fluids, O2, patient position change |  | Vital signs briefly improve but begin to decrease again within 1 minute. | |  |
| 7-10 minutes into case | | Changes in patient's condition  Repeat Vital Signs  BP106/70 HR 110  BP 85/56 HR 130  i |  | |
| Intern asks to repeat exam. | Vital Signs  BP 106/70, Heart Rate 110 bpm  Repeated  BP 85/56, Heart Rate 130bpm  Abdomen: soft, tender over fundus, no rebound or guarding  Pelvic: large blood clot 500mL in vaginal vault  Cervix: apparent fetal membranes visible at os |  | |  |
| Patient asks if her doctor is available. |  | New Hire Nurse pages Attending. | |  |
| Intern determines need for surgery | Attending arrives |  | |  |
| Intern gives handoff to Attending. |  |  | |  |
| Attending informs patient of diagnosis and need for surgical intervention. Attending asks New Hire Nurse to notify OR of need for urgent D&C. |  | Any unaddressed clinical findings requiring interventions can be ordered by Attending ( transfusions, fluids, oxygen). | |  |

Ideal Scenario Flow

Each learner walks into room with the New Hire Nurse, who explains s/he is new, and would like to hear everything the learner says, “to learn more”. The New Hire Nurse introduces each learner to Susan, a 31 year old, G2P0 SAB1 female who presented this morning with vaginal bleeding, and who is 10 weeks pregnant. The New Hire Nurse informs the First Intern that the New Hire Nurse started an IV and drew “some blood”.

Each learner introduces him/herself to the patient and is expected to take a history. There are accurate, real-time vital signs present on the bedside monitor. There is an IV set up, O2 available, and a code cart. The learner can write down history and findings on paper, electronic device or wallboard. (In our experience the first learner to enter the scenario usually has adequate time to obtain a history and start to manage the clinical situation.)

The learner can perform a physical exam on the mannequin including external genital exam; internal pelvic exam findings are provided on index cards. Each learner should inspect the external genitalia to assess the quantity of bleeding and be able to give an estimate. Each learner should verify the clinical findings as these findings change during the scenario. The learner s can be instructed prior to the role- play that they are able to perform a physical exam, but that the findings will be given to them on index cards to ensure uniformity. This instruction helps the learners to suspend disbelief during the role-play, and can help lessen the anxiety for learners.

The learner can ask for labs and ultrasound. If these have not been ordered earlier in the scenario, the learner is informed, “these will take a few minutes”. The learner can be given the results up to 1 minute prior to the end of their allotted time in the role-play, so that there is adequate time (at least one minute) to interpret the results prior to the handoff. If there is not enough time remaining, the learner is expected to explain in the handoff which results are pending. The results can then be given to the next learner by the New Hire Nurse when asked.

Each learner is able to order IV fluids, O2, patient position change, blood transfusion, and medications. The New Hire Nurse should complete the orders requested if they are appropriate interventions; the supervising faculty can indicate to the New Hire Nurse to suggest alternative interventions if the requested treatment is inappropriate, or to ask the learner, “ are you sure?' or “ can you tell me why we want to do/give that”.

Each learner should spend 5 minutes obtaining or completing the history and exam, and should be provided with any results s/he has requested with 1-2 minutes left in the scenario. Each learner should be instructed that the exercise will end with a phone call into the room where the learner is asked to come assist in the OR and informed another intern will relieve the learner.

When the next learner arrives, the outgoing learner gives a handoff using SBAR technique. The incoming learner should have the opportunity to ask questions.

A. Scenario Background Given to Participants

1. Chief complaint: given to first Intern by New Hire Nurse as they enter the room using SBAR technique.

“Susan is a 31 year old G2P0SAb1 female with LMP 10 weeks ago, came in this morning complaining of bright red bleeding. She has no drug allergies and takes prenatal vitamins and iron.

“She was brought in by her boyfriend, who left and seemed angry. I don't have much more history than that.

“I think she has lost a lot of blood, she has gone through 2 pads since she got here. I brought her right in.

“I started an IV and drew a set of labs. I wasn't sure what you might want. Let me know what you want sent.

“Do you have any questions for me?”

The New Hire Nurse should explain s/he is new here and for the intern to speak loudly as s/he has a hearing problem (this allows observers to be able to hear interactions more clearly).

The remainder of the history is not supplied to the learner unless elicited from the patient.

2. Past medical history (must be asked for)

Anemia

Fibroids

Prior spontaneous abortion at 8 weeks requiring D&C

Asthma as a child

No abnormal pap tests, no STI, no significant gynecological history

3. Meds and allergies (given in handoff from New Hire Nurse)

Prenatal vitamins

Iron supplement started after first prenatal visit

4. Family/social history (must be asked for)

Patient's boyfriend waiting outside; he is a smoker.

Patient denies tobacco or drug use, drank 7-10 alcoholic beverages per week before pregnancy, now stopped.

Somewhat evasive when asked about domestic violence, admits to partner yelling, does not directly answer about violence.

Works as a secretary lives with boyfriend who is unemployed.

No significant family history

B. Initial Scenario conditions

- - - - 1. History (elicited from patient)

Patient is reticent and only gives history in response to questions. Of note, the patient only gives history asked for from any of the learners and does not volunteer information. (If a learner is struggling, the Monitor or Faculty can prompt the patient to give more information.) The actor playing the patient can adapt the following story line:

Patient states she woke up this morning and went to the bathroom, where she noticed some cramping and blood in the toilet bowl. When asked she is unable to quantify but thinks it was a lot of blood. She is concerned as this happened last year when she had a miscarriage. She woke up her boyfriend “who came in really late last night” and asked him to bring her to the Emergency Room.

When asked if she has had prenatal care yet, she answers yes, she was seen 2 weeks ago at her obstetrician's office where an ultrasound was done that showed her to be 8 weeks along. If asked she states this did confirm her due date. She adds that the doctor said she has something else in the uterus, maybe a fibroid. She also notes the doctor started her on iron, due to some anemia. This has been a problem for the patient her whole life.

The patient gives her past medical history when asked.

The patient answers evasively any questions about her relationship, domestic violence, and social history. When asked about the boyfriend, she asks if her boyfriend is waiting, and if asked, states she does not want him to come into the room. She is vague in answering questions about family history, stating her family “doesn't talk about medical stuff”.

2. Patient's initial exam

When the first intern tells the New Hire Nurse and the patient that s/he would like to do a physical exam, the following findings are encountered. If a programmable mannequin is used these initial findings should be entered; the index card will also list the findings, and the index card can be handed to the learner in a low-tech simulation session.

If the learner requests to perform a pelvic exam, the New Hire Nurse can explain “I will have to get ready for that”. The First Intern is not expected to perform the pelvic exam, and should tell the Second Intern in the handoff that the internal pelvic exam has yet to be done.

3. Management

At any point the learner may order laboratory tests, order fluids or other medications, order imaging studies. If the learner has not ordered any tests or interventions by 5 minutes, the New Hire Nurse can suggest these orders. If a CT, MRI or other clearly not indicated test is ordered, the New Hire Nurse can challenge the order: “are you sure you want to order that?”

4. Handoff

The First Intern is now called out of the scenario: a phone call into the room occurs, answered by the Nurse, who informs the First Intern s/he is needed in the OR, but that another intern will be coming to take over care. Second Intern now enters the scene. First Intern gives handoff to Second Intern using SBAR technique, allowing the Second Intern to ask questions. The First Intern should explain s/he was called away before a pelvic exam could be done, and that the Second Intern should complete the exam. The First Intern should specify what tests and interventions have been ordered.

C. Scenario branch point: Second Intern

Interval History: The Second Intern introduces him/herself and reviews the history with the patient. The patient answers all questions as asked, but again does not volunteer any information. Depending on the level of detail of the handoff, the Second Intern may need to gather more history from the patient.

- - - - 1. Changes in patient's condition

a. Vital signs trend toward hypotension and tachycardia. These can be entered into the monitors from the observation booth, or can be voiced by the New Hire Nurse in a low-tech setting. If the learner fails to notice the change, the New Hire Nurse can say: “ Her blood pressure seems to be dropping. Would you like me to do anything?”

b. Patient complains of fatigue, lightheadedness.

c. Physical exam can be repeated with no change other than vital signs and tachycardia.

d. Pelvic exam findings: these are given to the learner on index card 3 by the New Hire Nurse (this portion of the scenario requires suspension of disbelief on the part of the learner).

Card 3: Pelvic Exam

Pelvic:

Normal external female genitalia, no trauma or lesions

Blood at the introitus

100 mL blood clot in the vaginal vault

Cervix open, active bleeding from the os

Uterus enlarged approximately to 14 weeks’ size, irregular contour

Ovaries non palpable

Repeat Vital Signs: BP 100/60 Heart Rate 110, Respirations 18 O2 saturation 99%

2. Management

At any point the learner may order laboratory tests, order fluids or other medications, order imaging studies. If the learner has not ordered any tests or interventions by 5 minutes, the New Hire Nurse can suggest these orders. If a CT, MRI or other clearly not indicated test is ordered, the New Hire Nurse can challenge the order: “are you sure you want to order that?”

1. Results

Within 4 minutes of the role-play, the New Hire Nurse should give the learner the results of any tests ordered (index card 4) except the ultrasound, which should be reserved for the last learner, or end of the scenario.

Card 4 Laboratory Results

Patient’s boyfriend is waiting outside, asking what is going on

Laboratory Results:

Complete Blood Count: White Blood Cells 14.6K, Hemoglobin 5.3, Hematocrit 15.7%, Platelets 147, 000

Coagulation Studies: PT/PTT 11.9/34 INR 1.8

Fibrinogen: 187

Blood Type O negative

The learner may react with orders for interventions, or may not have time to do so. The learner should include this in the handoff.

1. Handoff

The Second Intern is now called out (phone call into scenario, answered and related to Second Intern by Nurse) and the Third Intern enters the scene. The handoff takes place, using SBAR technique and allowing the incoming intern to ask questions. The Second Intern should explain s/he was called away before the ultrasound results are back, that there appears to be more bleeding and that the incoming intern should repeat the pelvic exam. The Second Intern should specify what tests and interventions have been ordered and are pending, and give an interpretation of the results received so far.

D. Scenario branch point: Third Intern

Interval History: The Third Intern now introduces him/herself and reviews the history with the patient. The patient answers all questions as asked, but again does not volunteer any information.

1. Changes in patient's condition

Vital signs continue to trend toward hypotension and tachycardia.

Patient is complaining of lightheadedness, “ I feel really bad”, “I'm so dizzy”

Repeat Physical Exam: Card 5 (handed to Third Intern by Nurse)

Repeat Vital Signs

BP106/70 HR 110

BP 85/56 HR 130

Abdomen: soft, tender over fundus, no rebound or guarding

Pelvic: large blood clot 500mL in vaginal vault

Cervix: apparent fetal membranes visible at os

2. Management

At any point the learner can order laboratory tests, order fluids or other medications, order imaging studies. If a CT, MRI or other clearly not indicated test is ordered, the New Hire Nurse can challenge the order: “are you sure you want to order that?

The ultrasound report can be called into the room, or can be given on paper.

1. Results

Within 4 minutes of starting this portion of the role-play, the New Hire Nurse should give the Third Intern the results of any tests ordered and not previously received via handoff. When the Third Intern receives the results of the ultrasound, the New Hire Nurse should ask the Third Intern if s/he needs to notify anyone. The Third Intern is given Card 6 at this point.

Card 6 Hospital Systems Information

OR: extension 72411

Attending: extension 72295

Code Blue: extension 21212

Blood Bank: extension 72205

1. The Faculty Observer can intervene if it appears the Third Intern feels s/he must inform the patient of the results.
2. The Third Intern should recognize need to notify the OR, the Attending and the Blood Bank and should state this to the New Hire Nurse (if not prompting can be done). The Third Intern is then called out of the role-play without giving the interpretation of the ultrasound to the patient. (In the advanced scenario the learner gives the results to the patient.)
3. Handoff

The Third Intern is now called out for surgery and the Attending physician enters the scenario. The handoff takes place, using SBAR technique and allowing the Attending to ask questions for clarification. The Third Intern should explain s/he was called away before the results of the ultrasound have been shared with the patient. The Third Intern should specify what tests and interventions have been ordered, what are significant results, and patient's physiologic response to interventions.

E. Faculty Role Play

The Attending who enters the scene and takes the last handoff can model best practice behavior with interviewing the patient, explaining the results and recommendation for surgical management. Addressing psychosocial needs of the patient should be included (asking if patient has a supportive person with her, assuring the patient she did nothing to cause the miscarriage, asking if any phone calls to family or friends are needed). A demonstration of obtaining informed consent can occur if there is sufficient time.

VII. Instructor’s Notes (what the instructor must do to create the experience)

A. Tips to keep scenario flowing in lab

-Helpful to have a headset for communication from observers to the New Hire Nurse in the scenario. Texting via smartphone or mobile device can work but is slower.

- Ability to call in to room (with ultrasound preliminary report and to page the learner out to signify thee end of the scene) helpful but could be replaced with paging.

B. Tips to direct actors

- - - New Hire Nurse should help learner to identify increasing blood loss. Our system allows the New Hire Nurse to increase the amount of bleeding with pushing a syringe located under the bed linens.
    - Have simulation IV fluids and packed red blood cells in an anteroom or adjacent space to the room.

C. Scenario programming

1. Optimal management path

Intern Actions

Takes history

Interprets vital signs and lab results

Orders fluid resuscitation and O2

Orders blood products

Interprets physical findings and ultrasound results

Handoff patient to next provider

2.Optimal Sequence of Intern Actions:

Take History

Interpret vital signs

Order fluids, imaging and labs

Perform physical exam

Interpret physical findings and labs

Order ultrasound

Order blood products and manage fluids

Interpret ultrasound

(Inform patient of diagnosis and recommendations for surgery- not for clerkship student level learner)

Handoff patient to another provider

Anticipated Management Mistakes

- 1. Misinterpretation of Results: Occasionally the learner misinterprets results or orders unnecessary interventions. This can lead to a delay in diagnosis of acute blood loss. The New Hire Nurse, directed via headset- earpiece if needed by the Faculty Observer, can address these complications. However, this mistake will not affect the handoff. The incoming Intern should either have the opportunity to ask for clarification from the leaving Intern, or should re-examine the results and arrive at the correct interpretation.
  2. Leaner Stress: A learner may become overwhelmed by the role-play. Although we have not had this happen in our experience, learners should be reminded at the start of the session that this is not part of their overall grade in the clerkship, and that this is a safe place to practice handoff skills and patient management skills. If a learner appears to be overwhelmed the scenario can be stopped at any point. The Debrief checklist addresses learner well-being and stress.
  3. Failure to Start Interventions: A learner may fail to perform a physical exam, start IV fluid resuscitation, order labs, or order blood transfusion. This can lead to delay in diagnosis or treatment. The New Hire Nurse can suggest interventions if the learner does not recognize the need.
  4. Pertinent Positives. The history contains details which might distract the learner, for example suggestions of intimate partner violence. The learner is expected to discern the urgent nature of the patient’s condition. An excellent learner will note these concerns and the need to follow up on this, but will not be distracted from the urgent situation at hand.

Debriefing Plan

1. Method of debriefing
   - - - 1. Third Year Student Version: the 3 or 4 students who participated in one run of the role-play are assembled and debriefing occurs as a group with Faculty observers. Video can be used to review if available. Faculty observers use debrief tool to address topics.
         2. Fourth Year Student Version: each student has an individual debrief with a Faculty observer. Video can be used if available. Faculty observers use debrief tool to address topics.
2. Debriefing materials

Debrief checklist is detailed in Appendix H.

C. Rules for the debriefing

- - - - 1. Check in with learners emotionally.
        2. Reassure students regarding management decisions. Correct mistakes made.
        3. Emphasize this is a chance to practice handoff and clinical management skills.
        4. Emphasize this is not used in assessment or evaluation.

D. Questions to facilitate the debriefing

- - - 1. When you gave the handoff, did you use the SBAR technique? If not, why not? Would SBAR have helped you to be more complete in your handoff?
      2. When you received the handoff did you remember to ask questions and clarify? If not, why not?
      3. Was closed-loop communication used in the handoffs?
      4. What could have gone better?
      5. How would you improve the scenario?
      6. Review the clinical findings and explain each significant item:
         1. Pre-existing anemia contributes to patient's rapid decline.
         2. Enlarged uterus is due to fibroids, but may be due to multiple gestations, molar pregnancy.
         3. The learners may have been distracted be the social history, suggestive of ongoing domestic violence issues, or the possibility of recurrent pregnancy loss. The learners should recognize the need for emergency surgery and not be distracted by non-urgent findings.
         4. Some findings, which may seem incidental are significant, for example the Rh-negative status indicates need for rhogam.
         5. Pelvic exam findings with open cervical os and tissue protruding indicate need for surgical management.
         6. Rapid decompensation indicates need for surgical management.

E. Evaluation form for participants

1. A Student Assessment form is included (Appendix G, Student Assessment Form).
2. Survey Monkey or other clerkship evaluation tool can be administered.
