# Supplementary material for: Teaching Patient Handoffs to Medical Students in Obstetrics and Gynecology: Simulation Curriculum and Assessment Tool
Source: MedEdPORTAL. 2016 Oct 2;12:10479. doi: 10.15766/mep_2374-8265.10479 (PMC6440488; doi:10.15766/mep_2374-8265.10479)
Supplement: Supplementary file 1 — A. Patient Handoffs in Obstetrics and Gynecology.pptx B. Approach to Diagnosis and Management of First Trimester Bleeding.pptx C. Patient Handoffs in Obstetrics and Gynecology Narrated.mp4 D. Approach to Diagnosis and Management of First Trimester Bleeding Narrated.mp4 E. Handoff Skills Speakers Notes.docx F. First Trimester Bleeding Speakers Notes.docx G. Simulation Guide.docx H. Role Play Description.docx I. Trainee Simulation Information Cards.doc J. Ultrasound Report.docx K. Student Assessment Tool.docx L. Debrief Checklists.docx [file mep-12-10479-s001.zip › H. Role Play Description.docx]

**Teaching Patient Handoffs to Medical Students in Obstetrics and Gynecology: A Simulation Curriculum and Assessment Tool**

**Appendix H: Role Play Guide**

Simulation exercises and workshops provide a safe learning environment, giving participants the opportunity to practice skills and behaviors without worry for patient harm or other adverse events. One area, which is notoriously difficult to learn, and which is of utmost importance is the safe handoff of patients from one caregiver to the next. The simulation lab is an ideal environment to practice this skill, using a partially scripted scenario involving role-playing by trainees. We intend for students to practice team building and leadership skills, as well as the management of gynecological hemorrhage in emergency settings.

Our simulation is run with the Simulation Center Staff. One staff member functions as the "registered nurse" in the scenario, and another manages the mannequin and vital signs, adjusting the status of the patient during the scene. Two didactic sessions precede the simulation: one reviewing handoffs using the “SBAR” system, and the second reviewing the diagnosis, evaluation and management of bleeding in the first trimester of pregnancy. The simulation consists of a 10-minute role-play followed by a debrief session.

The students meet with one of the faculty for two introductory sessions, during which patient handoffs skills and a short didactic on the evaluation and management of first trimester bleeding are presented. Topics discussed include: taking a history, pertinent physical findings to evaluate, and determining which imaging and lab studies are appropriate to order. The students are informed they will be managing a patient as they will on the wards later in their careers as residents, and to order labs, tests and treatment interventions as they think appropriate. For clerkship students, we emphasize that during the simulation they are expected to evaluate and manage the patient, but that they are not expected to inform the patient of a diagnosis, as that will be done at the conclusion of the scenario as a team. For boot camp students and intern orientation, the instructions include counseling the patient regarding her diagnosis and obtaining informed consent for a dilation and curettage (D & C). We explicitly acknowledge that simulation exercises are difficult, requiring suspension of disbelief, and can be intimidating, challenging and potentially frightening. We also explicitly state the exercise is not used in their course evaluation.

**Clerkship Version**

Learners are introduced to the scenario at the conclusion of the didactic sessions. Learners are given the case scenario below, and instructed that each learner will have the opportunity to receive and give a handoff. Learners participate in groups of 3. Learners can choose to be the First Intern, the Second Intern, or the Third Intern (the scenario can be adapted for a fourth learner). The First Intern receives a handoff from the RN actor, and the Third (or Fourth) Intern gives a handoff to the attending physician. All other handoffs are with other learners, and all participants should be assured they all will have the opportunity to give and receive a handoff.

Learners remain in an anteroom or other adjacent room until they are called into the scenario.

Learners are instructed to take a history from the patient, and that the patient will give answers to any questions asked, but will not volunteer any information. The RN actor will also answer any questions asked, but is a “newly hired RN”, who may need some time to supply the answers. Learners may give verbal orders to the RN, and are expected to evaluate the patient, order interventions (IV fluids, oxygen, medications, blood), and request lab and imaging results. Learners are instructed to perform as much of a physical exam as they would like to do, but that findings may not be available at some points in the scenario. This is also true of imaging and lab results. The learner should manage the patient based on the information available at the time. The learner can take notes on paper, a hand held device, or a wall-mounted erasable board. (Learners do not have to take notes, but our experience indicates a higher comfort level for learners who do take notes.)

Learners should be told their time in the scenario will be approximately 5-7 minutes, and at the end of the allotted time, their character will be paged out of the scenario, and the next intern will be sent in (for the last intern, this will be the attending physician). The learner should then give a handoff to the incoming intern or attending, using the SBAR technique.

Once the learner leaves the scenario, s/he waits in a de-brief room. Learners may wish to observe the rest of the scenario, if possible.

Once the last learner has given a handoff to the attending, the attending calls the team back to the room. The attending reviews the actions thus far, making sure clinical care has been appropriate, models professionalism and humanism while interacting with the patient, demonstrates how to break bad news compassionately, and how to quickly obtain informed consent for a procedure under urgent circumstances. Additionally, the attending can address psychosocial aspects of the case, which are sometimes neglected by the students.

**Case Scenario: First Trimester Bleeding**

Susan Quinn is a 30 yo G2P0 SAb1 female, with a last menstrual period 10 weeks prior to presentation. Ms. Quinn has a history of fibroids and chronic anemia, attributed related to heavy menstrual bleeding. She presents to the Emergency Department complaining of bleeding since 6 am this morning. Ms. Quinn states that after waking up, she went to urinate and noticed she was cramping. She states, “the toilet was full of blood”, and she called her boyfriend Charles, who brought her to the ED. The patient states her primary obstetrician saw her 2 weeks prior to presentation for prenatal care, at which time an ultrasound confirmed a singleton, intrauterine pregnancy of 8 weeks. The patient denies other symptoms except cramping abdominal pain, which is getting worse. She had a prior first trimester pregnancy loss 2 years ago that did not require a D&C, and has been trying to conceive since then. She has no prior surgical or other medical history. She is a former smoker, denies alcohol or drug use, has no pets or recent travel history, and lives with her boyfriend who does smoke. Ms. Quinn describes their relationship is “rocky”, but is vague when asked regarding controlling or violent behavior. She has a sedentary clerical job. Family history is non-contributory, with both parents living, and no family history of recurrent pregnancy loss. Her review of systems is otherwise negative.

This history is intended to be elicited by students during the exercise, and thus is not revealed to them. There are historical and physical findings, which are intended to provoke a robust differential diagnosis (chronic anemia, prior spontaneous abortion, uterus much larger than expected) as well as to rule out potential causes for first trimester bleeding (known intra-uterine pregnancy on early ultrasound precludes twins, molar pregnancy, etc.; fibroids are the intended explanation for the large uterus).

The exercise asks each student to play the role of an OBGYN intern. Each learner is expected to enter the patient's room, receive a handoff from the personnel present (either the RN for the first student or another learner), interact with the patient, and formulate a management plan. The learner is then called away, and is expected to handoff the patient to the incoming intern. The last student is expected to give a handoff to the faculty attending.

The " intern" is asked by the RN to come evaluate a patient. The first intern is given the following information initially:

**CARD 1**

DOOR CHART INFORMATION

30 yo G2 P0 with LMP 10 weeks ago, bleeding since this morning

Medications: prenatal vitamins, iron

NKDA

The intern then interacts with the mannequin, who is voiced by a faculty member, to elicit a history. The intern can give orders to the RN for fluids, labs and imaging studies as needed. The RN, prompted by a faculty member via head set, alerts the trainee as to any clinical information or changes that the trainee may be missing. Once the intern has obtained a history, s/he receives a second card, with the following physical findings:

**CARD 2**

BRIEF PHYSICAL EXAMINATION

Initial Vital signs:

120/70; 100; 18; 37.0; O2 sat 99%

Heart exam: regular rate and rhythm, no murmur noted

Lung exam: clear to auscultation

Abdomen: soft, fundus palpable to the umbilicus, mildly tender above

symphysis, no costo-vertebral angle tenderness

Genitalia: Normal external female genitalia

Blood at the introitus

An exam can be done, which should include an estimate of the blood loss. A phone call then is made into the scene, to page the intern out of the scenario. The RN takes the call, informs the intern that s/he is needed in the OR, and that another intern will come to relieve the current intern.

The second intern arrives, and the first intern is expected to give a handoff, using SBAR. The oncoming intern should have opportunity to ask questions and receive clear communication of all the information the first intern has before the first intern is called away.

The second intern is then expected to go through the history with the patient, again asking for tests and treatments as indicated. Once this is completed the a card with pelvic exam findings is given:

**CARD 3**

PELVIC EXAM RESULTS

Pelvic:

Normal external female genitalia, no trauma or lesions

Blood at the introitus

100 mL blood clot in the vaginal vault

Cervix open, active bleeding from the os

Uterus enlarged approximately to 14 weeks’ size, irregular contour

Ovaries non palpable

The patient’s vital signs are trending down, and the student may or may not notice this. The student is given a chance to determine the patient's diagnosis and the next step in the workup and treatment. The second index card is then given, which includes significantly abnormal lab findings:

**CARD 4**

Partner is waiting outside, asking what is going on

Labs: CBC: WBC 14.6, Hgb 5.3, Hct 15.7, Plts 147k

PT/PTT 11.9/34 INR 1.8

Fibrinogen 187

The scenario is again interrupted by a call for the intern to go to the OR. The third intern then enters the scenario. The second intern gives a handoff to the incoming intern. This learner is again intended to review the history with the patient, and perform a physical. The information for the exam at this point is given as:

**CARD 5**

Repeat Physical Exam

Orthostatic Vital Signs

BP106/70 HR 110 supine

BP 85/56 HR 130 sitting

Abdomen: soft, tender over fundus, no rebound or guarding

Pelvic: large blood clot 500mL in vaginal vault

Cervix: apparent fetal membranes visible at os

The students are expected to evaluate the deterioration of the clinical picture and recognize the need for operative management at this point. The RN provides the last intern with a last card, including the OR, Anesthesia, and blood bank phone numbers as well as the code blue page number. The mannequin (voiced by faculty member) is able to direct to some extent the workup and management by asking questions of the caregivers, and the RN can suggest interventions or next steps; the RN is in contact with the faculty member by headset. The students can request an ultrasound, and the results are called into the room by a faculty member. If the students do not interpret the report correctly (i.e., does not recognize the fetal demise), the faculty member calls back with a "final result" and emphasizes the points the students may have missed.

After students leave the patient's room, they are asked to wait in the adjoining de-brief room, where the rest of the case information is made available to them, including an ultrasound report confirming incomplete abortion. The students as a team can then discuss the case and determine the management they would like to recommend.

The last step in the exercise is the arrival of the attending to take handoff from the last intern. The attending is played by another faculty member. The attending comes in to the room (after being paged) and receives report from the "last intern", thus allowing this learner to practice a patient handoff. At this time, the “team” of learners returns to the room. The attending reviews the actions thus far, making sure clinical care has been appropriate, models professionalism and humanism while interacting with the mannequin, demonstrates how to break bad news compassionately, and how to quickly consent a patient for a procedure. Additionally, the attending can address psychosocial aspects of the case, which are often neglected by the students.

At the conclusion, there is a debriefing session with the two faculty members at the end of the session with each set of students. Feedback is elicited from the students about their experience, paying particular attention to feelings of discomfort or student's self-perception of errors, reassuring them this is normal and expected and in fact the purpose of the exercise. We do correct obvious medical errors, but first highlight the positive aspects of their performance. We review how the handoff of information occurred, emphasize what went right and make suggestions for improvement. Lastly, we ask for any helpful criticism they have of the exercise or suggestions for improvement.

**Boot Camp/ Intern Version**

In this version, the learner functions independently throughout the exercise, from receiving the handoff from the triage nurse, through evaluation and management of the patient including informing the patient of the diagnosis and the need for urgent intervention, obtaining informed consent, and giving the handoff to the attending physician. While this version gives the learner a more immersive experience, it does not emphasize team building, and requires more time as each learner will require approximately 15 minutes to complete the role play, and each learner will have an individual de-brief session. Additionally the skills utilized and evaluated are more appropriate for assessing readiness for residency, for example obtaining informed consent.
