# Supplementary material for: Teaching Patient Handoffs to Medical Students in Obstetrics and Gynecology: Simulation Curriculum and Assessment Tool
Source: MedEdPORTAL. 2016 Oct 2;12:10479. doi: 10.15766/mep_2374-8265.10479 (PMC6440488; doi:10.15766/mep_2374-8265.10479)
Supplement: Supplementary file 1 — A. Patient Handoffs in Obstetrics and Gynecology.pptx B. Approach to Diagnosis and Management of First Trimester Bleeding.pptx C. Patient Handoffs in Obstetrics and Gynecology Narrated.mp4 D. Approach to Diagnosis and Management of First Trimester Bleeding Narrated.mp4 E. Handoff Skills Speakers Notes.docx F. First Trimester Bleeding Speakers Notes.docx G. Simulation Guide.docx H. Role Play Description.docx I. Trainee Simulation Information Cards.doc J. Ultrasound Report.docx K. Student Assessment Tool.docx L. Debrief Checklists.docx [file mep-12-10479-s001.zip › J. Ultrasound Report.docx]

**Teaching Patient Handoffs to Medical Students in Obstetrics and Gynecology: A Simulation Curriculum and Assessment Tool**

**Appendix J: Ultrasound Report**

To be read by a facilitator over phone to learner, when requested by the learner.

Facilitator reads:

“*Hello, this is the sonographer calling with a wet read on patient* *Susan M.Quinn, Medical Record: 987-65-432*

*Please remember this is a wet read only. There is a singleton, intrauterine pregnancy with a crown-rump length of 19 mm, consistent with 8 weeks and 5 days. There is no cardiac activity noted, consistent with a fetal demise. The gestational sac appears to be in the lower uterine segment. Several large fibroids are noted, the largest is 5cm and in a fundal location. There are no adnexal masses, no free fluid and the ovaries appear normal.*

.

**Transvaginal Ultrasound “Wet Read” Quick Facts**

Single intrauterine pregnancy

Crown rump length c/w 8w5d

No cardiac activity

Several large fibroids noted

No adnexal masses

No free fluid
