# Supplementary material for: Teaching Patient Handoffs to Medical Students in Obstetrics and Gynecology: Simulation Curriculum and Assessment Tool
Source: MedEdPORTAL. 2016 Oct 2;12:10479. doi: 10.15766/mep_2374-8265.10479 (PMC6440488; doi:10.15766/mep_2374-8265.10479)
Supplement: Supplementary file 1 — A. Patient Handoffs in Obstetrics and Gynecology.pptx B. Approach to Diagnosis and Management of First Trimester Bleeding.pptx C. Patient Handoffs in Obstetrics and Gynecology Narrated.mp4 D. Approach to Diagnosis and Management of First Trimester Bleeding Narrated.mp4 E. Handoff Skills Speakers Notes.docx F. First Trimester Bleeding Speakers Notes.docx G. Simulation Guide.docx H. Role Play Description.docx I. Trainee Simulation Information Cards.doc J. Ultrasound Report.docx K. Student Assessment Tool.docx L. Debrief Checklists.docx [file mep-12-10479-s001.zip › K. Student Assessment Tool.docx]

**Appendix K. Teaching Patient Handoffs to Medical Students in Obstetrics and Gynecology: Student Assessment Tool**

Please score learner’s performance in each professional activity.

| Activity | 1  Consistently Below Expectations | 2  Occasionally Below Expectations | 3  Appropriate to level of training | 4  Often Above  Expectations | 5  Consistently Above Expectations | Not Observed |
| --- | --- | --- | --- | --- | --- | --- |
| 1.History Taking: Student takes complete and accurate history efficiently. Responds to patient's affect and non-verbal cues (EPA 1). |  |  |  |  |  |  |
| 2. Fund of Knowledge: Student applies understanding of pathophysiology to clinical context (EPA 2). |  |  |  |  |  |  |
| 3. Clinical Evaluation and Management: Student demonstrates a well-reasoned, individualized use of tests and procedures, generating a comprehensive treatment plan (EPA 3). |  |  |  |  |  |  |
| 4. Interpersonal Skills: Student engenders trust, and uses communication skills to deliver high level of care. |  |  |  |  |  |  |
| 5. Presentation Skills: Student uses SBAR to present a cogent, efficient and sophisticated hand off, synthesizing complex information, conveying thought processes behind clinical decisions, and tailored to the setting. (EPA 8). |  |  |  |  |  |  |
| 6. Professionalism: Student models highest standards of integrity, reliability and collegiality in interactions with team and patient, seeking out and accepting responsibility |  |  |  |  |  |  |
| 7. Desire to Learn: Student demonstrates intellectual curiosity (EPA 7). |  |  |  |  |  |  |
| 8. Student recognizes critical findings (EPA 10). |  |  |  |  |  |  |
| 9. Student obtains informed consent for D&C (EPA 11). |  |  |  |  |  |  |
| 10. Systems Awareness: Student understands and uses hospital systems information to facilitate patient care (notifies OR, blood bank, anesthesia) (EPA 13). |  |  |  |  |  |  |

**Comments**:
