# Supplementary material for: Teaching Patient Handoffs to Medical Students in Obstetrics and Gynecology: Simulation Curriculum and Assessment Tool
Source: MedEdPORTAL. 2016 Oct 2;12:10479. doi: 10.15766/mep_2374-8265.10479 (PMC6440488; doi:10.15766/mep_2374-8265.10479)
Supplement: Supplementary file 1 — A. Patient Handoffs in Obstetrics and Gynecology.pptx B. Approach to Diagnosis and Management of First Trimester Bleeding.pptx C. Patient Handoffs in Obstetrics and Gynecology Narrated.mp4 D. Approach to Diagnosis and Management of First Trimester Bleeding Narrated.mp4 E. Handoff Skills Speakers Notes.docx F. First Trimester Bleeding Speakers Notes.docx G. Simulation Guide.docx H. Role Play Description.docx I. Trainee Simulation Information Cards.doc J. Ultrasound Report.docx K. Student Assessment Tool.docx L. Debrief Checklists.docx [file mep-12-10479-s001.zip › I. Trainee Simulation Information Cards.doc]

**Teaching Medical Students Patient Hand offs in Obstetrics and Gynecology: A Curriculum and Assessment Tool**

**Appendix I: Trainee Simulation Information Cards**

These cards are intended to provide updates to the learner during the simulation. They can be printed, laminated and handed to the learner at the appropriate time in the role-play, or the information can be integrated into the role-play and given to the learner by the RN actor.

Patient Susan M. Quinn.

Medical Record: 987-65-432

Card 1: Door Chart Information

30 yo G2P0 LMP 10 weeks ago, bleeding since this morning

Medications: Prenatal vitamin, iron

ALL: NKDA

Patient Susan M. Quinn.

Medical Record: 987-65-432

Card 2: Brief Physical Exam

Initial Vital signs:

Blood Pressure 120/70; Heart Rate100; Respirations18; Temp37.0; O2 saturation 99%

Heart exam: regular rate and rhythm, no murmur noted

Lung exam: clear to auscultation

Abdomen: soft, fundus palpable to the umbilicus, mildly tender above

symphysis, no costo-vertebral angle tenderness

Genitalia: Normal external female genitalia

Blood at the introitus

Patient Susan M. Quinn.

Medical Record: 987-65-432

Card 3: Pelvic Exam

Pelvic:

Normal external female genitalia, no trauma or lesions

Blood at the introitus

100 mL blood clot in the vaginal vault

Cervix open, active bleeding from the os

Uterus enlarged approximately to 14 weeks’ size, irregular contour

Ovaries non palpable

Repeat Vital Signs: BP 100/60 Heart Rate 110, Respirations 18 O2 saturation 99%

Patient Susan M. Quinn.

Medical Record: 987-65-432

Card 4 Laboratory Results

Patient’s boyfriend is waiting outside, asking what is going on

Laboratory Results:

Complete Blood Count: White Blood Cells 14.6K, Hemoglobin 5.3, Hematocrit 15.7%, Platelets 147, 000

Coagulation Studies: PT/PTT 11.9/34 INR 1.8

Fibrinogen: 187

Blood Type O negative

Patient Susan M. Quinn.

Medical Record: 987-65-432

Card 5 Repeat Physical Exam

Repeat Vital Signs

BP106/70	HR 110

BP 85/56 HR 130

Abdomen: soft, tender over fundus, no rebound or guarding

Pelvic: large blood clot 500mL in vaginal vault

Cervix: apparent fetal membranes visible at os

Patient Susan M. Quinn.

Medical Record: 987-65-432

Card 6 Hospital Systems Information

OR: extension 72411

Attending: extension 72295

Code Blue: extension 21212

Blood Bank: extension 72205
